# Phytotoxicity and Other Adverse Effects on the In Vitro Shoot Cultures Caused by Virus Elimination Treatments: Reasons and Solutions

**DOI:** 10.3390/plants10040670

**Published:** 2021-03-31

**Authors:** Katalin Magyar-Tábori, Nóra Mendler-Drienyovszki, Alexandra Hanász, László Zsombik, Judit Dobránszki

**Affiliations:** 1 Centre for Agricultural Genomics and Biotechnology, Faculty of the Agricultural and Food Science and Environmental Management, University of Debrecen, P.O. Box 12, H-4400 Nyíregyháza, Hungary; dobranszki@freemail.hu; 2 Research Institute of Nyíregyháza, Institutes for Agricultural Research and Educational Farm (IAREF), University of Debrecen, P.O. Box 12, H-4400 Nyíregyháza, Hungary; mendlernedn@gmail.com (N.M.-D.); zsombik@agr.unideb.hu (L.Z.); 3 Kerpely Kálmán Doctoral School of Crop Production and Horticultural Sciences, University of Debrecen, Böszörményi Str. 138, H-4032 Debrecen, Hungary; hanaszalexandra@gmail.com

**Keywords:** virus eradication, chemotherapy, cryotherapy, electrotherapy, meristem culture, thermotherapy

## Abstract

In general, in vitro virus elimination is based on the culture of isolated meristem, and in addition thermotherapy, chemotherapy, electrotherapy, and cryotherapy can also be applied. During these processes, plantlets suffer several stresses, which can result in low rate of survival, inhibited growth, incomplete development, or abnormal morphology. Even though the in vitro cultures survive the treatment, further development can be inhibited; thus, regeneration capacity of treated in vitro shoots or explants play also an important role in successful virus elimination. Sensitivity of genotypes to treatments is very different, and the rate of destruction largely depends on the physiological condition of plants as well. Exposure time of treatments affects the rate of damage in almost every therapy. Other factors such as temperature, illumination (thermotherapy), type and concentration of applied chemicals (chemo- and cryotherapy), and electric current intensity (electrotherapy) also may have a great impact on the rate of damage. However, there are several ways to decrease the harmful effect of treatments. This review summarizes the harmful effects of virus elimination treatments applied on tissue cultures reported in the literature. The aim of this review is to expound the solutions that can be used to mitigate phytotoxic and other adverse effects in practice.

## 1. Introduction

The phytopathogen viruses can cause very significant economic losses in crop yield and quality [1,2]. In vegetatively propagated plants (tuberous plants, bulbs, fruits, etc.), they can be of particular importance, as they are more likely to be passed on to the offspring with the propagating material [3,4,5].

Viruses are very simple “organisms”, and to this day there is a debate about whether we can consider them to be living things at all [6]. They are very small in size (mostly 5–300 nm), and are constructed of a hereditary material (nucleic acid template molecule(s)) that is in general protected by a protein/lipoprotein envelope. Viruses are obligate parasites [7].

Due to the failure of own specific metabolic processes (protein synthesis, etc.), there is no pesticide to control viruses [8,9]. Cultivation of resistant cultivars may be one solution to the problem [2], but in the case of vegetatively propagated plant species, the planting of virus-free propagating material is the most effective method of control [10].

Virus elimination in general occurs under in vitro conditions based on meristem isolation. Production of planting material of vegetatively propagated crops is worldwide based on the micropropagation of virus- and pathogen-free in vitro shoot cultures [5,11], which can originate from non-adventitious (axillary or apical) or adventitious buds [12,13]. Beside organogenesis the micropropagation can also occur by somatic embryogenesis [11,14].

During the virus elimination process, it is possible to separate the virus-free part (meristematic region of shoot/root tips) from the virus-infected plant parts, since the distribution of viruses in the plant is not uniform [15,16]. Many viruses are not found in the apical dome of meristem [17], and their presence in the meristematic region can be affected by exogenous (environmental conditions) and endogenous (developmental stage of plants) factors [18]. At least four reasons are known why meristem can be free of some viruses, as follows:

(1) Intensive metabolic processes and the raised auxin concentration accompanying active cell division in meristems inhibit viral replication as well [9].
(2) The RNA silencing involved in the plant defense mechanism against viruses [19], could also play an important role in prevention of accumulation of viruses and viroids in the apex [20,21,22].
(3) The spread of viruses in plants from cell to cell is relatively slow, and their long-distance movement in plant mostly occurs via the vascular system [7] that does not exist in the meristem yet [23,24,25].
(4) Wang et al. [26] supposed a relationship between the presence of viruses and the plasmodesmata development, since they observed a few (non-branched) plasmodesmata in the cell walls of tissues where the virus was not detected, while they occurred frequently in the tissues infected by virus such as the base of first leaf primordium.

Meristem isolation and culture are often combined with other treatments (thermo- and chemotherapy) to increase the effectiveness of virus eradication [27,28]. There are other methods to obtain virus-free plantlets (electrical treatment, cryotherapy, embryogenesis) [29,30,31,32,33,34].

Even though the environmental condition for in vitro culture is highly controlled, the explants and developing plantlets suffer from several stress factors. These factors are injury occurring during meristem isolation, incomplete plant development (i.e., shoot without root), high humidity and high level of gases in vessel, supra-optimal levels of nutrients, sucrose and growth regulators in medium, etc. [35,36]. Virus elimination treatments applied in tissue culture can result in further stress effects including high or very low temperature and toxicity of chemicals leading to changes in physiology and/or morphology of plantlets [36]. Apart from inhibited growth of in vitro shoots, hyperhydricity is one of the most common morpho-physiological anomalies in in vitro cultures as a response to inappropriate environmental factors, i.e., high humidity, inadequate plant growth regulator (PGR) levels, etc. [35].

This is a review of literature on phytotoxicity and other harmful effects of the plant viral elimination methods applied on in vitro cultures. We have considered only articles that also reported the damages and phytotoxic effects of treatments, including the rate of survival, regeneration capacity, etc. We investigated the reasons for the harmful effects of the treatments and solutions to reduce losses during the process.

## 2. Meristem Excision and Culture

### 2.1. The Background

Usually the apical meristem of the shoot is very small (about 100 µm in diameter), dome shaped, and consists of about 800–1200 cells. However, the size and the shape are various depending on the species and developmental stage of plant. The shoot apex includes also the other surrounding tissues of the meristems and organs found at the distal end of the shoot [37].

Localization of viruses in the plant varies depending on the virus and plant species infected. For example, the Apple stem grooving virus (ASGV) was detected in the whole shoot tip of apple (*Malus domestica* Borkh.), even in the youngest region [38]. Wang et al. [26] found that the Raspberry bushy dwarf virus (RBDV) in raspberry (*Rubus ideaus* L.) existed in all tissues including leaf primordia (LP) and the base of meristem; virus did not present only in the least differentiated cells of the apical dome (AD). However, the smallest part of meristem, which could be mechanically isolated, was the 0.1 mm in length with the youngest leaf primordium in the case of raspberry (*Rubus idaeus* L., ‘Z13’) [26].

Excision and culture of meristem in this size (0.1–0.3 mm) are feasible only under sterile conditions, because the wounded area is too large in relation to the volume of explant, and any kind of contamination would be fatal for it [39]. Even under sterile conditions, the loss due to contamination can be very high in overly susceptible species, as there are many small sites in phylotaxis or they may be contaminated with soil [40]. It seems that normal plant development requires an apical shoot tip containing at least 1–2 leaf primordia that can ensure the production of auxins and cytokinins [9].

Even though the ability of meristems to survive was proven to be better under drought [35], freezing [41], or heat [26] stress than other tissues (leaves, cotyledons), the excised meristem suffers from several stresses during isolation and cultivation. The wounding accompanied by excision of meristem resulted in a stress similar to that caused by herbivorous insects, and reactive oxygen species (ROS) are produced in a very short time, which damages the meristems [35,39]. Browning of explants may also be a serious problem in species producing polyphenols [9]. Polyphenol oxidase (PPO) is one of the enzymes responsible for wounding induced browning of explant, which can even lead to death of those [42]. In wounded cells, PPO and its substrate (polyphenols) are released from the vacuoles and from plastids/chloroplast, where they are localized. Polyphenols can react with molecular oxygen; this reaction is catalyzed by PPO, and finally dark pigment (melanin) will be produced. Antioxidant enzymes (peroxidases (POX), superoxide dismutase (SOD)) also play role in explant browning [43].

In addition to wounds, chemical stress also affects the plant during the disinfection process using standard chemicals such as sodium hypochlorite, ethanol, and mercuric chloride [3,35]. However, both the extent of wound damage and the contamination can be reduced by use of hypodermic needle for meristem isolation [44,45,46,47]. Mother plant can be pre-treated by fungicide and bactericide chemicals in order to decrease the rate of contamination of shoot tips; thus, a less harmful disinfection procedure can be used [9].

Although shoot initiation could be observed after about 10 days in sweet potato (*Ipomoea batatas* (L.) Lam) [44,47] or 7–21 days in summer squash (*Cucurbita pepo* L., ‘Bulum’, ‘Rumbo’) [48], even several months can be taken for obtaining fully-developed healthy plantlets, during which time the in vitro cultures may become contaminated or the medium may dry out [3]. In vitro plant regeneration from isolated meristem/shoot tip takes place through organogenesis, as a response to wounding; in general, new organs or whole plantlets can develop. Even though plant cells have a very strong regeneration capability due to their developmental flexibility [49], the survival and regeneration rate of explants are significantly different, which depends on several factors.

### 2.2. Effect of the Meristem Size on the Regeneration Ability of Explant

The size of the excised part is crucial: as the size of the excised meristems increased from less than 0.1 to 0.5 mm gradually, the regeneration rates increased from 10–20% up to 44–50% in cardamom (*Amomum subulatum* Roxb.) [50] (Table 1). Similarly, survival rates of carnation (*Dianthus caryophyllus* L.) shoot meristems increased from 20% to 80% with an increase of explant size from 0.1 to 0.4 mm [51]. Wang et al. [26] also detected positive correlation between the shoot tip length of raspberry (*Rubus idaeus* L., ‘Z13’) and its survival rate and regeneration capacity. The explant excised in 0.1 mm survived in 25%, and 40.0% of them regenerated to shoots, while 40% of shoot tips in 0.2 mm length survived with 65% regeneration rate. Significantly more shoots (95%) survived in the case of the 0.3 mm shoot tip, and each of them was able to regenerate the shoot. Similarly, sweet potato (*Ipomoea batatas* (L.) Lam.) meristems in the length of longer than 0.3 mm were only able to survive [52].

Meristems between 1 and 2 mm in size showed at least 79.2% regeneration rate with 100% virus elimination rate in the case of both Sugarcane mosaic virus (SCMV) and Sugarcane yellow leaf virus (ScYLV) in sugarcane (*Saccharum* spp. L., ‘NCo376’) virus elimination experiments. Plantlets developed from the larger shoot apex remained all infected by viruses, while smaller explants responded with shoot development only at a 46.4–53.9% rate [53]. Meristems 0.3–0.6 mm in size were proven to be the most sui0le for the establishment of in vitro culture in apple (*Malus domestica* Borkh.), although some shoots initiated from explants sized 0.5–0.6 mm remained infected by Apple chlorotic leaf spot virus (ACLSV). A high rate of loss (44.4%) could be observed in the case of meristems smaller than 0.3 mm, due to their dehydration, while the culture of meristem explants excised in larger than 0.6 mm size failed in virus removal. Moreover, the presence of phenolic browning was also more frequent in meristem explants > 0.5 mm [40]. The size of the meristem can also affect the time required for regeneration; in the meristem culture of cardamom (*Amomum subulatum* Roxb., ‘Golsahi’ and ‘Ramsahi’), the shoot initiation occurred within 14–18 weeks on explants larger than 0.3 mm, while regeneration on smaller meristems could be observed after 18–24 weeks [50].

The size of isolated meristem significantly affected the shoot length in potato (*Solanum tuberosum* L.): meristems were excised in 100, 200, and 300 µm, and the length of shoots in the average of two cultivars were 5.2, 7.2, and 9.7 cm, respectively, after 60 days of culture initiation [54]. The optimal size of the meristem depended on the genotype in the case of fig (*Ficus carica* L.) varieties. The highest regeneration rates (96%) were achieved by isolation of meristem in 0.5 mm length in the case of Capri fig ‘Assafri’. Two of the Tunisian local figs showed the best results when a meristem of 1.0 mm length was used for culture in the case of ‘Zidi’ and ‘Bither Abiadh’, resulting in 79% and 73.3% regeneration rates, respectively. The third Tunisian local fig (‘Soltani’) showed the highest regeneration rate (95.2%) when meristem of 1.5 mm length was isolated [55]. However, it was observed in each variety that culture initiation by larger shoot apex (1.5 mm) was accompanied by death of the explant base in high rate (up to 76.45%, depending on genotype).

### 2.3. Effect of the Genotypes, Explant Source, and Age on the Regeneration Ability of Isolated Meristems

The survival of the meristem explants and the success of shoot regeneration was still also affected by many factors, including the genotypes and physiological stage of the mother plant, or other environmental factors such as plant growth regulators (PGR), nutrient supply, light condition, etc., reviewed by Bidabadi and Jain [56]. Interaction between the factors can often be detected; however, one of the most important factors proved to be the PGR content of initiation medium, e.g., in sweet potato (*I. batatas* (L.) Lam) [57] and in grapevine (*Vitis vinifera* L.) [58].

The regenerative capacity of genotypes can be very different; for example, in potato (*S. tuberosum* L.), it varied between 40% and 80% [59]; in summer squash (*Cucurbita pepo* L.) between 69% and 75% [48]; and in fig (*Ficus carica* L.) between 51.7–78.3% [60]. Other factors, however, can also play role in the rate of responsiveness, as was observed when 100 genotypes of garlic (*Allium sativum* L.) were involved in virus elimination experiments based on meristem isolation. Although very high regeneration rates (90–100%) were observed in garlic meristems in length of 1.0 mm (2–3 leaf primordia), when 0.3–0.8 mm sized meristems were isolated, their survival and regeneration rate varied between 1% and 80%, and six genotypes did not regenerate the shoot at all [61].

In another experiment, 10 genotypes of tested 51 garlic accessions showed better results on PGR-free medium, while the others performed better on media containing PGRs (0.1 mg/L β-indolyl-acetic acid (IAA) + 0.1 mg/L kinetin (KIN) or 0.01 mg/L α-naphthalene acetic acid (NAA) + 0.01 mg/L 6-benzyladenine (BA)), and the majority of them preferred the latter medium [61]. Although the same PGRs were used for meristem cultures of fig (*F. carica* L.), the best levels of them (BA and gibberellic acid, GA_3_) were different for fig cultivars [60].

**Table 1:** Results and details of meristem cultures experiments.

Plant Species, Cultivar, Virus	Methods	Survival and/or Regeneration [Reference]
Fig, *Ficus carica* L., ‘Bursa Siyahi’, ‘Alkuden’, FMV	Meristems (0.5–0.8 mm) were in D for 1 wk, transfer weekly on MS with various PGR combinations (mg/L): A: 0.1 GA_3_ + 0.2 BA + 0.1 IBA; B: 0.1 GA_3_ + 0.5 BA + 0.1 IBA, C: 0.2 GA_3_ + 0.2 BA + 0.1 IBA, D: 0.2 GA_3_ + 0.2 BA + 0.1 IBA, for 8 wks, transfer to MS with various PGR for shoot development: A: 0.1 GA_3_ + 1.0 BA + 0.1 IBA; B: 0.1 GA_3_ + 2.0 BA + 0.1 IBA, C: 0.2 GA_3_ + 1.0 BA + 0.1 IBA, D: 0.2 GA_3_ + 2.0 BA + 0.1 IBA. Rooting on MS: 1: 0.1 GA_3_ + 0.0 IBA; 2: 0.1 GA_3_ + 1.0 IBA, 3: 0.1 GA_3_ + 2.0 IBA, 4: 0.0 GA_3_ + 0.0 IBA.	Survival rates on A/B/C/D: ‘Bursa Siyahi’: 73.3%/73.3%/80%/86.7%, ‘Alkuden’: 73.3%/40%/46.7%/46.7%. Shoot development on A/B/C/D: ‘Bursa Siyahi’: 44.4%/63.9%/58.9%/70%, ‘Alkuden’: 63.9%/70%/44.4%/50%. Rooting rate/root number on A/B/C/D: ‘Bursa Siyahi’: 66.6%/6.3; 44.4%/5.3; 44.4%/4.3; 22.2%/1.6. ‘Alkuden’: 44%/30; 83.3%/40; 33.4%/0.7; 16.7%/1.3. [60]
Raspberries, *Rubus idaeus* L., ‘Z13’, RBDV	Meristems of 0.1 mm (1LP), 0.2 mm (2LP), 0.3 mm (2LP) cultured for 3 days on solid MS with 100 mg/L myo-inositol, 30 g/L sucrose, 0.5 mg/L BA, 0.05 mg/L IBA, 3.5 g/L Bacto agar, 1.2 g/L Gelrite, and 2.5 g/L AC for 3 ds, then transfer to the same medium without AC. Culture at 22 ± 2 °C, 16 h L., 45 µE s^−1^ m^−2^	Survival/regeneration rates: 0.1 mm: 25%/40%; 0.2 mm: 40%/65%; 0.3 mm: 95%/100%. [26]
Grapevine, *Vitis vinifera* L., ‘Flame Seedless’, GLRaV-1, GFLV	Meristems (0.5 mm, 1.0 mm with 2 LP), on WP without PGR or with 0.5, 1.0 or 1.5 mg/L BA, 0.04 mg/L IBA. Culture for 2 wks at 25 ± 2 °C, 16 h L. Then sub-culture: 4 wks.	Shoot number per explant: 0.5/1.0 mm explant on different BA (mg/L): BA 0: 0.8/1.0; BA 0.5: 3.7/6.8; BA 1.0: 5.8/12.2; BA 1.5: 5.3/13.1 in GFLV infected plants. BA 0: 0.9/1.0; BA 0.5: 3.9/6.2; BA 1.0: 5.8/10.1; BA 1.5: 7.3/12.8 in GLRaV-1 infected plants. Shoot length (cm): 0.5/1.0 mm explant on different BA (mg/L): BA 0: 6.4/8.5; BA 0.5: 8.9/11.6; BA 1.0: 9.3/10.4; BA 1.5: 9.8/10.9 in GFLV infected plants. BA 0: 5.3/8.9; BA 0.5: 7.7/11.5; BA 1.0: 8.2/9.6; BA 1.5: 7.1/8.5 in GLRaV-1 infected plants. [58]
Sugarcane, *Saccharum* spp. L., ‘NCo376’, SCMV, ScYLV	AP meristems in sizes from 0.5 to 10 mm on the liquid MS with 20 g/L sucrose, 10 g/L agar, 3.5 g/L AC, 1 mg/L methylene blue. PGR treatment: A: 2 mg/L BA, 1 mg/L KIN, 0.5 mg/L NAA; B: 0.5 mg/L BA; C: 2 mg/L BA; D: 0.1 mg/L BA, 0.015 mg/L KIN. Culture in D for 1 wk, then16 h L., 28 °C, after 1 wk sub-culture on medium without AC. Shoot proliferation on liquid MS medium with 0.1 mg/L BA. Sub-cultures: fortnight. Shoots (4 cm) rooted in ½ MS with 5 g/L sucrose, 8 g/L agar, 0.25 g/L casein-hydrolysate, for 2–3 wks.	Regeneration rate of different sized meristems, explants from field/node shoot. ≤1 mm: 46.4%/53.9%; >1 ≤2 mm: 79.2%/100%; >2 ≤10 mm: 69.2%/100%. Regeneration rates/shoot number on different PGR: A: 50%/5.9; B: 55%/4.1; C: 100%/3.8; D: 100%/11.1. [53]
Summer squash, *Cucurbita pepo* L., ‘Bulum’, ‘Rumbo’, ZYMV, CMV, AMV, BYMV	Meristem 0.3 mm, from 25–30 ds old shoot onto filter paper bridge on liquid MS with various PGR content: KIN or BA (0.5/1.0/1.5/2.5 mg/L), or 0.5 mg/L NAA with KIN (1.0/1.5/2.5 mg/L), or 0.5 mg/L GA_3_ with KIN (1.5/2.0/2.5 mg/L), or GA_3_ (0.5–2.0 mg/L). Culture at 25 ± 2 °C, 16 h, 2000–3000 lux, for 28 ds. Then onto MS with 8.0 g/L agar, and combinations of BA, KIN, IBA, IAA.	Regeneration rates: ‘Bulum’/‘Rumbo’: Control: 14.4%/11.3%, best results: 2.0 mg/L KIN + 0.5 mg/L GA_3_: 75.6%/69.3%. Shoot length (cm): ‘Bulum’/’Rumbo’: Control: 3.1/2.97, best results: 2.0 mg/L KIN: 4.7/4.24. Number of roots: ‘Bulum’/‘Rumbo’: Control: 2.9/2.8, best results: 1.0 mg/L BA: 3.4/3.3. Number of shoots (42 ds): ‘Bulum’/’Rumbo’: Control: 2.6/2.5, best results: BA 2.0 mg/L: 4.8/4.1. [48]
Okra, *Abelmoschus esculentus* L. (Moench.), ‘Parbhani Kranti’, ‘SL-444’, OMV, YVMV	Meristems 0.3–0.5 mm on filter paper bridge on liquid MS with combinations of BA: (0.1; 0.5; 1.0; 1.5; 2.0 mg/L) and GA_3_ (0.1; 0.5 mg/L) or NAA (0.1; 0.5 mg/L). Culture for 3–4 wks. Then sub-cultured on MS with various PGR (+8 g/L agar). Micropropagation from nodal segments. Rooting on MS with NAA or IBA (in 0.5, 1.0, 2.0 or 3.0 mg/L). Culture conditions: 24 ± 1 °C, 16 h L., 28–34 µmol m^−2^ s^−1^.	Survival results on PGRs (mg/L): ‘Parbhani Kranti’/‘SL-444’: BA 0.1: 32.7/28.8%; BA 0.5: 7.9/45.8%; BA 1.0: 72.3/67.4%; BA 1.5: 58.2/52.5%; BA 2.0: 40.74/35.9%. BA 0.5 + GA_3_ 0.1: 49.4/42.6%; BA 1.0 + GA_3_ 0.1: 53.5/47.3%; BA 1.0 + GA_3_ 0.5: 60.3/58.8%; BA 1.5 + GA_3_ 0.5: 55.6/50.5%; BA 0.5 + NAA 0.1: 40.2/38.1%; BA 1.0 + NAA 0.5: 56.5/51.6%; BA 1.5 + NAA 0.5: 50.7/45.9%. Best multiplication rates: on 1.0 mg/L BA + 0.5 mg/L GA_3_: ‘Parbhani Kranti’ 8.9 shoots/explant, ‘SL-444’: 6.8 shoot/explant. [46]
Sweet potato, *Ipomoea batatas* (L.) Lam), ‘Awassa local’, ‘Awassa-833’, ‘Guntute’, SPFMV, SPCSV	Meristems on MS with 30 g/L sucrose, 7 g/L agar, and 13 PGR combinations (GA_3_, NAA, and BA). Culture at 24 ± 2 °C, 12 h L., 40 µmol m^−2^ s^−1^. Sub-culture: 4 wks.	The best regeneration rates were: (1): 66.7% on medium with 1 mg/L BA, 0.01 mg/L NAA, and 1 mg/L GA_3_ in ‘Awassa-833’ and in ‘Guntute’. (2): 63.33% on medium with 1 mg/L BA, 0.01 mg/L NAA, and 2 mg/L GA_3_ in ‘Awassa local’. The highest number of shoots per explant: ‘Awassa-833’: 5.26, ‘Awassa local’: 5.12 both on medium with 2 mg/L BA. ‘Guntute’: 2.5 on medium with 3 mg/L BA. [57]
Carnation, *Dianthus caryophyllus* L., CLV, CarVMV	Meristems in sizes of 0.1; 0.2; 0.3; and 0.4 mm with 1–2 LP, cultured on MS with 0.1 mg/L NAA, 2.0 mg/L KIN, grown at 25℃, 16 h L. Shoot clump proliferation on MS with 30 g/L sucrose, 8 g/L agar, 0.2 mg/L BA. Multiplication on MS with 1.0 mg/L BA, 0.5 mg/L KIN, sub-culture: for 3wks. Rooting: MS with 1.5 mg/L NAA.	Survival rates of meristem in size of 0.1/0.2/0.3/0.4 mm with 1–2 LP: 20%/35%/65%/80%. [51]
Potato, *Solanum tuberosum* L., ‘Burren’, ‘Binella’, PVY	AP meristems (100, 200, 300 μm) cultured on MS, with 2 mg/L glycine, 5 mg/L nicotinic acid, 5 mg/L pyridoxine, 5 mg/L thiamine, 5 mg/L ascorbic acid, 200 mg/L myo-inositole, 2.0 mg/L GA_3_, 0.2 mg/L KIN, 3% sucrose, 0.6% agar. Culture: 25 ± 2 °C, 16 h, 2.5 μmol m^−2^ s^−1^.	Survival rates of 100/200/300 μm meristems: ‘Burren’: 88%/100%/100; ‘Binella’: 86%/94%/100%. Shoot length (cm) after 60 ds: ‘Burren’: 5.4/7.7/9.9; ‘Binella’: 4.9/6.6/9.6. [54]
Sweet potato, *Ipomoea batatas* (L.) Lam., ‘Bellela’, ‘Temesgen’, ‘LO-3233’, ‘Zapallo’, SPCSV, SPFMV, SPMMV, SPCFV, SPCaLV, SPMSV, SwPLV, SPVG, CMV	Meristems 0.5–0.7 mm on MS with 30 g/L sucrose, BA (0.1; 0.5; 1.0; 2.0; 5.0 mg/L) combined with 0 or 0.01 mg/L NAA, and 0 or 1.0 mg/L GA_3_. Culture at 25 ± 2 °C, 16 h L., 51 μmol m^–2^ s^–1^. Sub-culture on same medium 4 wks. Multiplication: MS with PGR combinations: KIN, BA, IAA. Rooting: ½ MS with 0, 1, 2, 3, 4, or 5 mg/L IBA.	Regeneration rates of ‘Bellela’/‘Temesgen’/‘LO-3233’/‘Zapallo’ on medium without PGR: 5.4/17.1/13.0/21.6%, on medium with 0.01 mg/L NAA + 1.0 mg/L GA_3_ + 0.1 mg/L BA: 6.7/30/20/30%; or +0.5 mg/L BA: 63.3/53.3/40/16.7%, or +1.0 mg/L BA: 63.3/70/60/70%; or +2.0 mg/L BA: 73.3/93.3/90/80%; or +5.0 mg/L BA: 100/100/76.6/70%. The best shoot proliferation on MS + 0.5 mg/L BA + 0.5 mg/L KIN. The best rooting was on PGR-free medium. [44]
Sweetpotato*,* *Ipomoea batatas* (L.) Lam., ‘BARI-11’, ‘BARI-22’, ‘BARI-33’, ‘BARI-44’, ‘BARI-55’, ‘BARI-66’, ‘BARI-77’, SPFMV, SPMMV	AP meristems (0.3–0.5 mm, 1–2 LP) on filter paper bridge, on liquid MS with combinations of KIN and GA_3_. Culture at 25 °C, 16 h L., 50–60 µmol m^−2^ s^−1^ for 4 wks. Sub-culture on semisolid medium for 4–6 wks.	Regeneration rates in a range of 7 genotypes: KIN 1.0 mg/L: 37.5–50%; KIN 2.0 mg/L: 45.8–66.7%, KIN 2.5 mg/L: 54.7–70.8%; KIN 3.0 mg/L: 41.7–58.3%; GA_3_ 1.0 mg/L: 33.3–45.8%; GA_3_ 1.5 mg/L: 41.7–54.2%; GA_3_ 2.0 mg/L: 45.8–62.5%; GA_3_ 3.0 mg/L: 37.5–50%; KIN 2.0 + GA_3_ 0.1 mg/L: 54.2–66.7%; KIN 2.0 + GA_3_ 0.5 mg/L: 62.5–79.2%; KIN 2.5 + GA_3_ 0.1 mg/L: 50–62.5%; KIN 2.5 + GA_3_ 0.5 mg/L: 54.2–75%. [47]
Fig, *Ficus carica* L., ‘Zidi’, ‘Soltani’, ‘Bither Abiadh’, ‘Assafri’, FMD	ST (0.5, 1.0, and 1.5 mm) on MS with 30 g/L sucrose, 7 g/L agar, 90 mg/L PG. PGRs: (M1): 0.2 mg/L BA, 0.1 mg/L NAA, 0.1 mg/L KIN; (M2): 0.2 mg/L BA, 0.1 mg/L NAA, 0.1 mg/L IPA; (M3): 0.2 mg/L BA, 0.1 mg/L NAA, 0.1 mg/L GA_3_, (M4): 0.2 mg/L BA 0.1 mg/L 2,4-D. Culture at 25 ± 1 °C, 16 h L., 40 μmol m^−2^ s^−1^	Regeneration rates of different sized meristems: 0.5/1.0/1.5 mm: ‘Zidi’: 61.1%/79%/70.5%; ‘Bither Abiadh’: 67.8%/73.3%/56.7%; ‘Soltani’: 90%/55.7%/95.2%; ‘Assafri’: 96%/92.6%/87.96%. [55]
Large Cardamom, *Amomum subulatum* Roxb., ‘Golsahi’, ‘Ramsahi’, CBDV, LCCV	Meristems 0.2–0.7 mm on MS with 30 g/L sucrose and various PGRs: BA, 0.5–1.0 mg/L, GA_3_, 0.1 mg/L, IBA or NAA 0.01–0.1 mg/L, or IAA, 0.12–0.15 mg/L, PVP, 0.5 g/L or AA 100 mg/L, 7 g/L agar, for 6 wks. Then transfer to same MS. Sub-culture: MS with PGRs: BA (0.5–1.0 mg/L), IBA (0.01–0.1 mg/L), and GA_3_ (0.1–0.5).	Survival rates of meristems: 0.2–0.3 mm: 20.7%; 0.3–0.4 mm: 25.7%; 0.4–0.5 mm: 32.1%; 0.5–0.6 mm: 32.9%; 0.6–0.7 mm: 36.4%. Survival rates on medium with different PGR content: (1): 1.0 mg/L BA + 0.05 mg/L IBA + 0.1 mg/L GA_3_: 56.6%; (2):0.5 mg/L BA + 0.08 mg/L IBA + 0.1 mg/L GA_3_: 37.5%; (3): 0.5 mg/L BA + 0.58 mg/L NAA + 0.1 mg/L GA_3_: 9.5%. [50]
Potato, *Solanum tuberosum* L., 8 cultivarsPVY, PVM, PVS, PVX	Meristems on liquid MS with 20 g/L sucrose, 1 g/L casein, 0.1 mg/L IBA, 1 mg/L GA_3_, and 40 mg/L adenine hemisulphate. Culture at 20 ± 2 °C, 16 h L., 50 μmol s^−1^m^−2^. Sub-culture: 3 wks (2×). Then transfer to MS with 30 g/L sucrose, 1 g/L casein, 0.5 mg/L IBA, 9 g/L Bacto agar.	Regeneration rates: ‘Truls’: 70%, ‘Kerrs Pink blatt skall’: 60%, ‘Gammelraude’: 60%, ‘Abundance’: 50%, ‘Gjernespotet’: 40%, ‘Hroar Dege’: 75%, ‘Iverpotet/Smaragd’: 80%; ‘Sverre’: 75%. [59]

2,4-D: 2,4-Dichlorophenoxyacetic acid, AA: ascorbic acid, AC: activated charcoal, ACLSV: Apple chlorotic leaf spot virus, AMV: Alfalfa mosaic virus, AP: apical, ApMV: Apple mosaic virus, ASGV: Apple stem grooving virus, ASPV: Apple stem pitting virus, BA: 6-benzyladenine, BYMV: Bean yellow mosaic virus, CarVMV: Carnation vein mottle virus, CBDV: Cardamom bushy dwarf virus, CLV: Carnation latent virus, CMV: Cucumber mosaic virus; d(s): day(s), D: darkness, FMD: Fig mosaic disease, FMV: Fig mosaic virus, GA_3_: gibberellic acid, GFLV: Grapevine fanleaf virus, GLRaV-1: Grapevine leafroll-associated virus, h(s): hour(s), IAA: β-indolylacetic acid, IBA: Indole-3-butyric acid, IPA: Iso-Pentyl Adenosine, KIN: kinetin, L: light, LCCV: Large cardamom chirke virus, LP: leaf primordium, MS: Murashige-Skoog medium [62], NAA: α-naphthylacetic acid, OMV: Okra mosaic virus, PG: phloroglucin, PGR(s): plant growth regulator(s), PNRSV: Prunus necrotic ringspot virus, PVM: Potato virus M, PVP: polyvinylpyrrolidone, PVS: Potato virus S, PVX: Potato virus X, PVY: Potato virus Y, RBDV: Raspberry bushy dwarf virus, SCMV: Sugarcane mosaic virus, ScYLV: Sugarcane yellow leaf virus, SPCaLV: Sweet potato caulimo-like virus, SPCFV: Sweet potato chlorotic fleck virus, SPCSV: Sweet potato chlorotic stunt virus, SPFMV: Sweet potato feathery mottle virus, SPMMV: Sweet potato mild mottle virus, SPMSV: Sweet potato mild speckling virus, SPVG: Sweet potato virus G, ST: shoot tip, SwPLV: Sweet potato latent virus, wk(s): week(s), WP: Woody Plant medium [63], YVMV: Yellow vein mosaic virus, ZYMV: Zucchini yellow mosaic virus.

### 2.4. Effect of the Medium Component on the Regeneration Ability of Explants

Very low rate of shoot initiation (5.4–21.6% depending on genotypes) could be achieved from sweet potato (*Ipomoea batatas* (L.) Lam.) meristems on medium without PGRs [57], while Kaushal et al. [45] did not observe any organogenesis on gentian (*Gentiana kurroo* Royle) explants cultured on medium lacking PGRs. Similarly, Anisuzzaman et al. [46] could not detect any development on okra (*Abelmoschus esculentus* L. (Moench.)) meristems cultured on medium without any PGR. Application of NAA and GA_3_ alone failed to induce shoot development from meristem explants of ‘Brondal’ sweet potato (*I. batatas* (L.) Lam.) cultivar, while addition of 1 mg/L BA alone to medium induced the shoot regeneration at about a 1% rate. The presence of BA either alone or combined with NAA or GA_3_ was proven to be necessary for shoot initiation [64]. BA level was crucial to increase the shoot initiation percentage of other cultivars of sweet potato (*I. batatas* (L.) Lam.), which increased as BA level increased from 0.1 to 2.0 mg/L (‘Lo-323’ and ‘Zapallo’) or to 5.0 mg/L (‘Belella’ and ‘Temesgen’). Although 100% regeneration rate were observed in ‘Belella’ and ‘Temesgen’ cultured on medium with 5.0 mg/L BA, the shoots were dwarf and highly multiplied. In the case of ‘Lo-323’ and ‘Zapallo’ genotypes, application of 2.0 mg/L BA resulted in the best shoot initiation rates (90% and 80%, respectively) and shoots were of good quality [44]. Lower level of BA (1.0 mg/L) was found to be optimum for ‘Awassa-83’, ‘Guntute’, and ‘Awassa local’ sweet potato (*I. batatas* (L.) Lam.) cultivars, which resulted in shoot induction at more than 60.0% of the rate of isolated meristems [57]. High regeneration rate (83%) could be achieved by using 1.0 mg/L BA and 0.5 mg/L IAA for culture of gentian (*G. kurroo* Royle) meristems [45]. The same level of BA applied alone as PGR resulted in the best survival rates (72% and 67%) in meristem cultures of both okra (*A. esculentus* L. (Moench.)) genotypes ‘Parbhani Kranti’ and ‘SL-44’ [46]. In meristem cultures of grapevine (*Vitis vinifera* L.) with increase of both cytokinins (BA and KIN) level (from 0.0 to 1.0 mg/L), the amount of formed callus increased, especially in the case of BA [65]. Combination of 0.1 mg/L BA with 0.015 mg/L KIN was the best in the shoot induction medium also for sugarcane (*Saccharum* spp. L., ‘NCo376’) meristems, resulting in a 100% regeneration rate and high shoot number per meristem (13.7), while those meristems cultured on medium without KIN (0.5 and 2.0 mg/L BA) yielded less than five shoots per meristem, although their regeneration rates were different: 55% and 100%, respectively. However, the lowest regeneration response (50%) and quite a few shoots (six per explant) were detected on meristems grown on medium with a combination of 2.0 mg/L BA and 1.0 mg/L KIN, supplemented with 0.5 mg/L NAA [53]. Sweet potatoes (*I. batatas* (L.) Lam) tended to respond to unfavorable cytokinin content of the medium with undesirable callus formation: use of thidiazuron (TDZ) or BA led to callus formation and failed in shoot induction [52]. When cultivars responded to BA with abundant callus development, the KIN also could be used efficiently for meristem cultures in several sweet potato (*I. batatas* (L.) Lam) genotypes; when 2.0 mg/L KIN was added to medium with 0.5 mg/L GA_3_, the survival rates of explants varied between 62.5% and 79.2% depending on genotypes [47]. The best shoot initiation responses (75% survival rate of isolated meristems with high vigor) were obtained by application of liquid Murashige–Skoog medium (MS) [62] with 2.0 mg/L KIN and 0.5 mg/L GA_3_ without any kind of callus development [52]. The same PGR combination (2.0 mg/L KIN and 0.5 mg/L GA_3_) was the most effective for summer squash (*Cucurbita pepo* L.) regeneration (75.5% and 69.27% regeneration rates) from meristem in the case of ‘Bulum’ and ‘Rumbo’, respectively [48]. Addition of 0.5 mg/L GA_3_ to meristem culture medium containing 2.0 mg/L KIN increased the survival rate by about 14% in average of seven sweet potato (*I. batatas* (L.) Lam.) genotypes, compared those cultured on the medium without GA_3_ [47]. GA_3_ level had to be increased from 1.0 to 2.0 mg/L to achieve the best regeneration rate (63.3%) in ‘Awassa local’ sweet potato (*I. batatas* (L.) Lam.) cultivar [57]. Even a much higher level GA_3_ (up to 20 mg/L) also enhanced the shoot regeneration in the case of ‘Brondal’ sweet potato (*I. batatas* (L.) Lam.) cultivar; regardless, there were no significant differences between shoot regeneration capability of meristems cultured on media with 5.0, 10.0, and 20.0 mg/L GA_3_ content. However, the most shoots were obtained on the medium containing 10.0 mg/L GA_3_ due to multiplied shoots developed on meristems [64].

Application of more than 0.05 mg/L of NAA in the medium of sweet potato (*I. batatas* (L.) Lam) meristem culture led to formation of abundant calli without the ability of shoot regeneration [57]. Addition of 0.1 mg/L isopentyl adenosine (IPA) to the culture initiation medium containing 0.2 mg/L BA and 0.1 mg/L NAA also resulted in significantly higher rate of fig (*Ficus carica* L.) explants (58.3–81.3%), showing callus development on three of the four varieties tested, compared to the medium without IPA (20.4–62.2%). However, no callus development was observed in ‘Soltani’ cultivar on medium with IPA, this variety was also characterized by poor callus development (0–8.75%) on other media [55]. Excised sweet potato (*I. batatas* (L.) Lam.) meristems preferred the culture on the liquid medium instead of the semi-solid medium, where the majority of explants were not responsive enough, maybe due to the higher accessibility of the nutrients and water in the liquid medium compared to the semi-solid medium [52]. Application of the liquid medium was also preferred for species that suffered from polyphenolic browning, because the toxic molecules (quinone compounds) are less able to accumulate around the explants [40]. Inhibition of production of phenolic molecules by addition of 2-aminoindane-2-phosphonic acid (AIP) into the medium can reduce the rate of browning as was reported in *Artemisia annua* L., *Ulmus americana* L., and *Acer saccharinum* L. in vitro cultures [66]. Addition of antioxidants to the medium or as pre-treatment for mother plants, such as phloroglucinol (PG), ascorbic acid (AA), citric acid (CA), etc., can prevent the browning of meristem cultures [55,67,68]. Adsorption of toxic materials by application of activated charcoal (AC) in the medium also frequently used solution in tissue cultures [69]. Application of 2.0 g/L AC in culture initiation medium can also enhance the regeneration ability of meristems isolated from several grapevine (*V. vinifera* L.) cultivars [70].

Shoot length of sweet potato (*I. batatas* L. Lam) cultivars varied depending on the genotypes and BA level added to the shoot initiation medium. After three months of culture period, the longest shoots (8.8 cm) developed on ‘LO-323’ explants on medium supplemented with 0.5 mg/L BA, while similar growth was observed in ‘Belella’ (8.2 cm) on the medium with 1.0 mg/L BA. The shortest shoots developed on ‘Zapallo’ explants, where the best results (2.9 cm) were obtained by application of 1.0 mg/L BA [44]. Although significantly more shoots developed on the meristems of grapevine (*V. vinifera* L.) cultured on media supplemented with BA (0.2–1.0 mg/L) compared to those induced by media with the same levels of KIN, the shoot elongation was greatly inhibited [65]. In the case of different sized grapevine (*V. vinifera* L., ‘Flame Seedless’) meristems explants (0.5 and 1.0 mm), the number of shoots were almost the same (0.9–1.0 shoots per explant) when meristems were cultured on cytokinin-free medium. However, when they were grown on media containing BA (0.5–1.5 mg/L), significantly more shoots developed on the larger explant. Increasing the BA concentration from 0.5 to 1.0 mg/L in media also significantly increased the number of shoots, while a further increase to 1.5 mg/L no longer resulted in a further significant change. In any case, the higher number of shoots was accompanied by a decrease in the length of the shoots [58]. In the case of ‘Brondal’ sweet potato (*I. batatas* L. Lam) cultivar, the significantly longest shoots (up to 20 mm) developed on medium supplemented with 10.0 mg/L GA_3_, compared to those shoots (<14 mm) grown on media with 0.0 and 5.0 mg/L GA_3_, each medium contained also 1.0 mg/L BA, while the length of shoots regenerated on the medium with 20.0 mg/L GA_3_ did not differ from either [64]. The PGR content in the medium can be supposed to be the most determining factor affecting the rate of shoot growth from the isolated meristem.

### 2.5. Effect of the Season and In Vitro Culture Condition on the Regeneration Ability of Explants

April and May were the best seasons for the establishment of grapevine (*Vitis vinifera* L., ‘Flame seedless’) meristem culture, because the phenolic content of plant was low [58]; the beginning of summer is still appropriate for the establishment of grapevine tissue culture, during the period of rapid shoot growth [71]. Similarly, in the case of temperate trees, the spring months are best suited for starting in vitro culture when they are in the active growth phase [9,43]. After initiation cultures of plants producing polyphenols, they should be stored under dark and cool conditions for a short period, in order to reduce the activity of enzymes [9,43,60,65]. Frequent transfer to fresh medium is also required for these species [60,72]. Photoperiod also can affect the success of shoot regeneration. Murashige [73] reported that *Calanchoe* sp. regenerated better under short day illumination, which is required for its flowering, while walnuts (*Juglans* sp.) preferred a long day photoperiod for shoot proliferation.

### 2.6. Solutions for Improvement of Survival and Regeneration Ability of Explants after Meristem Isolation

Pre-treatment of the mother (donor) plant by chemicals to reduce contamination, and timing of the shoot tip/meristem collection and excision can improve the survival of explants. Technically, the use of sharp and thin tools for meristem isolation (hypodermic needle) can reduce the rate of injury. Decisions about the size of the meristem to be excised should be made considering the virus type (its localization) and plant species and cultivar; moreover, the positive correlation between the survival rate and the size of meristem should be taken into account. Application of an adequate medium for in vitro culture initiation from meristems is also necessary to enhance the responsiveness of explants and their regeneration capacity; liquid medium may be preferred, proper balance of PGRs is necessary, and the type and level of cytokinins and auxins are especially crucial. Additives applied in the initiation media such as antioxidants (AA, CA, polyvinylpyrrolidone (PVP)), or absorbents (AC) play an important role in prevention losses due to phenolic browning. Growing conditions are also to be fitted to the requirements of genotypes, and if needed, a dark and cool environment should be ensured.

## 3. Shoot Tip Cryotherapy

### 3.1. The Background

*“In fact, freezing injury is reported to be mainly the result of intracellular water crystallization, which occurs either during the cooling and/or the thawing steps”*

Helliot et al., 2002 [74]

Virus eradication by cryotherapy is also based on the separation of healthy cells (cell groups) from infected ones [75], similar to meristem isolation (Section 2 and Section 2.1). During cryotherapy, infected shoot tips or axillary buds are kept in liquid nitrogen at −196 °C for a certain period (in general for 1 h) [76,77]. Differentiated, larger, and more vacuolated cells with high water content survive the ultra-low temperature treatments at a lower rate than smaller, highly cytoplasmic meristem cells [74,76,78]. Surviving cells are generally restricted to the apical dome of meristem and the youngest leaf primordia (LP1 and LP2) [25]. As a consequence, the regenerated plants from surviving cells have a good chance of being virus-free due to different occurrence of viruses in different plant tissues (see Introduction chapter).

Other pathogens such as viroid, phytoplasma, and bacteria can be eliminated this way as well [79,80,81]. The benefit of using cryotherapy for virus elimination is that it is not necessary to isolate very small sized meristems. Moreover, it is technically easier and relatively fast to obtain virus-free plantlets, because the regeneration capability of larger explants is higher than smaller ones [78]. Cryotherapy is also a promising method for species in which the phenolic browning is a problem during the isolation of the meristem, for example, bananas (*Musa* L. spp.) [74]. Further advantages and disadvantages of the cryotherapy method are very well summarized in many recent reviews [76,79,81,82].

Different cryotherapy methods have been used for virus elimination (encapsulation–dehydration, encapsulation–vitrification, and droplet–vitrification) with similar effectiveness considering both the virus eradication and the ability of shoot tips for regrowth [81] (Table 2).

During freezing, several harmful phenomena can be detected, including membrane damage, leading to structural and compartmentation shortcomings, causing electrolytes leakage [83] and increase in electrolyte concentration [84]; finally, the cell can collapse and death may occur [76]. Freezing damage can arise during both the freezing and thawing process; it is related to the crystallization of water localized in cells [74] and dehydration of cells due to freezing of extracellular water [85]. Thus, the proper dehydration of plant parts is essential for reducing frost damage to issues. Dehydration of actively growing parts, such as shoot tips and meristems, is complicated due to their high water content [84]. Dehydration may be induced by air flow or application of desiccants [84].

Moreover, infected plants tolerate the cryo-treatment less than healthy plants, and actively growing parts, such as shoot tips, and meristems used for elimination process are more difficult to bring to a state suitable for cryo-treatment [84]. The cryo-treatment significantly decreased (by 39–50%) the regeneration rate of shoot tips compared to the untreated explants in *Prunus* rootstock (‘Fereley-Jaspi (R)’) [78]. Moreover, responses of species and genotypes, even in the same species, were found to respond very differently to the cryotherapy [76]. Survival rates are various for different species; it was very low (10%) in apricot (*Prunus armeniaca* L.) [86] while it was 76.3% in yam (*Dioscorea rotunda* Poir.) [77]. Good survival rates could be achieved in apple (*Malus domestica* Borkh) (70–75%) [87,88] and up to 85% was obtained in potato (*Solanum tuberosum* L.) [89] and in sweet potato (*Ipomoea batatas* (L.) Lam) [25]. High survival rate (90%) was observed in artichoke (*Cynara scolymus* L.) [90].

**Table 2:** Results and methodology of cryotherapy experiments.

Plant Species, Cultivar, Virus	Methods	Survival and/or Regeneration [Reference]
*Prunus salicina* Lindley, ‘Methley’ × *Prunus spinom* L., rootstock hybrid ‘Fereley-Jaspi (R)’, PPV, Marcus strain	Vitrification: Pre-culture: 24 h, 4 °C on medium with 5% DMSO, 2% proline, then ST to modified PVS-2 for 20–40 min. Cryotubes frozen: 1 °C/min to −40 °C, then into LN. Next day: rapid warming at 40 °C for 1 min, rinsed with ½ MS with 1.2 M sucrose, and post-cultured.	Regeneration rates: 7 d: 42%; 14 d: 54%; 30 d: 42%. Controls: 69%, 97%, 84%, respectively. [78]
Grapevine, *Vitis vinifera* L., ‘Bruti’, GVA	Encapsulation-dehydration: ST (1 mm) from 4-wk-old culture into ½ MS with 3% Na-alginate, 2 M glycerol, 0.4 M sucrose. Mixture, with ST, into 0.1 M CaCl_2_ with 2 M glycerol, 0.4 M sucrose at RT for 30 min, to form beads (4 mm). Pre-c. of beads: on MS with 0.26% gellan gum, and sucrose content increased daily (0.25, 0.5, 0.75, and 1.0 M) for 4 ds. Then dehydration by air drying, at RT, for 7 h. Then into LN for 1 h. Thawed in a water bath at 40 °C, for 3 min. Post-c: on MS with 3% sucrose, 0.26% gellan gum, 0.05 mM NAA, 3 mM BA. In D at 28 °C for 2 ds., then 24 °C, 16 h L., 45 µE s^−1^ m^−2^.	Survival rate after different steps: encapsulation: 100%; pre-culture: 100%, dehydration: 82%; freezing: 60%. Survival of different ST size: 0.5 mm: 50%; 1.0 mm: 65%; 1.5 mm: 60%; 2.0 mm: 50%. [91]
Grapevine, *Vitis vinifera* L., ‘Bruti’, GVA	Vitrification: Pre-c of ST (1 mm) on ½ MS with sucrose content increased daily: 0.25, 0.5, up to 0.75 M, 0.26% gellan gum, for 3 ds. Then: treatment by mixture of 2 M glycerol, 0.75 M sucrose for 60 min at 25 °C, then dehydration: ½ PVS-2 at 0 °C for 30 min, then full-strength PVS-2. ST into cryotubes, then LN for 1 h. Warmed in water bath at 40 °C for 3 min. Post-c: on ½ MS with 3% sucrose, 0.26% gellan gum, 0.05 mM NAA, 1 mM BA. In D, at 24 °C for 2 days, then 24 °C, 16 h L., 45 µE s^−1^ m^−2^.	Survival rates: control: 100%, encapsulation-dehydration: 62%; vitrification: 50% [91]
Potato, *Solanum tuberosum* L., ‘117’, PLRV, PVY	Encapsulation-dehydration: ST 1–1.5 mm, in MS + 2.5% Na-Alginate, 2 M glycerol, 0.4 M sucrose, into 0.1 M CaCl_2_ (+2 M glycerol, 0.4 M sucrose), 20 min. Pre-c. of beads (4 mm): 0.25; 0.5; and 0.75 M sucrose, increased daily, for 3 d. Surface drying, RT for 0–8 h. Cryotubes into LN for 1 h, then thawing 40 °C, for 3 min. Post-c. MS + 1.0 mg/L GA_3_, 0.4 mg/L BA, D, 22 ± 1 °C, for 3 ds. Then at 22 + 1 °C, 16 h, 50 µE s^−1^ m^−2^.	Water content during dehydration: initial: 67.1%; after 5 h: 20.4%; after 8 h: 15.1%. The best survival: 78% after 5 h dehydration, at least 2 h was necessary. [89]
Potato, *Solanum tuberosum* L., ‘117’, PLRV, PVY	Encapsulation-vitrification: ST 1–1.5 mm, Susp. in MS + 2.5% Na-Alginate, 2 M glycerol, 0.4 M sucrose. Into 0.1 M CaCl_2_ (+2 M glycerol, 0.4 M sucrose), for 20 min. Pre-c. of beads (4 mm): 0.25; 0.5; and 0.75 M sucrose increased daily, for 3 ds. Beads loaded in MS + 0.4 M sucrose, 2 M glycerol, for 90 min., at RT. Vitrification: PVS-2, 0 °C, for 0–240 min. Cryotubes: LN 1 h. Thawed ST washed by 1.0 M sucrose 30 min., RT. Post-c.: MS + 1.0 mg/L GA_3_, 0.4 mg/L BA, D, 22 ± 1 °C, for 3 ds, then at 22 ± 1 °C, 16 h, 50 µE s^−1^ m^−2^.	At least 30 min. vitrification time was necessary for survival, the best survival rate: 75% after 180 min. vitrification. [89]
Potato, *Solanum tuberosum* L., ‘117’, PLRV, PVY	Droplet cryotherapy: Pre-c. of ST (1–1.5 mm): 0.25; 0.5 and 0.75 M sucrose, increased daily, for 3 d. Then ST into cryoprotectant solution: 10% DMSO in MS: 0–160 min. 3.5 µL droplets on aluminium foil; LN 1 h. Thawing: ST washed by 1.0 M sucrose 30 min., RT, Post-culture: MS + 1.0 mg/L GA_3_, 0.4 mg/L BA, kept in D, at 22 ± 1 °C for 3 d, then: 22 + 1 °C, 16 h L, 50 µE s^−1^ m^−2^.	DMSO treatment: at least 20 min. was necessary for survival, the best survival rate: 85% after 120 min. DMSO treatment. [89]
Sweet potato, *Ipomoea batatas* L., line 199004.2, SPFMV, SPCSV	Encapsulation-vitrification: ST (1 mm, 3 LP) from 3-week-old shoots, into 2.5% Na-alginate, 2.0 M glycerol, 0.4 M sucrose in 0.1 M CaCl_2_ solution with 2.0 M glycerol and 0.4 M sucrose. Pre-c. of beads: liquid MS with 0.62 mM calcium nitrate, 1.1 mM AA, 0.04 mM calcium pantotenate, 0.12 mM putrescine dihydrochloride, 0.57 mM l-arginine, 0.24% Gelrite, 0.3 M sucrose on rotary shaker (90 rpm). Then: loading in the same medium with 2.0 M glycerol, 1.6 M sucrose (pH 5.7) (3 h) rotary shaker (60 rpm). Vitrification: PVS-2; RT, for 0 to 180 min. Beads surface-dried, then cryotubes in LN for 1 h, warmed in water bath at 40 °C, 3 min, washing: liquid ammonium-free MS with 1.2 M sucrose (20 min). Post-c: on ammonium-free medium in D, (3 ds), then fresh medium, 22 ± 2 °C, 16 h L, 50 µE s^−1^ m^−2^, for 2 wks.	Survival rates after different duration (min) of PVS-2 treatment: 0: 0%; 30: ~25%; 60: ~42%; 90: ~53%; 120: 85%; 150: ~65%; 180: ~42%. [25]
Sweet potato, *Ipomoea batatas* L., 199004.2 line, SPFMV, SPCSV	Encapsulation dehydration: ST (0.5 mm: 1–2 LP, 1 mm: 3–4 LP, and 1.5 mm: 4 LP) from 3-wk-old shoots used for cryotherapy. Encapsulation: in sodium alginate solution into calcium chloride solution. Freezing in LN for 1 h, then beads in a water bath at 40 °C for 3 min and washing with liquid MS without ammonium, but with 1.2 M sucrose for 20 min. Then surface drying. Post-c: D for 3 ds, on MS without ammonium, then further culture on fresh MS at 22 ± 2 °C, 16 h, 50 µE s^–1^ m^–2^ for 2 wks.	Survival rates of ST in different size: 0.5 mm: 83%; 1.0 mm: 83%; 1.5 mm: 87%. Regeneration rates: 0.5 mm: 18%; 1.0 mm: 87%; 1.5 mm: 87%. [80]
Grapevine, *Vitis vinifera* L., ‘Black’, GVA	Encapsulation-dehydration: ST (1.0 mm), in ¾ MS + 3% Na-alginate, 2 M glycerol, 0.4 M sucrose, 2 µM BA, then ST with medium into 0.1 M CaCl_2_ with 2 M glycerol, 0.4 M sucrose, RT, 30 min: 4 mm beads. Pre-c. of beads on ¾ MS + 0.25; 0.5; 0.75; 1.0 M sucrose (increased daily) for 4 d; dehydrated by air drying at RT for 12 h. Cryotube into LN for 1 h, thawed in 40 °C water bath, for 3 min. Post-culture: ¾ MS with 2 µM BA, in D, at 24 °C, for 2 ds; then at 24 ± 2 °C, 16 h L, 45 µE s^−1^ m^−2^ for 6 wk. Then regeneration of shoot > 3 mm on ¾ MS + 1.5 mg/L or 1.0 mg/L BA.	Survival rates after different steps: control: 100%, after encapsulation: 100%, after dehydration: 100%, after LN: 59%. [92]
Globe artichoke, *Cynara scolymus* L., 12 clones, ALV	Vitrification: ST (1–1.5 mm, 3–4 LP) pre-c: on MS with 0.3 M sucrose, in D at 4 °C for 24 h. ST to cryovials, treated by LS (MS + 2 M glycerol, 0.4 M sucrose) at 25 °C, 30 min. Vitrification: 1 mL PVS-2, at 0 °C, for 55 min. then fresh PVS-2, cryovials into LN for 1 h. Thawing: water bath at 40 °C for 90 s. Washing: MS with 1.2 M sucrose, for 20 min. at 25 °C. ST onto regeneration medium: M1 for early types: MS + 20 g/L sucrose, 0.5 mg/L BA, 0.1 mg/L NAA, 0.5 mg/L GA_3_; M2 for late types: Gik + 0.5 mg/L BA, 0.1 mg/L NAA, 0.05 mg/L GA_3_. Culture at 23 °C, D, for 3 d. Then: 23 ± 1 °C continuous light (4000 lux).	Survival rates: in early types: 70–90%; in late types: ˂25%. Duration of LN treatment (15 or 30 min.): did not affect the regeneration rate. [90]
Apple rootstocks, *Malus domestica* Borkh., ‘M9’, ‘M26’, ASPV, ASGV	Encapsulation-dehydration: Stabilization of ST (0.5 mm (2 LP), 1.0 mm (3–4 LP), 1.5 mm (5–6 LP) on MS with 30 g/L sucrose, 0.25 mg/L BA, 0.01 mg/L IBA, 8 g/L agar, for 1 d. Encapsulated. Pre-c. of beads (5 mm): 0.75 M sucrose (for 7 d), dehydration: air drying (for 6 h) until 21% water content. LN (1 h), then thawing. Post-c: on MS with 30 g/L sucrose, 0.25 mg/L BA, 0.01 mg/L IBA, 8 g/L agar (for 8 wk). Regenerated shoots: to fresh medium, sub-culture: 4 wk.	Regeneration rates: ‘M9’: ST: 0.5 mm: 0%, 1.0 mm: 45.5%, 1.5 mm: 73.8%. ‘M26’: ST: 0.5 mm: 0%, 1.0 mm: 42.5%, 1.5 mm: 75.2% [88]
Grapevine, *Vitis vinifera* L., 9 cultivars GFLV, GLRaV-3	Droplet vitrification: ST from 2-wk-old shoots. Pre-c: on ½ MS with 0.1 M sucrose for 24 h. Then treated with LS (2 M glycerol + 0.4 M sucrose in MS for 20 min at RT). Dehydrated: 1/2 PVS-2 (30 min, RT) then full strength PVS-2 (0 °C, 50 min). Buds placed in 5 µL PVS-2 droplets on Al foils, then in LN (1 h). Rewarming: Al foils immersed in unloading solution (1.2 M sucrose) (20 min, RT). Post-c: medium with 1µM BA, D (26 ± 1 °C) for 7 d; then at 27 ± 2 °C, 12 h, 40 μE∙m^−2^∙s^−1^.	Survival/regeneration rates: ‘Portan’: 50%/50%; ‘Chardonnay’: 51–61%/30–31%; ‘Cabernet Sauvignon’: 58–62%/42–47%; ‘Merlot’: 75–68%/61–70%; ‘Pinot Noir’: 38–48%/0%; ‘Plavac mali’: 0%/0%; ‘Maraština’: 22–25%/11%; ‘Pošip’: 0%/0%; ‘Škrlet’: 15%/0%. [93]
Grapevine, *Vitis vinifera* L., ‘Cabernet Sauvignon’, ‘Chardonnay’, *V. vinifera* × *V*. *labrusca*, ‘Kyoho’, *V*. *pseudoreticulata*, ‘Hunan-1’ (rootstock), GLRaV-3	Droplet vitrification: Pre-c. of ST (1.0 mm, 5–6 LP) on medium with 0.3 mM sucrose, 0.16 mM glutathione, 0.14 µM AA, for 3 d. Loaded in 2 M glycerol, 0.4 M sucrose, for 20 min, at RT. Vitrification: 0 °C, ½ PVS-2, 30 min., then full PVS-2 for 50 min. ST into 2.5 µl PVS-2 droplet, into LN for 1 h. Rewarm: into unloading solution, MS + 1.2 M sucrose, RT, 20 min. Post-c: on ½ MS + 0.6 M sucrose, 7 g/L agar, D for 1 d, then onto ½ MS + 30 g/L sucrose, 7 g/L agar + 0.5 mg/L BA. In ‘Cabernet Sauvignon’ the ST sizes: (0.5 mm (3–4 LP), 1.0 mm (5–6 LP), 1.5 mm (6–7 LP)), and exposition time of full strength PVS-2 (50, 75, 100 min) were tested.	Survival rates after 7 ds/regeneration rates after 8 wk: PVS-2: for 50 min: 75%/58%; for 75 min: 50%/20%; for 100 min: 23%/11%. ST size: 0.5 mm: 52%/23%; 1.0 mm: 75%/59%; 1.5 mm: 60%/48%. Three cultivars 43–51% regrowth and 95% survival during acclimatization. [24]
Apple rootstock, *Malus prunifolia* (Wild) Borkh., ‘Marubakaido’, ACLSV, ASPV, ASGV	Encapsulation-dehydration: Ax. ST (1.5 mm, 3–4 LP) from 4-wk-old shoots. Pre-c: on MS with 30 g/L sucrose, 0.25 mg/L BA, 0.01 mg/L IBA, 2.6 g/L Phytagel™, in D for 1 d. Then ST in liquid MS (no calcium), with 2.5% Na-alginate, 2 M glycerol, 0.4 M sucrose. Dropped into 0.1 M CaCl_2_ with 2 M glycerol, 0.4 M sucrose in liquid MS for 20 min. at 25 ± 2 °C. Pre-c. of beads: in D for 7 ds in MS with 0.5 M sucrose, 2.6 g/L Phytagel™, 25 ± 2 °C. dehydration: by air flow for 0, 4, 5, 6, 7, 8, and 9 h, at 25 ± 2 °C and 29% RH. ST into cryotubes in LN for 1 h. Then warming: 40 °C water bath (for 3 min). Post-c: on MS with 40 g/L sucrose, 1 mg/L BA, 2.6 g/L Phytagel™ for 24 h, then onto fresh medium, in D for 7 ds, at 25 ± 2 °C, then ST removed from beads, to fresh medium, culture: 25 ± 2 °C, 16 h L, 50 μmol m^−2^ s^−1^. Sub-culture 5–6 wks.	Water content: initial: 79%, after different duration of drying: 4 h: 34%; 7 h: 23%; 9 h: 19%. The best survival and regeneration rates: 53% and 35% after 7 h dehydration. [94]
Potato, *Solanum tuberosum* L., ‘981818’, ‘T01-7-70’ clones, PVS	Droplet-cryotherapy: Pre-c. of shoots: on MS with SA: 0 M, 10^−5^ M, or 10^−6^ M for 28 d. Pre-c: of isolated Ax buds (1–2 mm) on SA-free MS with 0.3 M sucrose at 21 °C for 3 ds. Droplets of Na-alginate solution (2%) with 0.4 M sucrose in MS added to cryoplate well. Buds into well, covered with Na-alginate solution, BEMCOT paper, calcium chloride solution (0.1 M calcium chloride, 0.4 M sucrose in MS), until covered completely. Polymerization for 15 min at RT. Excess calcium chloride solution removed. Cryoplates into LS (2.0 M glycerol, 1.0 M sucrose in MS) for 45 min. Then cryoplates dehydrated by 35 g silica gel, for 90 min, at 24 °C, into cryotubes, held on a cryocane, filled with LN, for 1 h. Cryotubes rewarmed in 1 M sucrose solution with MS for 15 min. at RT. Buds removed from cryoplates, onto solid MS, then buds removed from the alginate gel and onto fresh solid MS.	Survival rates: without AS treatment: 0%, treatment with 10^−6^ M SA: ‘T01-7-70’: 70%, ‘981818’: 28.3%. 10^−5^ M SA: ‘T01-7-70’: 0%, in ‘981818’: 10%. [95]
Apple, *Malus domestica* Borkh., ‘SC417 Monalisa’ (Gala × Malus 4), ACLSV, ASGV, ASPV	Droplet vitrification: Stabilization of Ax ST (1 mm, 2-3LP) from 4-week-old shoots, on MS with 30 g/L sucrose, 0.25 mg/L BA, 0.01 mg/L IBA, 2.6 g/L Phytagel™, for 1 d, at 25 ± 2 °C, in D. Pre-c. of ST on MS with 2 M glycerol, 0.8 M sucrose for 1 d., at 25 ± 2 °C, in D. Then placed in PVS-2, RT or 0 °C for 0, 20, 40, 50, 60 or 80 min. Then ST into 2.5 μL PVS-2 droplets on Al foil strips, and into LN for few minutes, then into cryotubes filled with LN for 1 h. Warming: into unloading solution (MS + 1.2 M sucrose at pH 5.8) RT, for 20 min. Regeneration: on MS with 30 g/L sucrose, 0.25 mg/L BA, 0.01 mg/L IBA, 2.6 g/L Phytagel™, at pH 5.8, for overnight in D, then onto fresh medium. Kept for 7 d in D, 25 ± 2 °C, then 25 ± 2 °C, 16 h L., 50 μmol m^−2^ s ^−1^. Rooting: MS with 30 g/L sucrose, 1 mg/L IAA, 2.6 g/L Phytagel™.	Survival/regeneration rates after different duration of vitrification: At RT: 0 min: 0%/0%; 20 min: 70%/45%; 40 min: 65%/45%; 50 min: 40%/<40%; 60 min: 25%/<20%; 80 min: 10%/0%. At 0 °C: 0 min: 0%/0%; 20 min: <65%/<40%; 40 min: 78%/58%; 50 min: 77%/51%; 60 min: <60%/<40%; 80 min: <45%/<20%. [87]

µE: microEinsteins; AA: ascorbic acid; ACLSV: Apple chlorotic leaf spot virus; Al: aluminium, ALV: Artichoke latent virus; ASGV: Apple stem grooving virus; ASPV: Apple stem pitting virus; Ax: axillary; BA: 6-benzyladenine; d(s): day, days; D: darkness; DMSO: dimethyl sulfoxide; GA_3_: gibberellic acid; GFLV: grapevine fanleaf virus; Gik: Gik medium [96]; GLRaV-3: grapevine leafroll-associated virus-3; GVA: grapevine virus A; h(s): hour(s); IAA: indoleacetic acid; IBA: Indole-3-butyric acid; L: light; LN: liquid nitrogen, LP: leaf primordium; LS: loading solution; min: minute; MS: MS medium [62]; NAA: α-naphthylacetic acid; PLRV: Potato leafroll virus; Post-c.: post-culture; PPV: Plum Pox Potyvirus; Pre-c: pre-culture; PVS: Potato virus S; PVS-2 solution: contains 30% glycerol, 15% ethylene glycol, 15% DMSO and 0.4 M sucrose in MS medium (pH 5.8) [26]; PVY: Potato virus Y; RT: room temperature; SA: salicylic acid; SPCSV: Sweet potato chlorotic stunt virus; SPFMV: Sweet potato feathery mottle virus; ST: shoot tip; Wk(s): week(s).

### 3.2. The Role of Genotypes and Explant in the Survival and Regeneration Rate

The freezing tolerance of 12 globe artichoke (*Cynara scolymus* L.) clones belonging to different groups of maturity were tested [90]. Even though the survival rates were high (80–90%) and more or less uniform, the genotypes belonging to early maturity group tolerated the cryo-treatment better and showed higher regeneration rates (70–90%) than late genotypes (˂25%) [90] (Table 2). The majority of tested Norwegian potato (*Solanum tuberosum* L.) cultivars (‘Truls’, ‘Gammelraude’, ‘Abundance’, ‘Gjernespotet’, ‘Iverpotet/Smaragd’, and ‘Sverre’) showed regeneration rates between 40–60%, but the best results (70%) were found in ‘Hroar Dege’, and the lowest rate (30%) in ‘Kerrs Pink blått skall’ [59]. Both healthy and infected plants (Grapevine fanleaf virus, GFLV; Grapevine leafroll-associated virus type 1 and 3, GLRaV-1 and GLRaV-3) in several grapevine (*Vitis vinifera* L.) genotypes including Croatian cultivars were tested in cryotherapy experiments. Survivors were found only seven of the nine varieties, and only five cultivars were able to regenerate shoots [93]. Croatian genotypes were proven to be more sensitive to the cryotherapy (15–25% survival rates) compared to other cultivars (37–75% survival) [93]. From survivors, the ‘Pinot Noir’ did not regenerate the shoot at all, and only ‘Maraština’ was able to regrow from Croatian cultivars at a low rate (11%). The best results were obtained in ‘Merlot’ (85.5–90% survival, 61–70% regeneration). The healthy status of donor plants did not affect the survival and regrowth results. Marković et al. [93] supposed that the weak stress tolerance ability of Croatian cultivars maybe was due to their lack of adaptation to in vitro conditions, because their in vitro cultures were established just before the experiment.

Duration of the last sub-culture (the age of donor plantlets) influenced the regeneration rate of both the treated and control shoot tips: in the case of *Prunus* rootstock (‘Fereley–Jaspi (R)’), the best results were obtained after a 14-day-long sub-culture period [78]. Helliot et al. [74] used highly proliferating meristematic clumps from banana (*Musa* L. spp., ‘Williams’) in vitro culture for cryotherapy, because this explant type showed a better regeneration rate than the single meristem. This different response was maybe due to the fact that the apical dome (AD) of highly proliferating meristem is not covered by leaf primordium (LP), while the apical dome of the single meristem is coated by LP, which may prevent the entry of cryoprotective solutions; thus, the dehydration of this region cannot happen. Additionally, it was hypothesized that freezing occurs too slowly due to the insulator effect of air between the AD and the covering LP.

### 3.3. The Effect of Shoot Tips Size

No difference in survival rates (83–87%) was found for shoot tips of different sizes in sweet potato (*I. batatas* (L.) Lam) (199004.2 line); however, the regeneration capacity of the smallest explants tested (0.5 mm) was very poor (18%), while 87% of the larger shoot tips (1.0–1.5 mm) regenerated shoots [25]. Li et al. [88] also found that as the shoot tip size increased (from 0.5 to 1.5 mm), the regenerative capacity increased from 0% to over 70% in the case of apple rootstocks (*M. domestica* Borkh) ‘M9’ and ‘M26’. Explants larger than 0.5 mm regenerated significantly better than smaller ones in treated *Prunus* rootstock (‘Fereley-Jaspi (R)’) culture [78]. The highest survival and regrowth rates (75% and 59%, respectively) were found in grapevine (*V. vinifera* L., ‘Cabernet Sauvignon’) when a 1.0-mm-long shoot tip was used as explant in experiments where shoot tips in the length between 0.5 and 1.5 mm were tested [24]. In the study of Wang et al. [91], 1.0 and 1.5 mm shoot tip sizes resulted in 65% and 60% regrowth of cryo-treated shoots in grapevine (*V. vinifera* L., ‘Bruti’).

### 3.4. The Effect of Pre-Treatments

The survival rates of grapevine (*V. vinifera* L., ‘Black’) shoot tips (1.0 mm) after the different steps of cryotherapy were 100% in control (without any treatment), as well as after encapsulation and dehydration, while only 59% of explants survived the freezing in liquid nitrogen [92]. Before and during cryotherapy, several methods have been used to increase the survival of treated shoot tips. Pre-treatment of plants or explants before cooling to ultra-low temperatures improves the chances of tissue survival. Pre-treatments include (1) pre-treatment of cultures (medium, acclimation to low temperatures, selection of appropriate plant parts), or (2) chemical treatments (osmotic treatments or application of cryoprotectants penetrating into the tissues), and (3) dehydration of plant parts [84].

#### 3.4.1. Pre-Treatments of Mother Cultures

A short-day photoperiod combined with cold treatment imitating the winter period and lasting for some weeks can be used mainly for temperate zone species [84], because they have a mechanism that enables them to have better freezing tolerance after adequate cold acclimatization [97]. In several plant species, various substances (low molecular weight, cryoprotective, compatible solutes) accumulate during cold acclimation (di- and polysaccharides, polyol, sorbitol, proline, polyamines, etc.) [85]. These molecules can help to maintain the original form of protein conformation and can enhance suitable membranes structure as well [85]. The sucrose concentration in plant can increase up to tenfold during cold treatment, and starch can be converted to sucrose. The lipid content and the structure of membranes and the ratio of unsaturated/saturated fatty acid also can change, and all these alterations play role in preventing freezing damages [84,85]. An alternate treatment was used for cold acclimation of apple (*Malus sieversii* Ledeb. M. Roem. and *M. domestica* Borkh.) shoots: 8 h illumination at 22 °C (10 µE m^−2^ s^−1^) and a 16 h dark period at 1 °C alternated before cryotherapy [98].

Potato clones (*S. tuberosum* L., T01-7-70 and 981818) were proven to be very sensitive to freezing and did not survive cryotherapy at all. They were incubated on a medium supplemented with salicylic acid (SA) prior to cryotherapy in order to enhance their survival. Treatment with 10^−6^ M SA resulted in 70% and 28.3% survival rates in T01-7-70 and 981818 clones, respectively, while the addition of 10^−5^ M SA to the medium was less favorable and led to the death of each explant of the T01-7-70 clone and only a low (10%) survival rate in explants of the 981818 clone [95].

#### 3.4.2. Pre-Treatments of Explants by Vitrification Solutions and Osmoprotectants

Pre-cultures of the explants for 24 h immediately after isolation on MS medium containing 100 mg/L myo-inositol, 30 g/L sucrose, 0.5 mg/L BA, 0.05 mg/L IBA, 3.5 g/L agar, and 1.2 g/L gelrite can reduce the effect of excisional stress; thus, they can stabilize the condition of the explants [81].

In order to reduce freezing injuries, the removal of intracellular water is very important, and high concentration plant vitrification solutions (PVS) have been developed for this purpose [99,100]. Cryoprotective solutions dehydrate plant tissues, so water molecules do not assemble into ice crystals during rapid freezing, but become a “vitreous” state, a process called vitrification, which means that the solidification of the liquid is caused by an extreme increase in viscosity, and the solution becomes a “glass” during cooling [101]. In virus elimination processes, the PVS-2 (which originally contains 30% (*w*/*v*) glycerol, 15% (*w*/*v*) ethylene glycol, 15% (*w*/*v*) dimethyl sulfoxide (DMSO), and 0.15 M sucrose in MT [102] medium) is very commonly used for shoot tips before freezing [99]. Modified version of PVS-2 (0.4 M sucrose, in MS) is the most frequently used [103]. Bettoni et al. [87] found that PVS-2 treatment was the prerequisite for survival of apple (*M. domestica* Borkh., ‘Monalisa’) cryo-treated shoot tips; without PVS-2 treatment, no survivor was found at all. Similarly, the shoot tips of sweet potato (*I. batatas* (L) Lam, 199004.2 line) also did not survive the cryotherapy without PVS-2 treatment [25].

Duration of PVS treatment and the temperature of vitrification have significant effect on the survival and regeneration rates of treated explants [81]. However, the length of treatment required depends on the species or genotype to be treated. Bi et al. [24] studied the effect of the duration (50, 75, and 100 min) of full strength PVS-2 treatment on the survival and regrowth of grapevine (*V. vinifera* L., ‘Cabernet Sauvignon’) shoot tips (1.0 mm). As the duration of treatment increased from 50 to 100 min, both the survival and regeneration rates decreased from 75% to 23% and 58% to 11%, respectively. At least 30 min of treatment was necessary to get a survivor at all [89]. Treatment with PVS-2 for 180 min resulted in the best survival rate (75%) in potato (*S. tuberosum* L.) shoot tips; but a longer period significantly decreased the survival. The best survival rate (85%) for sweet potato (*I. batatas* (L.) Lam, 199004.2 line) was obtained by a 120-min-long PVS-2 exposure [25]. Treatment of apple (*M. domestica* Borkh., ‘Monalisa’) shoot tips at room temperature for more than 80 min was fatal: each shoot tip died [87], and the optimal durations were 20- and 40 min-long-treatment, which resulted in 70% and 65% survival rates, respectively, followed by 45% regrowth in both treatments. However, the best survival and regeneration rates were observed when shoot tips were treated by PVS-2 at 0 °C for 40 and 50 min (78–77% survival, 58–51% regeneration rates). The authors found that explants treated by PVS-2 at the lower (0 °C) temperature tolerated the longer treatment better [87].

The osmo-protectant chemicals are proven to be toxic to living cells depending on the concentration used, as reviewed by Best [104]; some of them, such as DMSO and glycerol used in cryotherapy of plant tissues, can also be phytotoxic [105,106,107,108]. Although at least 20-min-long treatment by DMSO was necessary to get survivor potato (*S. tuberosum* L.) shoot tips, and the best survival rate (85%) was achieved by 120-min-long treatment, longer period reduced the survival rates [89]. Moreover, there are plant species, such as avocado (*Persea americana* Mill.), which hardly tolerate the osmotic stress created by DMSO; thus, they need pre-conditioning to enhance their survival. Applying high sucrose level (0.3 M) or cold treatment (10 °C for 2 weeks) on donor plants of avocado (*P. americana* Mill., ‘Velvick’, ‘Reed’) was found to be able to decrease the damages caused by PVS-2 [109]. A relationship was found between the abiotic stress tolerance and response to pre-condition of cultivars. The ‘Velvick’ cultivar, which is sensible to cold but has a good salt tolerance, responded well to high sucrose treatment (83% survival and 73% regrowth compared to the control: 70% and 23%, respectively), but did not prefer the cold treatment. The other genotype (‘Reed’), which has a good cold tolerance and moderate salt tolerance, preferred the pre-conditioning by cold treatment (86% survival and 80% regrowth compared to control 23% and 16%, respectively). In the latter cultivar, pre-conditioning by high sucrose resulted in 76% survival and 40% regeneration.

### 3.5. Dehydration by Physical Drying

The water content can be also decreased by physical drying, with application of air-flow or silica gel. In general, the best water content of shoot tips before cryo-treatment is about 20% [81].

During air-drying, the water content of the apple (*M. prunifolia* (Wild.) Borkh., ‘Marubakaido’) shoot tips decreased sharply in the first 4 h from the initial 79% to 34%, then the rate of decline moderated. The best survival (more than 50%) and regeneration percentages (35%) were achieved after a 7-h-long drying, when 23% water content could be measured [94]. Moreover, the normal development of regenerated shoots of ‘Marubakaido’ (*M. prunifolia* (Wild.) Borkh.) required air-drying, which lasted for 6–9 h. If treatment was shorter than 6 h, only green calli or hyperhydrated leaf tissues developed [94]. Dehydration by air flow of at least for 2 h was required for survival of potato (*S. tuberosum* L.) shoot tips resulted in the loss of about half of the water content. However, the best survival rate (78%) was obtained when shoot tips were dehydrated for 5 h in potato (*S. tuberosum* L.); during this period, the initial water content had dropped from 67.1% to 20.4%. Dehydration for more than 5 h significantly reduced survival [89].

### 3.6. Suggestions for Improvement of Survival and Regeneration Ability of Explants

Although there is plenty of information on cryopreservation treatment of different plant species and cultivars, the different steps of cryotherapy methods should always be optimized for the genotype to be treated, because genotype-dependence in treatment responses is very strong. The ability of shoots to regenerate can be improved by bringing the donor plant to a proper physiological state (age of mother plant and cold acclimation of temperate zone species). If possible, the use of an explant from a tissue culture that has already been adapted to the in vitro culture conditions is recommended. A stabilization period of 24 h on specific medium after excision can also enhance the stress tolerance of explants. Several methods of pre-treatments can also be used after optimization of conditions (duration of treatment, ambient temperature, concentration and content of vitrification solutions and osmoprotectants, the means of dehydration by physical drying).

## 4. Virus Elimination by Thermotherapy

### 4.1. The Background

All the processes of multiplication, inactivation, and moving of viruses in plants are dependent on the temperature. Although multiplication of viruses can increase at more than 22 °C, the progeny viruses can be inactivated quickly at 30 °C or higher temperature [110]. Exposure of tissue cultures to heat treatment by application of temperature higher than 28 °C can reduce significantly the virus content in plants. In general, at 28 °C, the infectivity of viruses already has been reduced, but application of at least 32 °C is required for effective virus eradication [111]. Raising the temperature to 40 °C almost promptly stopped the single-stranded progeny RNA synthesis of Tobacco mosaic virus (TMV) [112], as well as the RNA synthesis in host tobacco (*Nicotiana tabacum* L., ‘Xanthi’) plants [113]. Several thermotherapy experiments applying 40 °C were successful, e.g., each tobacco (*Nicotiana rustica* L.) in vitro culture was free from Cherry leaf roll virus (CLRV) [111]. Virus-free plants were obtained at a high rate when cowpea (*Vigna unguiculate* (L.) Walp) plants infected by Cowpea chlorotic mottle virus (CCMV) were treated by alternating temperature regimes, including an increase of temperatures up to 45 °C [114]. Even though Wang et al. [26] did not obtained plantlets fully free from Raspberry bushy dwarf virus (RBDV), by heat treatment applied on raspberries (*Rubus idaeus* L., ‘Z13’), they could detect that the viral RNA and coat protein (CP) content in the shoot tips were significantly reduced by high temperature (38 °C). The amount of viral RNA2 in shoot tips and leaves rapidly decreased after heat treatment for five days, similarly to viral RNA3, which could be detected only in its remains after thermotherapy for eight days [26]. Degradation of CP also plays an import role in virus eradication, because viral CP is involved in the CP-dependent cell-to cell transport of viruses. CP also takes part in translation of viral RNA by direction the viral genome to the proper site for replication [115]. Immunolocalization studies revealed that 20–27% of the 0.5 mm meristematic region of shallot (*Allium cepa* var. *aggregatum*, 10603) was free from Onion yellow dwarf virus (OYDV) and Shallot latent virus (LSV), without thermotherapy, respectively. After heat treatment, 66% and 73% of the 0.5 mm meristematic region became free of those viruses, respectively [116]. Effectiveness of thermotherapy can be affected by virus type and the host genotype. Apple stem grooving virus (ASGV) was not detectable in the AD region and LP1 after a two-week-long heat treatment by 36/32 °C temperature applied on ‘Gala’ (apple, *Malus domestica* Borkh.) in vitro shoots infected by ASGV, but the virus was still present in LP3 and older tissues. However, after a four-week-long treatment, the virus was only detectable in LP6 or older regions. Prior to thermotherapy, virus distribution was similar in ‘Ruixue’ and ‘Gala’ cultivars; however, in ‘Ruixue’, the virus was still detectable in LP5 and older regions after four weeks of treatment, and even occurred in LP4 with 80% frequency [38].

Thermotherapy can be applied on both in vivo and on in vitro plants; in the former case, the treatment is followed by in vitro culture from shoot tips or meristems. In general, meristems or shoot tips have been isolated and cultured, or treated tissues have been sub-cultured after thermotherapy (see Table 3). The temperature range of 32–40 °C has been used in previous experiments, but about 37–38 °C is the most frequently applied. However, in the case of tropical plants, e.g., sugarcane (*Saccharum officinarum* L.), the temperature of treatment can be increased even up to 50 °C [117].

Thermotherapy can occur by application of a constant or dual regime of temperature. For example, grapevine (*Vitis vinifera* L.) in vitro cultures were treated by a constant 39 °C (for 32 days) in the case of ‘Plavac Mali’ [118], or were exposed to a dual regime of temperature (37.5/34 °C during 16 h light/8 h dark period) in the case of ‘Napoleon’ clones [119]. Similarly, for virus elimination of sweet potato (*Ipomoea batatas* L.), also dual regimes of temperature (32/28 °C; 36/32 °C; 40/34 °C) could be used effectively [120]. The thermotherapy can be repeated by a second cycle to increase the amount of virus-free plant material as it was reported for the potato (*Solanum tuberosum* L.) sanitation by some researchers. The first time, plantlets were exposed to the 37 °C (for 40 days), then the plantlets that remained infected were multiplied by sub-cultures and again exposed to the treatment with the same condition [121,122]. A very specific method was suggested for thermotherapy of artichoke (*Cynara cardunculus* L. var. *scolymus*) in order to get usable explants for post-culture. The treatment included the gradual raising and lowering of the temperature (28 °C: 1st and 2nd day, 30 °C on 3rd day, 38 °C from 4th to 14th days, 36 °C from 15th to 18th days, 34 °C from 19th to 21st days, 32 °C from 22nd to 23rd days, 30 °C from 24th to 25th days, 28 °C from 26th to 28th days, and finally 26 °C on the last (29th) day [123]).

Thermotherapy is often combined with other virus elimination treatments, such as chemotherapy or cryotherapy. The combined methods including thermotherapy have been summarized by Wang et al. [124]. The great variety of thermotherapy methods all serve the goal of obtaining the highest possible proportion of surviving plants with good regeneration ability, without decreasing the antiviral efficacy. To tell the truth, plants treated with thermotherapy are exposed to quite a lot of stress. The higher plants respond with special cellular and metabolic changes when exposed to a temperature effect that is in excess by at least 5 °C of their optimal temperature range. These changes are designed to ensure the survival of the plants [125]. In a natural environment, the onset of heat stress symptoms depends on the plant species and its natural habitat. A temperate zone plant may respond to a short 30-min exposure at 40–45 °C with leaf damage, while desert plants with many adaptation mechanisms may withstand the much higher temperatures and possibly longer periods without signs of damage [126].

Heat stress induced symptoms of damages such as yellowing, browning, wilting or necrotic leaves appeared on grapevine (*Vitis champinii* Planch.) cultures shortly after the beginning of treatment at 37 °C [127]. A similar phenomenon (chlorosis, wilting, necrosis, etc.) was observed on sweet potato (*Ipomoea batatas* (L.) Lam) plantlets treated by diurnal temperature regimes [120]. Yellow discoloration was mainly the characteristic of the basal part of the shoots in heat treated (36/32 °C) apple (*Malus domestica* Borkh.) cultivars [38]. Growth of cassava (*Manihot esculenta* Crantz, Tanzanian landscape cultivar) plantlets at 30 °C was normal, similarly to controls (grown at 28 °C), while those treated by a higher temperature (35 and 40 °C) showed abnormal development, and hyperhydration occurred. The leaves often became chlorotic, dried, and finally fell off [128]. However, thermotherapy can result in other morphological and developmental changes, which are not necessarily harmful. The length of in vitro apple (*Malus domestica* Borkh) shoots was reduced by high temperature (36/32 °C) from 3.5 cm observed in control plants to 2.5 cm. Since the multiplication rate was significantly increased by heat treatment (from 2.5 to 4.7 shoots/explant obtained in control and in treated, respectively) [38], development of the shorter shoots maybe related to the higher proliferation rate [43]. In contrast, Hu et al. [127] found significantly longer shoots in heat-treated (37 °C for 20 days) cultures of grapevine (*Vitis champinii* Planch.).

Histological studies revealed changes in tissues induced by high temperature. The heat-treated plants of raspberries (*Rubus idaeus* L., Z13) suffered from heat stress, and tissues showed symptoms and damage of heat stress: cell staining was weaker than of untreated plants, and the histological structure of the tissues was looser in the leaf primordia and in the base of meristem. Sub-cellular alterations were also detected, while in the apical dome region of the meristem, densely stained nucleoli were observed; few of these were detected in the first and second leaf primordia. The cells and vacuoles in the more differentiated tissues were enlarged due to the thermotherapy [26]. Moreover, decreased nucleo/cytoplasma ratio induced by heat treatment was also detected in shallot (*Allium cepa* var. *aggregatum* G. Don, 10603) [116].

Changes can be also detected at the molecular level. As a result of many stresses including heat stress, the plant suffers oxidative damage, during which the amount of ROS increases in the plant, cell metabolism may change, membrane damage may occur, the oxygen generation system may be inactivated, and so on. Finally, all of these changes can even lead to cell death [129]. The increased level of superoxide (O^2−^) in heat stressed plants has been reported for several species as summarized by Hasanuzzaman et al. [129]. ROS molecules are toxic at high concentrations, but at lower concentrations they serve as signaling molecules that increase stress tolerance to a number of stress effects. SA molecule belonging to the phenolic compounds with the hydroxyl group may also play an important role in the development of heat tolerance [130]. Knowledge of the changes induced by heat stress can help to develop methods that can reduce the harmful effects of high temperatures and/or increase the heat tolerance of treated plants or cultures.

### 4.2. Sensitivity of Species, and Effect of Genotypes and Explant Types

The application of heat treatment, e.g., in the case of tulips (*Tulipa* sp. L.) and narcissus (*Narcissus* sp. L.), is almost completely impossible; in vitro tulip shoots are very sensitive to temperatures above 20 °C, a dormancy state develops, and the regeneration and development of new shoots is fully inhibited [131]. Potato (*Solanum tuberosum* L.) varieties showed a different tolerance to the long-term (60–65 days) thermotherapy (27 ± 1, 30 ± 1, and 35 ± 1 °C) applied immediately after meristem isolation. Although the differences in the average of the treatments were not very large (19.1%, 22.0% and 26.0% survival rates were found in ‘Diamant’, ‘Heera’, and ‘Lalpakri’, respectively), they were still significantly different [132]. Potato (*S. tubeosum* L., ‘Baraka’) plantlets treated by 37 ± 2 °C for 40 days under continuous light survived in a rate of 61.5% [122]. Different sensitivity of potato cultivars was also reported by Waswa et al. [133]. In their experiments ‘Kinigi’, ‘Rwangume’, and ‘Victoria’ cultivars regenerated in 54.3%, 65.3%, and 39.3%, respectively, in the average of the whole experiment. However, the survival rate of potato (*S. tubeosum* L.) varieties depended also on the virus infection of them: PVS infected plants of ‘Rwangume’ and ‘Victoria’ survived the thermotherapy to a greater extent (75.4% and 67.1%) compared to PVX infected plants (61.7% and 67.1%). Responses of ‘Kinigi’ variety were contrary: the survival rate of PVS infected plants was 54.6%, while PVX infected ones survived at a rate of 64.6% [133]. Artichoke (*Cynara cardunculus* L. var. *scolymus*) plants infected by Artichoke Italian latent virus (AILV) or Artichoke latent virus (ArLV) responded also very differently to heat treatment considering their survival rates, which were 90.9% and 6.5%, respectively. Authors supposed that the reason for this large difference may be the decreased stress tolerance of plants infected by ArLV, due to their very weak condition [123]. Although stone fruit species are considered to be heat sensitive [134], the ‘Early Rivers’ sweet cherry (*Cerasus avium* (L.) Moench) variety showed very good tolerance to heat stress, since all shoots survived the treatment by 36 °C for four weeks. The majority of the myrobalan (*Prunus cerasifera* var. *divaricata* Borgh) shoots (80%) also survived the treatment, while in vitro shoots of ‘Empress’ plum (*Prunus domestica* L.) cultivar survived the thermotherapy in 66.7% [135]. All shoots of different apple (*M. domestica* Borkh.) cultivars survived the heat treatment by 36/32 °C [38]. Similarly, after treatment of sand pear (*Pyrus pyrifolia* Burm, ‘Jinshui no. 2’) in vitro shoots by 35 ± 0.5 °C for 40 days, 100% survival rate was achieved by excised shoot tips [4]. The survival of plants during thermotherapy alone is not sufficient for effective virus eradication. The regeneration capacity of explants sub-cultured after thermotherapy is as important as survival in order to obtain virus-free plant material. In general, regeneration rates are lower than survival rates. The meristems of onion plants survived 100%, and only 55% regenerated in the control treatment (without thermotherapy), while meristems from heat treated (at 36 °C) plantlets for four weeks survived and regenerated at a rate of 62% and 32%, respectively [116].

To increase the survival and regeneration rates, an alternate regime of temperature has been used instead of constant high temperature in virus elimination process for several species, which can decrease the harmful stress effect of heat treatment [114]. During alternating thermotherapy, the high temperature periods alternate with lower temperature periods. The method can be used to eliminate viruses from plants, because plants recover faster after heat shock than viruses, as was observed in tobacco (*Nicotiana tabacum* L.). When plants were back to optimal temperature, their RNA synthesis (previously stopped by 40 °C) was immediately restored [114], while a delayed recovery was observed for RNA synthesis in Tobacco mosaic virus [136]. Therefore, exposure to high temperature interrupted for sufficiently short periods of lower temperature is gentle on the plants, but does not reduce the effectiveness of the virus eradication. For example, alternate temperature regimes were used in experiments with sweet potato (*Ipomoea batatas* L.) (see Table 3), and significant differences were found in the regeneration rates of sweet potato (*I. batatas* L.) cultivars, so ‘Tanzania’, ‘New Kawogo’, ‘Busia’, and the clone 199004.2 regenerated shoots at rates of 77%, 78%, 70%, and 82%, respectively [120]. Regeneration rates of grapevine clones (*Vitis vinifera* L., Napoleon clones see Table 3) were also significantly reduced after thermotherapy (37.5/34 °C for 1.5 months) from 75.3% (control) to 58.8% (treated) in the average of clones. However, in the average of clones, the regeneration rate of terminal buds (65.3%) was much lower than those of the second and third buds (both 80%) isolated from treated cultures [119].

The age of the in vitro culture involved in the thermotherapy also can influence the outcome of the treatments. After heat treatment of peach (*Prunus persica* (L.) Batsch) in vitro shoots, the best survival rates were obtained when treatment started on 15- and 18-day-old plantlets of ‘Summerset’ and ‘Hermosa’ cultivars, respectively [137].

### 4.3. The Effect of Medium Composition

When thermotherapy (27 ± 1, 30 ± 1, and 35 ± 1 °C) was applied immediately after meristem isolation on media with different combinations of PGRs (BA and GA_3_ in different concentrations), no survivor was found on the PGR-free medium and on media without BA. The lowest survival rates were found on media without GA_3_ but containing BA, while the most survivors (39.7%, 46.1%, and 42.4%) were observed on media containing 0.2 mg/L GA_3_ at all BA levels (1.5, 3.0, and 4.5 mg/L, respectively) [132]. Presence of BA (2.0 mg/L) in the medium for shoot tip culture significantly decreased the regeneration rates of grapevine (*Vitis vinifera* L, ‘Manto Negro’ clone: MPL15.01) from 35.5% obtained on PGR-free medium to 13% (in average of both heat treatment, see Table 3) [138]. Moreover, shoot tips cultured on medium with 2.0 mg/L BA showed high rates of proliferation and hyperhydricity [138]. During the antiviral treatment of peaches (*Prunus persica* (L.) Batsch) with thermotherapy, it has also been found that optimizing the BA concentration added to the medium can improve the survival of the shoots. The effect of BA concentration in the range of 0.01 to 6 mg/L was tested during 35 days of treatment at 35 °C. According to their results, the shoots died within five days on the medium containing 6 mg/L BA. However, reducing the BA level to 0.2 mg/L resulted in more than 90% survival rate for both tested varieties (‘Hermosa’ and ‘Summerset’) [137].

To improve the stress tolerance of potato (*S. tuberosum* L.) plants SA and hydrogen peroxide (H_2_O_2_) were applied before heat treatment of the plants. SA was added to the medium (10^−5^ or 10^−6^ M) and the potatoes were grown on it for 30 days before thermotherapy (32/42 °C 23/1 h for 35 days). Heat treatment was applied either immediately or after a 30-day sub-culture (short- and long-term effects). A similar experiment was performed to test the effect of H_2_O_2_ (plants soaked in 1.0 or 0.5 mM H_2_O_2_ solutions for 1 h); then, they were rinsed and heat treated after the first or second sub-culture (30 days). All treatments significantly increased the survival; however, there was no significant improvement in the regenerative capacity [130].

**Table 3:** Details of the thermotherapy experiments studied in this review.

Plant Species, Cultivar, Virus	Methods	Survival and/or Regeneration [Reference]
Peach (*Prunus persica* (L.) Batsch), ‘Hermosa’, ‘Summerset’, PNRSV	Thermotherapy: on AP medium + Gentamycin (40 mg/L), BA 0.2 mg/L. Shoot age: ‘Hermosa’ 18 d, ‘Summerset’ 15 d. Heat treatment: T1: 38/28 °C (16h L/8h D); T2: 28/39 °C (16 h L/8 h D); T3: 39/28 °C (12 h L/12 h D); T4: 28/39 °C (12 h L/12 h D); T5: 25/25 °C (16 h L/8 h D). Shoot tip culture (7–10 mm) on AP medium with 6 mg/L BA.	Survival rates of Hermosa’/’Summerset’: T1: 2%/5%; T2: 58%/52%; T3: 15%/10%; T4: 47%/49%; T5: 100%/100%. [137]
Potato, *Solanum tuberosum* L., ‘Baraka’, PVY	Nodal cuttings (1.0 cm) MS with 1 mg/L thiamine, 100 mg/L myo-inositol, 2 mg/L glycine, 30 g/L sucrose; 8.0 g/L agar; 0.001 mg/L NAA, 1.0 mg/L KIN, 0.1 mg/L GA_3_. Culture: 25 ± 2 °C, 16 h, 110 µmol m^−2^ s^−1^. T: 1st cycle: to a continuous light regime and T: of 37 ± 2 °C, for 40 days. Plants remained infected: sub-cultured many times, 25 ± 2 °C, 2nd cycle: 37 ± 2 °C, 16 h, 110 µmol m^−2^ s^−1^ for 30 days.	Regeneration rate: 1st cycle: 77.0%. [122]
Grapevine, *Vitis vinifera*, clones of Napoleon: 29-228, 39-29, 74-16, 77-266, GLRaV-3, GFLV	Thermotherapy in heat chamber: T was increased gr. from 22 °C to 37.7 °C (increase by 4 °C on every 5th day, during 20 days). Exposure for total 1.5 month to alternate temperature (37.5/34 °C (16/8)). 150 µE m^−2^ s^−1^, RH 80%. Then nodal sections (3–5 mm) with the 1st, 2nd, or 3rd AX buds were cultured in vitro: MS with 2.0 mg/L BA, 30 g/L sucrose, 7 g/L agar, culture in 23 ± 2 °C, 16 h, 30–35 µE m^−2^ s^−1^, RH 55–60%, 6 wk.	Survival rates of clones (control/heat treated): 29-228: 81.5%/64.8%; 74-16: 72.2%/58.8%; 77-266: 72.7%/52.9%. Explant types (22-228 clone): 1st bud: 59.6%/64.8%; 2nd bud: 82.5%/80.0%; 3rd buds: 93.5%/80.0%. [119]
Myrobalan, *Prunus cerasifera* var. *divaricate* Borgh, ACLSV, PNRSV; Plum, *Prunus domestica* L., ‘Empress’: PNRSV; Sweet cherry, *Cerasus avium* (L.) Moench., ‘Early Rivers’, PDV	In vitro shoots (cultured on MS with 0.5 µM IBA, 5.0 µM BA, under 24/21 °C, 16 h, 2000 lux) heat treated: temperature gr. increased from 28 to 36 °C within a week and kept at 36 °C for four weeks. Post-culture: shoots on the fresh medium 24/21 °C for 4 weeks, then shoots on the rooting medium with 2 mg/L IBA. Potted and kept in greenhouse.	Survival rates of genotypes: myrobalan 80%; ‘Empress’ 66.7%; ‘Early Rivers’ 100%. [135]
Potato, *Solanum tuberosum* L., ‘Tsaeda embaba’, PVX, PLRV, PVS, and their co-infection	CMS with 2% sucrose, 6.5% agar, culture at 20/18 °C, 16 h, 55 µmol m^−2^ s^−1^, sub-culture: 3–4 wk. In vitro plants: at 37 °C, 16 h, for 1–4 wks. Survivor sub-cultured, meristems (0.5 mm) excised and onto MS with 2 mg/L glycine, 100 mg/L myo-inositol, 0.5 mg/L nicotinic acid, 0.50 mg/L pyridoxine HCl, 0.10 mg/L thiamine HCl, 2% sucrose, 6.5% agar, 0.01 mg/L BA. 27/20 °C, 16 h, 55 µmol m^−2^ s^−1^. Regenerated plantlets: medium without PGR, sub-cultures at 27/20 °C, 16 h, 55 µmol m^−2^ s^−1^.	Survival rates: Treatment for 1 wk: 90%; for 2 wk: 55%; for 3 wk: 0%. [139]
Raspberries, *Rubus idaeus* L., Z13 and virus-free cultures of line TTA-508, RBDV	4-week-old shoots (>2 cm) on MS with 100 mg/L myo-inositol, 30 g/L sucrose, 0.5 mg/L BA, 0.05 mg/L IBA, 3.5 g/L Bacto agar, 1.2 g/L Gelrite. Culture: 22 ± 2 °C, 16 h, 45 μE s^−1^ m^−2^ for 3 days. Then 38/26 °C, 16 h L/8 h D for 21–42 days. Meristem culture: 0.2 mm (2 LP) cultured for 3 d on MS with 100 mg/L myo-inositol, 30 g/L sucrose, 0.5 mg/L BA, 0.05 mg/L IBA, 3.5 g/L Bacto agar, 1.2 g/L Gelrite, 2.5 g/L AC, then transferred onto the same medium without AC for regeneration. 22 ± 2 °C, 16 h, 45 μE s^−1^ m^−2^.	Survival rates: after thermotherapy: 21 d: 62%, 42 d: 6%. Survival/regeneration rate after thermotherapy + meristem culture: 21 d: 95%/90%; 42 d: 32%/38%. [26]
Potato, *Solanum tuberosum* L., ‘Diamond’ PVY	In vitro shoot culture on MS medium with 0.2 GA_3_, 30 g/L sucrose, 25 ± 2 °C, 16 h, 2500 lux. 1st cycle of heat treatment: 37 ± 2 °C, for 40 days, then sub-culture at 25 ± 2 °C, 16 h, 2500 lx. 2nd cycle: 37 ± 2 °C for 30 days.	Survival/regeneration rates: 1st cycle: 42.8%/74.1%. 2nd cycle: n.a, but at least 14.2% regenerated. [121]
Sweet potato, *Ipomoea batatas* L., ‘Tanzania’, ‘New Kawogo’, ‘Busia’, CIP clone: 199004.2, SPCSV, SPFMV, SPMMV	Plantlets exposed to temperature regimes of 32/28 °C; 36/32 °C; 40/34 °C (16/8 h L/D) for 4 wk. Then AP meristem (0.5–1.0 mm, 1–2 LP) culture: on MS, 16 h, for 5 wk.	Regeneration rates of cultivars: ‘Tanzania’: 77%; ‘New Kawogo’: 78%; ‘Busia’: 82%; CIP clone: 199004.2: 70%. Survival rates after T regimes: 32/28 °C; 80.5%; 36/32 °C: 97.2%; 40/34 °C. 75.5%. [120]
Potato, *Solanum tuberosum* L., ‘Burren’, ‘Binella’, PVY	Plantlets exposed to 37 ± 2 °C, continued L (5 μm m^−2^ s^−1^), 40 d, then AP meristems (100–200–300 μm) isolated and cultured on MS, with 2 mg/L glycine, 5 mg/L nicotinic acid, 5 mg/L pyridoxine, 5 mg/L thiamine, 5 mg/L ascorbic acid, 200 mg/L myo-inositole, 2.0 mg/L GA_3_, 0.2 mg/L KIN, 3% sucrose, 0.6% agar.	Survival rate: heat treated/control, in average of cultivars: 100 μm: 88%/87%; 200 μm: 94%/97%; 300 μm: 100%/100%. [54]
Sand pear, *Pyrus pyrifolia*, Burm. ‘Jinshui no. 2’, ACLSV, ASGV	In vitro cultures on MS with 1.0 mg/L BA, 0.2 mg/L IBA, 30 g/L sucrose, 5.3 g/L agar. Growing: 24 ± 1 °C, 16 h, 40 µmol m^−2^ s^−1^. Sub-culture: 30 d. Shoots (7.0 mm) on fresh MS culture: in growing room for 2 ds, then into a heat chamber (16 h, 40 µmol m^−2^ s^−1^). T raised gr. 24 ± 1 °C to 35 ± 0.5 °C in 4 d. 40 d, Meristem (1.0–0.5 mm). ST from five main shoots and axillary shoots were cultured on MS with 1.0 mg/L BA, 0.2 mg/L IBA, 30 g/L sucrose, 5.3 g/L agar. Growing: 24 ± 1 °C, 16 h, 40 µmol m^−2^ s^−1^. 2 cycles of sub-culture.	100% survival, regeneration rates: 1.0 mm/0.5 mm explants: control: 90.9%/85.7%; treated: 62.5%/66.7%. [4]
Potato, *Solanum tuberosum* L., ‘Diamant’, ‘Heera’, ‘Lalpakri’, PVY	Meristems (0.2–0.5 mm) on MS + PGRs combinations (mg/L) (BA 0 − 1.5 − 3.0 − 4.5 + GA_3_ 0 − 0.2 − 0.4 − 0.6; 0.0 + 0.0 (control)). Culture: 25 ± 1 °C, 16 h, 2000–3000 lux, RH 60–70%. Immediately after excision for 60–65 days. Heat treatment: 27 ± 1 °C (control), 30 ± 1 °C, and 35 ± 1 °C.	Survival rate: T: 27 ± 1 °C (control): 24.55%; 30 ± 1 °C: n.a.; 35 ± 1 °C: 20.47%. Varieties: ‘Diamant’: 19.1%; ‘Heera’: 22%; ‘Lalpakri’: 25.9%. PGRs: All BA free media: 0%. The best results: 1.5 mg/L BA + 0.2 mg/L GA_3_: 39.7%; 3.0 mg/L BA + 0.2 mg/L GA_3_: 46.1%; 4.5 mg/L BA + 0.2 mg/L GA_3_: 42.4%. [132]
Artichoke *Cynara cardunculus* L. var. scolymus, AILV, ArLV	Meristem (0.3–0.5 mm) culture on MS, then heat treatment: 28 °C: 1st and 2nd day; 30 °C on 3rd day, 38 °C from 4th to 14th days; 36 °C from 15th to 18th days, 34 °C from 19th to 21st days, 32 °C from 22nd to 23rd days, 30 °C from 24th to 25th days, 28 °C from 26th to 28th days, and finally 26 °C at the last (29th).	Survival rates: AILV infected plant: 90.9%, ArLV infected plant: 6.5%. [123]
Grapevine, *Vitis vinifera* L., ‘Manto Negro’ clone (MPL15.01), GFkV	ST culture, established on MS with 3% sucrose, 0.7% agar (PGR-free; MS-0), or with 2 mg/L BA (MS-BA). Field therapy: in July: in average T not below 30 °C, reaching 39 °C at least for 3 d, not below 16 °C at night. For ST (1–3 mm) culture: MS-0 and MS-BA. Growing: 23 °C, 16 h, 56 μmol m^−2^ s^−1^, RH 60%, 5 wks, then rooting (1/2 MS). Chamber heating: 5-week-old greenhouse plant into heat chamber, 26 °C/22 °C (L/D), 16 h, 56 μmol m^−2^ s^−1^. T increased by 4 °C/wk., for 40 d. Then: AP and AX buds isolated and cultured.	Regeneration rates: MS-0/MS-BA: Field therapy: 33%/16%, Chamber therapy: 38%/10%. [138]
Apple, *Malus domestica* Borkh, ‘Xinhongjiangjun’ACLSV, ASPV, ASGV	Shoots (6.0 mm) onto fresh MS in growth room for 2 days, then into a heat-chamber (16 h L/8 h D 2000 lux). T raised gradually: up to 34 ± 0.5 °C, 36 ± 0.5 °C, and 38 ± 0.5 °C for 20 days. Meristem 1.0 mm from AP and AX shoots after treatments. Culture: MS with 1 mg/L BA, 0.1 mg/L NAA, 30 g/L sucrose, and 5.6 g/L agar, 24 ± 1 °C, 16 h, 2000 lux.	Survival rates: 34 °C: 100%, 36 °C: 93%; 38 °C: 40%. Regeneration rates: From AP: 34 °C: 70%; 36 °C: 96.4%; 38 °C: 8.3%. From AX: 34 °C: 72.9%; 36 °C: 88.2%; 38 °C: 0%. [140]
Apricot, *Prunus armeniaca* L., cultivar: n.a., PPV	Thermotherapy on in vitro shoots: 37 °C for 3, 4, 5, 6, and 7 weeks. Then ST onto proliferation medium (MS with 2 mg/L BA, 0.5 mg/L GA_3_) at 25 ± 2 °C, 16 h, 36 μM m^−2^ s^−1^.	Survival rates after 3 wks: 100%. Regeneration rates after 1st sub-culture: 60%; after 2nd sub-culture: 0%. Survival rates after 4 weeks: 40%; regeneration rates after 1st sub-culture: 20%; after 2nd sub-culture: 0%. After 5, 6 and 7 weeks: all died. [86]
Potato, *Solanum tuberosum* L., ‘Kinigi’, ‘Rwangume’, ‘Victoria’, PVX, PVS	Plantlets at 37 to 40 °C (16 h, 10,000 lux) and at 30–34 °C (8 h D) for 2, 3 or 4 wks in a heat chamber. AP meristem (~0.2 to 0.5 mm) on medium with 1.0 mg/L GA_3_, 0.4 mg/L BA, 100 mg/L ascorbic acid, 6 g/L agar, 100 mg/L myo-inosotol, 1 mL/L folic acid, 4 mL/L *L*-Arginine, 30 g sucrose.	Survival rates of varieties infected by PVS/PVX: ‘Kinigi’: 54.6%/64.6%, ‘Rwangume’: 75.4%/61.7%, ‘Victoria’: 79.6%/67.1%. [133]
Cassava, (*Manihot esculenta* Crantz), Tanzanian landrace, EACMVs	In vitro shoot culture: MS with 20 g/L sucrose, 3 g/L agar, 28 °C, 16 h, sub-culture 5 wk. 2-week-old plantlets into heat chamber: 30, 35, 40 °C (control: 28 °C), for 3 weeks, 16 h.	Survival rates: Control: 100%; 30 °C: 93.8%; 35 °C: 81.3%; 40 °C: 47.9%. [128]
Apple *Malus domestica* Borkh, ‘Gala’, ‘Fuji’, ‘Ruixue’, ‘Nongguo 25’, *Malus pumila paradisiaca* L.: ‘M9’, ASGV	Thermotherapy: MS + 0.25 mg/L BA, 2-wk-old shoot growth chamber 36/32 °C, 16 h, 50 µEs^−1^m^−2^.	Survival rate after thermotherapy: 100%. Shoot length: treated: 2.5 cm; control: 3.5 cm. Multiplication rate: treated: 4.7 shoot/explant, control: 2.5 shoot/explant. [38]
Apple, (*Malus domestica* Borkh) ‘Gala’, ASGV	Thermotherapy (36/32 °C, 16 h, 50 µEs^−1^m^−2^.), then shoot tip isolation: 1.5 mm (4–5 LP), 0, 2, 4, and 6 wks after thermotherapy for recovery, regeneration on MS ± 0.25 mg/L BA, 24 ± 2 °C, 16 h, 50 µEs^−1^m^−2^. Sub-culture 4 wks, rooting 0.5 mg/L NAA for 4 weeks, planted to soil.	Regeneration rates after different exposure time: 0 week: 100%, 2 weeks: 88.9%, 4 weeks: 77.8%, 6 weeks: 64.5%. [38]
Grapevine, *Vitis champinii* Planch, GLRaV-3	Plantlets on ½ MS, 24 °C, 16 h, 2000 lux, sub-culture: 50 d, shoots (1.0 cm) on fresh 1/2 MS for 10 d normal growing condition, then 16 h, 2000 lux, T increased gr. to 37 °C, for 20 days. AP and AX shoots (1.0 and 0.5 mm), culture on ½ MS, 5× sub-culture.	Plantlets higher than control in the whole period. Survival rate: 100%. Regeneration rate: 23.3%. Other: leaf discoloration, necrosis, wilting. [127]
Shallot, *Allium cepa* var. aggregatum, G. Don, 10603, OYDV, SLV	In vitro shoots on MS with 30 g/L sucrose, 0.5 mg/L BA, 0.1 mg/L NAA, 8 g/L agar. Culture: 22 ± 2 °C, 16 h, 50 µmol s^−1^ m^−2^. Sub-culture: 4 wk. 4-week-old in vitro shoots to thermotherapy for 0, 2, and 4 weeks, at a constant T of 36 ± 1 °C, 16 h, 50 µmol s^−1^ m^−2^. Then meristems (0.5 mm, 1–2 LP), on MS with 30 g/L sucrose, 0.5 mg/L BA, 0.1 mg/L NAA, 8 g/L agar. Culture: 24 ± 2 °C, D for 3 days, then 24 ± 2 °C, 16 h, 50 µmol s^−1^ m^−2^. Sub-culture: 4 wk.	Survival/regeneration rates after exposure of plantlets for 0 week (control): 100%/55%; for 2 weeks: 85%/51%; for 4 weeks: 62%/32%. [116]

AC: activated charcoal; ACLSV: Apple chlorotic leaf spot virus; AILV: Artichoke Italian Latent Virus; AP medium: Almehdi & Parfitt (1986) [141]; AP: Apical; ArLV: Artichoke Latent Virus; ASGV: Apple stem grooving virus; ASPV: Apple stem pitting virus; AX: Axillary; BA: 6-benzyladenine; CMS medium: [142]; d(s): day, days; D: Darkness; EACMVs: East African cassava mosaic viruses; GA_3_: Gibberellic acid; GFkV: Grapevine Fleck Virus; GFLV: Grapevine Fanleaf Virus; GLRaV-3: Grapevine Leafroll-associated Virus; gr: gradually; h: hour; IBA: Indole-3-butyric acid; KIN: kinetin; L: Light; LP: Leaf primordium(a); MS: [62]; n.a.: not available; NAA: α-naphthylacetic acid; OYDV: Onion Yellow Dwarf Virus; PDV: Prune Dwarf Virus; PGR: Plant Growth Regulator; PLRV: Potato Leaf Roll Virus; PNRSV: Prunus Necrotic Ring Spot Virus; PPV: Plum pox potyvirus; PVS: Potato Virus S; PVX: Potato Virus X; PVY: Potato Virus Y; RBDV: Raspberry Bushy Dwarf Virus; RH: Relative humidity; SLV: Shallot latent virus; SPCSV: Sweet potato chlorotic stunt virus; SPFMV: Sweet potato feathery mottle virus; SPMMV: Sweet potato mild mottle virus; ST: Shoot tip; T: temperature; wk(s): Week(s).

### 4.4. The Effect of Temperature and Exposure Time

During thermotherapy of apple (*Malus domestica* Borkh., ‘Xinhongjiangjun’) by 34 ± 0.5 °C, all plantlets grew normally [140]. Leaves started to yellow, to curl, and became to be chlorotic at 36 ± 0.5 °C, but the majority of plantlets survived until the end of the experiment. However, survival and shoot proliferation was significantly reduced by 38 °C treatment; low survival rate (40–46.7%) was observed, and axillary bud explant did not regenerate the shoot at all [140]. Survival rates of potato (*S. tuberosum* L.) plantlets in the average of three varieties (‘Diamant’, ‘Heera’, ‘Lalpakri’) were very low, and a slight decrease from 24.6% (27 ± 1 °C) to 20.5% (35 ± 1 °C) was detected when thermotherapy was applied for a 60–65 day period during development of plantlets from isolated meristem [132]. When higher temperature (37 °C) was applied for a shorter period (40 days) on in vitro plantlets, the survival rates in the average of different sized meristem isolated from treated plantlets were high (93.3% and 96.0%) in the case of ‘Binella’ and ‘Burren’ potato (*S. tuberosum* L.) cultivars, respectively [54].

During thermotherapy of the cassava (*Manihot esculenta* Crantz, Tanzanian landscape cultivar), the survival rate decreased with increasing temperature, while 100% of the plantlets survived in the control treatment, the plantlets treated by 30, 35, and 40 °C temperature survived in rate of 93.8%, 81.3%, and 47.9%, respectively [128].

The best regeneration rate (97.2%) was obtained when thermotherapy occurred by 36/32 °C, following the heat-treatment by 32/28 °C resulting in 80.5% regeneration in sweet potato (*I. batatas* (L.) Lam). Application of higher temperature in alternate regime (40/34 °C) restricted the regeneration ability of plantlets to 75.5% [120].

As several species grown in temperate zone are sensitive to rapid temperature rises, a gradual increase in temperature is recommended [124]; for example, a dual regime of temperature (37.5/34 °C for 16 h/8 h light/dark period) after gradual increase of temperature (4 °C on every fifth day) was suggested for grapevine clones (*Vitis vinifera* L, Napoleon clones see Table 3) [119]. Survival and regeneration capacity of shallot (*Allium cepa* var. *aggregatum* G. Don, 10603) decreased significantly as the duration of treatment (36 °C) increased from zero to four weeks; 85% of the plants survived the two-week-long treatment, and 51% of the meristems regenerated shoots. Survival rates were 62% in four weeks of treatment, and only 32% of meristems were able to regenerate shoots [116]. By increasing the duration from three to seven weeks of treatment at 37 °C, the survival rate of in vitro apricot (*Prunus armeniaca* L.) shoots was reduced. Maximum (100%) survival was obtained only after three weeks of treatment; the survival rate was already significantly reduced (down to 40%) in four weeks of treatment, and no plants survived the treatment lasted for more than four weeks. In addition, regenerative capacity did not increase during sub-culture (at 25 °C for four weeks) but decreased further, and all shoots died after second sub-culture [86]. Extension of heat treatment (38/26 °C) from 21 days to 42 days significantly determined the survival of raspberry (*Rubus idaeus* L., Z13) shoots (62% and 6%, respectively). When isolated meristems were cultured after thermotherapy, the survival and regeneration ability of meristems were also significantly affected by exposure time: after treatment for 21 days, 95% survival and 90% regeneration ability were observed. When thermotherapy lasted for 42 days, only 32% of explants survived, and 38% of them were able to regenerate shoots [26]. After one week of treatment by 37 °C, the survival rate of potato (*S. tuberosum* L., ‘Tsaeda embaba’) plantlets dropped to 90%; after two weeks, only 55% were alive, and by the end of third week, all had died [139]. By the end of the second week, there were already serious symptoms of heat stress: the leaves were curled up and almost facing the stem, pointing upwards, and the edges of the leaves turned yellow. By the second week, the shoots were already so dehydrated that it was very difficult to isolate the meristem: they were tiny and stuck to the isolating needle. However, the development of isolated meristems was quite rapid (regenerated in average in 38 days), which was attributed to the fact that the culture occurred at a relatively high temperature (27/20 °C) [139]. Although all sand pear (*Pyrus pyrifolia* Burm, ‘Jinshui no. 2’) shoots survived the heat treatment (35 ± 0.5 °C, for 40 days), shoots suffered from heat stress as exposure time went on. During the first 5–20 days, treatment stimulated the growth of in vitro shoots, but if exposure lasted 25–40 days, the plant growth was inhibited. The leaves often turned yellow or even blackened in the first 5–35 days, but with the development of new leaves, a gradual recovery in growth was observed [4].

### 4.5. Suggestions for Thermotherapy Applications

It is very important to make a decision about application of thermotherapy depending on the plant species, which required virus elimination. For example, plant species flowering in early spring and spending the summer in dormancy are unlikely to be treated with this method. In terms of heat sensitivity, there are very large differences between species, which must also be taken into account. The type and localization of the virus also determines whether it is worthwhile to perform heat treatment. Moreover, virus infected, weak plants can be more sensitive to high temperature, and the age of plantlets or in vitro cultures can play role in the heat tolerance as well. However, stress tolerance can be increased by application of pre-treatment chemicals that play a role as signaling molecules in stress reduction, such as SA or H_2_O_2_. Selection of the proper temperature and exposure time, which ensure an adequate balance between the rates of virus degradation and plant damage, requires extensive experiments, but it is worth doing. Temperature can be increased gradually, if necessary, and alternate temperature regimes can be used with success as well. Changing the composition of the medium, especially PGRs, can result in higher survival. However, the high survival rate is not enough, because the regeneration ability can be inhibited by thermotherapy even after more than one sub-culture.

## 5. Virus Elimination by Electrotherapy

### 5.1. The Background

Unlike the methods described so far, the antiviral effect of electrotherapy is based on the destruction of virus particles leading to loss their infectivity, but not on the isolation of uninfected plant parts [143,144]. This phenomenon was already achieved with a very short (2 min) treatment of 5 and 100 mA on purified preparations of almond mosaic virus isolate [143].

Despite the fact that the temperature of the electrotherapy treated plant parts can increase significantly during the process [32], the basis of virus eradication is probably not only due to the elevated temperature as it was concluded by Quaquarelly et al. [143]. Although in their experiments, the virus eradication was only successful if the temperature of treated plant parts increased, the same plant material (cuttings of almond infected by mosaic virus) treated by the same temperature (37–38 °C) in water bath remained infected.

Moreover, the sensitivity of viruses for electric current can be different [31,145], maybe due to their different structure (size, geometry, and biomolecular configuration) [146,147].

Electrotherapy can be used on any kind of plant part (stem segment, shoot tip, cane piece, plantlet, cornel, sprout, etc.) for virus elimination with high effectivity [27,144,148]. The process is very simple and cheap [149], does not required any specific equipment [144], and only takes a few minutes (from 5 to 25 min) [150]. Electrotherapy of plant parts immersed in solutions of Tris Acetate EDTA (TAE) or Tris Borate EDTA (TBE) buffer (mostly sodium chloride solution) is realized most frequently in an electrophoresis tank by application of power supply [31,34,121,151]. Electric current from 5 to 100 mA has been used for virus eradication, although the most common treatment is in a range of 10–25 mA (see Table 4). After a short period (5–20 min) of treatment tissue cultures are established following the surface sterilization, and regeneration occurs under in vitro condition.

In several experiments, the regeneration capability of treated plant parts was stimulated by electric treatments, maybe because of increased responses to the chemical signals and increased uptake of the resources from medium (PGRs, ions) [152,153]. Since electric field can be amplified by membranes [154], all membrane-located processes (for example hormonal signal transduction, uptake of ions and water) can be modified by an electric treatment [153]. Alteration of the amplitude or frequency of the own electric patterns of plant cells—as a result of interaction between exo- and endogenous electric fields—can also play role in further development [153,155]. In order to increase effectiveness, electrotherapy can be combined with other treatments, such as chemotherapy [27,144].

### 5.2. The Effect of Explant and Genotypes on Regeneration Ability of Treated Plant Parts

Regeneration ability of potato (*S. tuberosum* L.) explants (shoot tips, nodal segments, and tuber sprouts) treated by electric current (5, 10, 15, 20, and 25 mA for 5, 10, 15, 20, and 25 min) was studied by Singh and Kaur [150], and they found the best responses in tuber sprouts, because their viability was very similar to the control ones (>90% regeneration). The regeneration ability of shoot tip explants was also hardly affected (82.6–88.2%; control: 87.5%), while regeneration from nodal segments was decreased down to 76.4% from 90.97% (control). All regenerated plantlets showed normal development [150].

Some species proved to be sensitive for higher electric current, such as potato (*S. tuberosum* L.) [145], which is often involved in electrotherapy experiments, although responses of potato cultivars to electrotherapy were also different [31,32,33]. Breeding lines and wildtype potato stem segments were very sensitive for electric current of 5, 7, and 10 mA, and these treatments resulted in 58.3%, 50.0%, and 34.6% regeneration rates, respectively [145]. ‘Mexiquense’ and ‘Norteña’ potato cultivars were also sensible to the highest electric current (15 mA) [32]. Although the survival was affected by the size of excised meristem (the larger the explant the greater the survival rate), in general high survival rates could be achieved by potato cultivars ‘Binella’ and ‘Burren’ (in a range of 75–94% and 75–100%, respectively) when in vitro plantlets (in 12–15 cm length) were treated by 15 mA [54]. Moreover, electric treatments can enhance the regeneration of potato as it was detected in the case of clone 760055, when 20% regeneration rate was observed in control and 25–88% in plants treated with electric current. The other two clones (750615 and 750783) also mostly showed greater regeneration rates after electric treatments compared to control, but not in all treatments, and no clear trend could be detected [32].

### 5.3. The Effect of Electric Current Intensity and Duration of Treatments

The mean regeneration rates of potato (*S. tuberosum* L.) cultivars were decreased by electric current from 91.6% to 68% (10 min. treatment) and to 51.3% (20 min. treatment). Treatments for 20 min resulted in lower regenerative capacity of buds. The higher the exposure time or the intensity of electric current are, the lower the regeneration rate is. However, when the exposure time was only 10 min, the highest regeneration rate (75%) was detected after 25 mA treatment [31]. Similarly, lower regeneration rates could be observed after 20-min-long treatment of potato (*S. tuberosum* L.) (‘Roclas’) in each intensity (40, 50, and 100 mA) compared to shorter exposure, while no significant differences could be detected between treatments lasted for 5 and 10 min [156]. Interestingly, application of 100 mA on PVY infected potato stem segments inhibited the regeneration rates down to 50–59.5%, while PVX infected ones responded better (66.7–77.2% regeneration rates) [156].

When Mahmoud et al. [121] applied the electric treatment directly onto stem segments of potato (*S. tuberosum* L.) by connecting them to electrodes, they observed stimulatory effect on the regeneration: while control buds regenerated in 45.5%, treated explants reached 63.3% regeneration. Moreover, increasing current (from 5 to 15 mA) resulted in increasing regeneration capacity (from 48.8% to 63.3%) in the 5-min-long treatments. However, the highest current (15 mA) applied for a longer time (for 10 min) already reduced the regeneration rate down to 24.0%. In contrast, in the indirect treatment (applied on two-node stem pieces submerged in sodium chloride solution in electrophoresis chamber for 5 min), the regeneration capacity was rather reduced (from 31.1% to 17.9%) by increasing the current (from 5 to 15 mA). Accordingly, a very low regeneration rate (3.2%) was observed in the 10-min-long treatment by 15 mA current, although the lowest current showed a similar result to the control (30%) [121].

Treatment of grapevine (*Vitis vinifera* L., ‘Black’) by 10, 20, and 30 mA electric current for 10-min-long reduced the regeneration rate from 100% (control) to 83% and 75% (10 mA and both 20 and 30 mA, respectively) [92]. Longer treatment (for 15 min) resulted in a further decrease in regeneration rates (down to 75–62.5%). No differences were found in regeneration of buds after 20 and 30 mA treatments. Some regenerated plantlets were morphologically abnormal [92]. In contrast, no differences were detected in the morphology of potato (*S. tuberosum* L.) between treated and non-treated ones [32].

Responses of gladiolus (*Gladiolus communis* L.) cultivars (‘Aldebaran’, ‘Tiger Flame’, ‘Vink’s Glory’) were very similar to each other, when treated by electric current for 20 min. However, as the current intensity increased (from 10 to 20 and 30 mA) the regeneration rates decreased (from 74.7% to 65.3% and 56.0%, respectively) [157]. Similar responses were detected in dahlia (*Dahlia* sp., L.) when stem segments were treated by 15, 25, and 35 mA, and treatments resulted in 89%, 80%, and 67.5% regeneration rates on average, respectively. Moreover, slightly lower regeneration rates were obtained after 20-min-long treatment (76% regeneration mean) compared to 10-min-long treatment (81.7% regeneration mean) [146].

Regeneration capability of common bean (*Phaseolus vulgaris* L.) nodal cuttings after electric current treatment was decreased, and as the current increased (from 5 to 15 mA), the frequency of regeneration decreased for both cultivars (‘Khomein’ and ‘Capsouli’) treated for 10 min [34]. Ramírez et al. [158] tested the sensitivity of yam (*Dioscorea cayenensis* subsp. *Rotundata* (Poir.) Miège.) tissue for electric treatments in a range of 5 to 50 V, and they found that voltages higher than 20 V significantly reduced the regenerative capacity, and even in the case of 50 V, they did not receive a regenerated shoot at all.

In addition to regeneration and survival rates, other developmental, morphological changes can be induced by electric treatments. Regeneration rates of malanga (*Xanthosoma sagitifolia* Schott) explants were increased by a 5-min-long electric treatment in a range of 5–20 V, while higher voltages (30–50 V) decreased significantly the regeneration ability of treated axillary buds. Longer duration (for 10 min) of treatments by 5–15 V resulted in similar rates to control ones, while higher voltage inhibited the regeneration significantly [149]. Moreover, electrotherapy stimulated the shoot growth; the longest shoots (6.1 cm) were observed after 20-min-long treatment by 20 V; it was almost twice the length of the control plants (3.3 cm).

Bădărău et al. [151] applied electric currents (40, 50, and 100 mA for 5, 10, and 20 min) on nodal segments of potato (*S. tuberosum* L., ‘Roclas’). They studied the behavior of treated explants during three sub-cultures, and they found significantly increased (minimum doubled) multiplication rates after electric treatments, independently of the virus elimination success. The number of leaves and the length of shoots were also enhanced, and these effects could be observed in each sub-culture. No adverse effects were observed, although high currents (up to 100 mA) were used for a relatively long time (up to 20 min).

**Table 4:** Results of virus elimination experiments performed by electric treatment.

Plant Species, Cultivar, Virus	Methods	Survival and/or Regeneration [Reference]
Potato, *Solanum tuberosum* L., Clones: 760055, 750615, and 750783, PVX	2-month-old plants from greenhouse, segments (AX buds) treated: current intensity: 5, 10, or 15 mA, duration: 5 or 10 min. Then ST (0.5–1.0 mm) culture on MS salts with 0.25 ppm GA_3_, 2.0 ppm calcium pantothenate, 3% sucrose, 0.8% agar (30 d). Transfer to medium MS salts with 0.3 ppm IAA, 0.3 ppm KIN, 4% sucrose, and 7% agar. 14 h, 35 µE m^−2^ s^−1^, 25–28 °C (30 d).	Regeneration rates control/treated: 760055: 20%/67%; 750615: 20%/34.2%; 750783: 13%/18.8%. [32]
Malanga, *Xanthosoma sagitifolia* Schott, ‘Mexico 8’ clone, DMV	Treated by 5, 10, and 20 V for 5 min. Culture: 70% MS with 100 mg/L myo-inositol, 0.1 mg/L BA, 30 g/L sucrose (60 d), then: MS with 100 mg/L myo-inositol, 1.0 mg/L IAA, 3 mg/L BA, 30 g/L sucrose, 5 g/L agar.	Regeneration rates/growth: control: 35%/3.3 cm; 5 V: 75%/4.1 cm; 10 V: 70%/4.5 cm; 20 V: 20%/6.1 cm. [149]
Potato, *Solanum tuberosum* L., 94P70-4, 93P29-3, 93P42-1, 89L92-3, 89L92-4 lines and ‘Chuncheonjerae’ (wild type),PVX, PVY, PLRV	Stem segments (6 AX buds): 5, 7, and 10 mA for 5 min. Culture on MS with 0.2 mg/L GA_3_, 0.04 mg/L KIN, 0.1 mg/L IAA, and 30 g/L sucrose. Sub-culture: 8 wks, 35 µE m^−2^ s^−1^, 16 h, 23 ± 1 °C.	Regeneration rates: 5 mA: 58.3%; 7 mA: 50.0%; 10 mA: 34.6%; control: 92.3%. [145]
Potato, *Solanum tuberosum* L., ‘Diamond’, PVY	Stem (5 nodes): directly connected to the electrodes: 5; 10; and 15 mA for 5 or 10 min. Stem (2 nodes): in the electrophoresis chamber with NaCL solution, 5; 10; and 15 mA (indirectly). ST (1.0 mm) from AX buds from treated stems: onto MS with 0.1 mg/L IAA, 0.2 mg/L GA_3_, with or without 20.0 mg/L RBV, 30 g/L sucrose, and 7.0 g/L agar. Culture: 25 ± 2 °C, 4–6 wks, then multiplication (same medium without IAA) and rooting (same medium with 0.04 mg/L KIN): 25 ± 2 °C, 16 h, 2500 lux.	Regeneration rates: Directly: with RBV: 24–34.3%, without RBV: 24.0–63.3%. Indirectly: with RBV: 0–27.0%, without RBV: 3.2–31.0%. [121]
Common bean, *Phaseolus vulgaris* L., ‘Khomein’ and ‘Capsouli’, BCMV	Stem segments with AX buds in TAE buffer exposed to electric currents of 5, 10, and 15 mA (10 min). Nodal cuttings cultured on MS salts with B5 vitamins, 16 h, 23–25 °C for 30 d.	Regeneration rates: ‘Khomein’: control: 85%; 5 mA: 80%; 10 mA: 65%; 15 mA: 53.6%, ‘Capsouli’: control: 90%; 5 mA: 78.3%; 10 mA: 71.4%; 15 mA: 63.4%. [34]
Potato, *S. tuberosum* L., ‘Banaba’, ‘Olimpya’, ‘Agria’, ‘Desirea’, ‘Lady Roseta’, Clone 69, PVA, PVY	Stems (3-5 AX buds) from plants grown in greenhouse: treated by electric currents 15; 25; and 35 mA; for 10 or 20 min. Then AX bud culture: ½ MS, PGR-free (10 d). Sub-culture: full MS, 16 h, 23/18 °C, 60% RH, 54 μmol m^−2^ s^−1^.	Regeneration rates: ‘Banaba’: 66.6%; ‘Olimpya’: 54.1%; ‘Agria’: 58.3%; ‘Desirea’: 54.1%; ‘Lady Roseta’: 70.8%; Clone 69: 54.1%. [31]
Grapevine, *Vitis vinifera* L., ‘Black’, GVA	Green cane pieces, (3 cm with a bud), leaves removed, exposed to electric currents: 0, 10, 20, and 30 mA, for 10 or 15 min. in TBE buffer (90 mM Tris-borate, 2 mM EDTA, pH 8). Culture: nodal explants on MS with 1 mg/L BA, 0.5 mg/L NAA, 50 mg/L AscA, 50 mg/L AceA, 30 g/L sucrose, 8 g/L agar. 2 months.	Regeneration rates: %, 10/15 min: control: 100%; 10 mA: 83%/75%; 20 mA: 75%/62.5%; 30 mA: 75%/62.5%. [92]
Potato, *Solanum tuberosum* L., ‘Burren’, ‘Binella’PVY	Shoot culture: MS, 30 g/L sucrose, 7 g/L agar, 25 ± 2 °C, 16 h, 2.5 µmol m^−2^ s^−1^. Sub-culture: 20 d. Plantlets (12–15 cm) in NaCl solution (1N), 15 mA for 5 or 10 min. Then: apical meristem (100–200–300 µm) culture. Regeneration: on MS, 30 g/L sucrose, and 0.6% agar.	Survival rates: 5/10 min.: ‘Burren’: 87.33%/85%; ‘Binella’: 88%/87.7%. [54]
Potato, *Solanum tuberosum* L., ‘Roclas’, PVX	Nodal cuttings: in sodium chloride solution (1M), electric currents: 40, 50, and 100 mA for 5-, 10-, and 20-min. Regeneration and culture: on MS. Sub-cultures 3x: after 26 ds, 30 ds, and 28 ds.	Multiplication rates: shoots/explant: Control: ~2.0–2.5; treated: from 4.0 up to 7.0. [151]
Potato, *Solanum tuberosum* L., ‘Roclas’, PVX, PVY	Nodal cuttings: in sodium chloride solution (1M), electric currents: 40, 50, and 100 mA for 5-, 10-, and 20-min. Regeneration and culture: on MS.	Mean regeneration rates: 5/10/20 min. 40 mA: 67%/68%/57%; 50 mA: 75%/71%/64%; 100 mA: 63%/68%/58%. [156]
Potato, *Solanum tuberosum* L., PLRV, PSTVd	Shoot tips, nodal segments, and tuber sprouts for treatment in TAE buffer, 5, 10, 15, 20, and 25 mA, for 5, 10, 15, 20, and 25 min. Treated nodal segments onto MS with 0.1 mg/L GA_3_, 0.1 mg/L NAA, 500 mg/L malt extract, 3% sucrose, and 0.8% agar. Sprouts and shoot tips onto MS with 2 mg/L KIN, 1 mg/L IBA, 500 mg/L malt extract, 3% sucrose, and 0.8% agar, 25± 2 °C, 70% RH, 16 h, 40 μM m^−2^ s^−1^.	Regeneration rates of nodal segments: 5 mA: 88.2–91.7%; 10 mA: 77.8–90.9%; 15 mA: 78.5–90.3%; 20 mA: 78.5–88.2%; 25 mA: 76.4–86.1%. Tuber sprouts: each ˂ 90%; shoot tips: 86.2–88.2%. [150]
Gladiolus, *Gladiolus communis* L., ‘Aldebaran’, ‘Tiger Flame’, ‘Vink’s Glory’, BYMV	Cormels (0.3–0.5 cm^3^) in 1X TAE buffer: electric currents of 10, 20, and 30 mA for 20 min. After treatment, on MS with 1.0 mg/L BA, 0.5 mg/L IAA, 2.0 mg/L 2,4-D, 3% sucrose. Culture: 23–25 °C, 16 h.	Regeneration rates (in average of cultivars): 10 mA: 74.7%; 20 mA: 65.3%; 30 mA: 56.0%. [157]
Yam, *Dioscorea Cayenensis* subsp. *Rotundata* (Poir.) Miège., “White guinea”, potyvirus	Experiments 1: evaluation of tissue sensitivity: nodal segments (1.5 cm) in 1% CA solution. 0 (control), 5, 10, 20, 30, and 50 V (direct current, DC) for 5 min. Experiments 2: Virus elimination: nodal segments immersed in 3.0% NaOCl for 20 min, 0, 5, 10, 15, and 20 V. Then in vitro culture.	Regeneration rates: Experiment 1: 0 V: 96%; 5 V: 91.6%; 10 V: 84.4%; 20 V: 56.3%; 30 V: 15.7%; 50 V: 0%. Experiment 2: 0 V: 100%; 5 V: 89.6%; 10 V: 87.5%; 15 V:75.0%; 20 V:43.8% [158]
Dahlia, *Dahlia* sp., L., DMV	Stem segments with 2 AX buds treated by electric currents of 15, 25, and 35 mA for 10 or 20 min. Culture: on MS with 2 mg/L BA, 0.25 mg/L GA_3_.	Survival rates: 10/20 min: control: 100%; 15 mA: 90%/88%; 25 mA: 85%/75%; 35 mA: 70%/65%. [146]

µE: microEinsteins; 2,4-D: 2,4-dichlorophenoxyacetic acid; AceA: acetic acid; AscA: ascorbic acid; AX: axillary; BA: 6-benzyladenine; BCMV: Bean common mosaic virus; CA: citric acid; d(s): day, days; DMV: Dahlia mosaic virus; EDTA: ethylenediaminetetraacetic acid; GA_3_: gibberellic acid; GVA: Grapevine virus A; IAA: indoleacetic acid; IBA: indole 3-butyric acid; KIN: kinetin; mA: milliampers; MS: Murashige-Skoog medium [62]; NAA: α-naphthylacetic acid; PLRV: Potato leafroll virus; PSTVd: Potato spindle tuber viroid; PVA: Potato virus A; PVX: Potato virus X; PVY: Potato virus Y; RBV: Ribavirin (1-β-d-ribofuranosyl-1, 2,4 triazone-3-carboxamide); RH: relative humidity; ST: shoot tip; TAE buffer: a buffer solution containing a mixture of Tris base, acetic acid, and EDTA; TBE buffer: Tris/Borate/EDTA, mixture of Tris base, boric acid, and EDTA; Tris/Borate/EDTA; Tris: tris(hydroxymethyl)aminomethane; wk(s): week(s).

### 5.4. Suggestions for Application of Electrotherapy

Because of its low harmful effect and high effectiveness, electrotherapy is one of the most promising means of virus eradication. Several plant parts can be chosen for treatment, and after application of an adequate electric current intensity and exposure time, high regeneration rate could be achieved with high rate of virus-free regenerants. However, few literatures can be found until now; thus, condition of treatments should be optimized for given genotypes and explants considering also the type of virus has to be eliminated.

## 6. Virus Elimination by Chemotherapy

### 6.1. The Background

*“…there are no ideal antiviral compounds and no ideal method for their evaluation.”*

Špak et al., 2010 [159]

Chemicals, which have antiviral activity and are named as antiviral drugs, can be divided into four groups according to their target as follows: (1) drugs that prevent the virus from entering to the host cell, (2) drugs that inhibit the virus genome replication, (3) inhibitors of protein synthesis, (4) and finally those that are protease inhibitors. Drugs belonging the first group preclude viruses from linking and entering into the host cell, while drugs involved in the second group are nucleoside or nucleotide analogues or non-nucleoside analogues, which inhibit the viral polymerases or retrotranscriptases in DNA or RNA viruses, respectively, thereby blocking the synthesis of nucleic acids [160]. Interferons belonging to the third group inhibit the protein synthesis required for viral replication, while protease inhibitors (fourth group) prevent the virus maturation [160].

In recent decades, at least 90 antiviral drugs have been categorized by their functionality into 13 groups [161]. Originally, each of these antiviral drugs was developed to treat human viral diseases (especially against human immunodeficiency virus, HIV). Although there are differences between animal and plant host cells, the similarity of certain pathways allows antiviral drugs to be used against plant viruses as well [28]. When 27 veterinary medicinal product drugs were tested for its antiviral activity against Cowpea chlorotic mottle virus (CCMV), Tobacco mosaic virus (TMV) in tobacco (*Nicotiana tabacum* L., ‘Xanthy’), and cowpea (*Vigna unguiculata* (L.) Walp, ‘California blackeye’); 14 of them, i.e., about 50% of them, were proven to be effective against these plant viruses [162].

In plant virus elimination experiments, several antiviral drugs were further tested including 2,4-dioxohexahydro-1,3,5-triazine (DHT) [163,164], 5-Azacytidine (AZA), and 3-Deazauridine (DZD) [122], acyclic nucleoside analogues [159], and so on. However, in most experiments, the Ribavirin (1-β-d-ribofuranosyl-1,2,4-triazole-3-carboxamide, RBV, trade names are Virazole, Copegus or Rebetol) was proven to be the most effective applied alone or in combination with other treatments [4,122,159,165].

#### 6.1.1. Mechanism of Actions of Nucleoside and Nucleotide Analogues

Ribavirin (discovered in 1972 [166]) is a broad-spectrum antiviral agent that is active against a number of RNA viruses, as well as several DNA viruses [167]. Its effect is manifested by several mechanisms, depending on the virus type [168]. Ribavirin is a guanosine analogue that interferes with guanosine 5′-monophosphate (GMP) biosynthesis due to the inhibition the formation of xanthosine 5′-phosphate from inosine 5′-phosphate (IMP) by preventing enzyme function of inosine 5′-monophosphate dehydrogenase (IMPDH) [169]. As a result, intracellular guanosine-5′-triphosphate (GTP) level can be reduced by up to 90% upon RBV treatment. Because both the host cell and virus require GTP and 2′-deoxyguanosine-5′-triphosphate (dGTP) for replication, this process is toxic to the host cell as well, but fortunately, viral replication is much more sensitive to a decrease in GTP levels than the host cell [168]. Upon entry into the cell, RBV is converted to ribavirin mono-, di-, and triphosphate (RMP, RDP, and RTP, respectively). Binding of RTP to the nucleotide binding site of RNA polymerase prevents the binding of the corresponding nucleotides, leading to reduced viral replication or the formation of defective virions (competitive inhibition) [170]. RTP is incorporated by viral RNA polymerase, resulting in viral mutation to a level that is “lethal” for the virus (“error catastrophe”) [171,172]. For certain RNA viruses, RBV may also act by preventing the 5′ end of the viral mRNA from being capped after translation. The cap prevents enzymatic degradation of the mRNA and aids in cap-dependent mRNA translation. When RBV takes the place of guanosine, it inhibits its methylation and thus the translation of mRNA.

Acyclovir (9-(2-hydroxymethyl)guanine, 1971) is also a nucleoside analogue, which [173] inhibits the DNA synthesis, after incorporation into DNA chain [174]. However, its effectivity and selectivity are depending on the virus, because it is phosphorylated by virus-specific enzyme (e.g., thymidine kinase) to create its active form [175].

As a result of later developments, further acyclic nucleotide analogues have been applied, e.g., Tenofovir (9-[(R)-2-(phosphonomethoxy) propyl]adenine, (R)-PMPA, 1995), Cidofivir (1-[(S)-3-hydroxy-2-(phosphonomethoxy) propyl]cytosine, (S)-HPMPC, 1996), Adefovir (9-[2-(phosphonomethoxy)ethyl]adenine, PMEA, 2002) [166], and PMEDAP (2,6-diamino-9-[2-(phosphonomethoxy) ethyl]purine)). They are phosphorylated by cellular kinases, resulting in competitive inhibitors of DNA polymerases [174].

Another often tested antiviral agent is the cytidine analogue named 5-Azacytidine [(4-amino-1-β-d-ribofuranosyl-s-triazin-2(1H), AZA, 1985]. AZA can be incorporated into RNA or DNA, after phosphorylation into 5′-mono-, 5′-di-, or 5′-triphosphate [176]. After incorporation of AZA into the DNA, a complex can be formed that can inhibit the DNA methyltransferase progression [177].

Similarly, to the other nucleoside analogues, the 2,4-dioxohexahydro- 1,3,5-triazin (or 5-azadihydrouracil, DHT) also can be converted to an active (phosphorylated) form [178], which may inhibit the viral reverse transcriptase [179].

The 3-Deazauridine (4-hydroxy-1-(β-d-ribopento furanosyl)-2-pyridone, DZD) is a uridine analogue. The main activity of this analogue is the inhibition of the synthesis of cytidine-5′-triphosphate (CTP); thus, inhibition of nucleic acids synthesis occurs without its incorporation into nucleic acids [180,181]. It also can be incorporated into the RNA chain under certain conditions, causing mutations [182].

Zidovudine (3′-azido-3′deoxythymidine, ZDV, synonym: azidothymidine; AZT, 1985) can be applied against several diseases including bacteria, fungi [183], and viral infections, especially HIV [166]. ZDV is a thymidine analogue, and its phosphorylated form (AZT-5′-triphosphate) can terminate the DNA chain during DNA polymerization [183]. Its incorporation was proven to be limited to viral DNA [184]. It was proven to be a competitive inhibitor of thymidine phosphorylation during TMP forming [185].

#### 6.1.2. Mechanism of Action of Other Antiviral Drugs Tested in Plant Virus Elimination

The oseltamivir (([(3*R*,4*R*,5*S*)-4-acetamido-5-amino-3-(1-ethylpropoxy)-1-cyclohexane-1-carboxylic acid)]), OSTV, 1997) is a neuraminidase inhibitor [166]. OSTV inhibits the release of virions from infected cells [174]. Its active form is the oseltamivir carboxylate [186], whose activity is highly selective to influenza virus [187]. Neuraminidase can be found in plants, animals, and microorganisms and on the surface of some members of Orthomyxoviridae [188] and Paramyxoviridae [189], but not in plant viruses. Thus, it is not expected to be effective against them, as was observed when it was applied alone [190]. However, using it in combined treatment with RBV was proven to be effective against potato (*Solanum tuberosum* L.) [156] and grapevine (*Vitis vinifera* L.) viruses [191].

Rimantadine [1-(1-adamantyl)ethylamine hydrochloride, 1964] inhibits the activity of viral polymerase [192], and the proton transport in the M2 protein channel (a surface membrane protein in influenza A) also was proven [193].

In recent years, a number of studies have focused on the usability of natural products including bee venom (venom of *Apis mellifera*). The main component of bee venom is a peptide, the melittin, which has shown antiviral activity against several human viral diseases [194] and against Tobacco mosaic virus (TMV) [195]. Several mechanisms of action of melittin can be supposed, including inhibition of viral replication, inducing a change of RNA conformation, destroying the virus particles directly, etc. [194].

Glycyrrhizin (synonym: glycyrrhizinic acid, GLY) is a triterpenoid saponin found in liquorice (*Glycyrrhiza glabra* L.) [196]. It can inhibit the penetration of a virus [197], maybe by its carboxyl group situated at 20-position [198]. It can also reduce the protein kinase activity [199].

Flavonoids are highly biologically active compounds that are widespread in plants and play a significant role in the plant interaction with the environment, including the defense mechanism elicited by biotic and abiotic stress [200]. Antiviral activity of flavonoids, including quercetin against human and animal viral diseases, has been proven [200]. Its mechanism of action against human herpes viruses was studied and a relationship was found between its antiviral activity and increased intracellular cyclic AMP levels [201]. Its antiviral activity likely occurs at different stages of the virus’s intracellular replication [202].

#### 6.1.3. Application of Chemotherapy in Plant Virus Elimination Processes

During the chemotherapy process of plants, in vitro shoot tips, meristems, nodal segments, shoots, plantlets, bulblets, etc., are cultured on the medium supplemented with chemicals having antiviral activity (Table 5). The antiviral drugs are added to the medium in a wide range of concentrations (up to 100 mg/L), and its level depends on the type of drug, plant species and genotypes, and the type of virus that needs to be eradicated. The duration of treatments is also very various, and treatments often are repeated if necessary; the in vitro cultures are sub-cultured several times on the same medium containing antiviral drugs. Applied methods and their effectiveness have been detailed in several reviews and studies [28,29,203,204].

The most frequently used synthetic nucleoside/nucleotide analogues applied in low concentration hardly damage the plants [162,205], but several researchers reported serious symptoms as a result of treatment by antiviral drugs, which are considered the main limit of method application [204].

However, the ribavirin, which is the most common antiviral drug in experiments with plants, can result in serious growth abnormality, as was observed many times. The phytotoxic effect of antiviral drugs depends on the plant species and cultivars [5,54,135,156,206,207,208] and on the type [86,159,209] and concentration of applied chemicals [5,140,209,210,211]. Moreover, antiviral agents showed sufficient antiviral activity only when used at a concentration at which phytotoxicity effect was also detected [122]. Nevertheless, abnormal growth of plants makes the production of virus free plants difficult [159]. Exposure time also plays a role in the onset of phytotoxicity [140,156,159,212,213,214].

**Figure 1:**
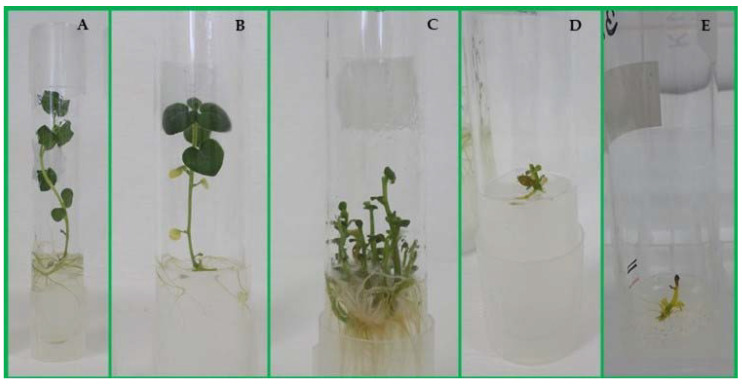
Effect of antiviral drugs on the growth and development of potato (*Solanum tuberosum* L.) in vitro shoots. Control plantlets on medium without antiviral drug (**A**); yellowing bottom leaves on plantlets grown on medium with ribavirin (25 mg/L) (**B**); enhanced shoot proliferation of explants (V-4 clone) grown on medium with 30 mg/L RBV + 20 mg/L ZDV (**C**); microtuber development on nodal explants of V-2 clone cultured on medium with 30 mg/L RBV + 20 mg/L ZDV (**D**); shoot tip necrosis on plantlets of 014PS clone grown on medium with 25 mg/L Adefovir (**E**). Photographs were taken by authors in Nyíregyháza (Hungary)

Phytotoxicity can be expressed by several symptoms (Figure 1). Decreased survival and regeneration rates reported most frequently, followed by inhibition of shoot growth, while root development, hyperhydricity, necrosis, chlorosis and discoloration of green parts, especially leaves, and dwarfing also can observed. Proliferation of shoots is also often affected; however, it can be stimulated many times. After chemo treatment, in vitro cultures are grown on the medium without antiviral chemicals to regenerate; however, they are not always able to recover.

### 6.2. Effect of Antiviral Drugs on Plants

#### 6.2.1. Effect of Ribavirin on Herbaceous Plants

Several pieces of experimental information about the effect of antiviral chemicals on potato (*Solanum tuberosum* L.) could be achieved from literature, and it is not surprising that the most common means of potato virus elimination is chemotherapy [28] (Table 5).

The effect of RBV was studied over a wide range of concentrations (0–200 mg/L) in five potato cultivars (‘Katahdin’, ‘Zhongshu 3’, ‘Chuanyu 5-4’, ‘Favorite’, ‘Saturna’) infected by PVX, PVS, PVA, and PLRV or by co-infected by PVY, PVS, and PVM. The shoot length and the fresh weight of potato plantlets decreased significantly at least by 50.6% and 56.1%, respectively, as RBV concentration increased (from 0 to 150 mg/L) in each group formed according the virus type. The rate of growth inhibition depended on the virus infection: plantlets infected by PLRV tolerated the treatment best, while PVX and PVS infected plantlets were the most sensitive for RBV content in the medium. However, there were no significant differences in growth inhibition rate between potato cultivars; when they were cultured on the medium with 75 mg/L RBV their growth was decreased by about 50% compared to their controls. The majority of plantlets did not survive the culture on the medium with 200 mg/L RBV [211] (Table 5).

Regeneration capacity of potato (*S. tuberosum* L.) cultivars (‘Amelia’, ‘Christian’, ‘Nicoleta’, ‘Roclas’) decreased as RBV concentration increased from 10 to 50 mg/L. Although great differences in regeneration ability exist between cultivars on control medium (the lowest rate was ~31% detected in ‘Amelia’, and the highest rate was found in ‘Nicoleta’ ~75%), at the higher RBV concentrations (˃50 mg/L), each cultivar regenerates shoots under the rate of 20% [206].

Similarly, the regeneration rates of potato (*S. tuberosum* L.) meristems were significantly different on control media, they varied from 33.3% (‘Luiza’) to 96.2% (‘Redsec’), but the regeneration abilities were significantly reduced by 35 mg/L RBV only in ‘Armonia’, ‘Linia 1161′, and ‘Luiza’ (by about 9–10%) [207]. The length of regenerated shoots was significantly decreased in each variety (by 76–79%) [207]. The number of nodes were also significantly reduced in each treated cultivar, but responses of genotypes were more differentiated. The rate of decrease was the least (43%) in ‘Luiza’, while the largest reduction (69%) was observed in ‘Redsec’ [207]. The rooting capacity and the number of roots were also affected by RBV. For example, ‘Luiza’ developed 13.3 roots per explants on control medium, while it did not develop root on medium with RBV at all. In average of cultivars, the number of roots were decreased about 12 times compared to the control. These inhibitor effects could also be detected during the first sub-culture of plantlets, especially in the length of shoots [207].

Shoots of other potato (*S. tuberosum* L.) cultivars (‘Burren’, ‘Binella’) that regenerated on medium containing 30 mg/L RBV showed yellow discoloration and weak growth, although these plants later developed normally on RBV-free medium. The RBV concentration and the size of the isolated meristems affected the length of regenerated shoots. The longest shoots (7.8 and 6.5 cm in ‘Burren’ and ‘Binella’, respectively) developed from meristems in the length of 300 µm on the medium containing the RBV at the lowest (10 mg/L) level. The shortest shoots (1.9 and 1.5 cm for ‘Burren’ and ‘Binella’, respectively) developed from the smallest (100 µm) meristems on the media with 30 mg/L RBV [54].

Regeneration of potato (*S. tuberosum* L.) cultivar ‘Diamand’ was inhibited by 20 mg/L RBV added to all the shoot induction, shoot multiplication, and rooting media. The regeneration rates decreased from 30.0–45.5% (both results observed in control treatments) to 26–33.3%, respectively [121]. Authors found also that application of RBV alone was not effective enough, but it resulted in several growth abnormalities.

To prevent the phytotoxic effect of high dose RBV, application of 12 mg/L RBV in shoot multiplication medium is suggested for virus elimination in potato (*S. tuberosum* L.). Plantlets should be cultured on this medium for four weeks, and repetition of treatment also recommended if necessary [215].

Although the main method for virus elimination in ornamental plants is the meristem culture alone, several results have been reported about application chemotherapy including RBV treatment [29]. The effect of RBV was tested on gerbera (*Gerbera jamesonni* Bolus, ‘Zingaro’) capitulum explants, which were regenerated on media supplemented with RBV in a range of concentration from 10 to 100 mg/L. The regeneration ability of formed calli was significantly reduced (84%, 56%, 36%, and 8%) with increasing RBV level (10, 20, 30, and 50 mg/L, respectively). All explants became brown and died on the medium containing 100 mg/L RBV [216].

The effect of RBV depended on the RBV concentration, the amount of virus in plant, and the plant genotypes during virus elimination process including several tulips (*Tulipa* L.) and narcissus (*Narcissus* L.) genotypes [5]. In the case of tulip, effect of genotypes was significant: the regeneration ability of an old variety (‘D’) was significantly inhibited by 12.5 mg/L RBV (regeneration rate was 69.6% in treated and 133% in control plants), while the regeneration rate of ‘P2’ breeding line was even higher at this concentration. ‘P1’ showed a higher regeneration rate than the control even at 25 mg/L RBV. Application of 50 mg/L RBV for ‘D’ variety has already resulted in significant shoot death. Regeneration ability after RBV treatment (12.5 mg/L RBV for 10 weeks) was lower for clones that were proven to be infected with the virus compared to those that were only ‘suspected of the virus infected’. The most infected ‘D’ old variety had the worst regeneration capacity [5]. All narcissus (*Narcissus* L. ‘Lajkonik’ and ‘0.985T’ clone) plantlets survived each treatment by RBV (12.5, 25, and 50 mg/L), although the highest level of RBV significantly reduced the growth of shoots [5].

#### 6.2.2. Effect of Other Antiviral Chemicals Applied Alone or in Combination on Herbaceous Plants

Besides RBV, Acyclovir, AZA, cytarabine, 5-bromouracil, 2-thiouracil, and ZDV were applied in concentration of 5, 10, 15, 20, 25, and 30 mg/L in the virus elimination experiment of potato (*Solanum tuberosum* L.) plantlets. The rate of the regenerated plantlets decreased as the level of chemicals increased from 5 to 30 mg/L in the case of all tested antiviral agents. The growth of shoots was also inhibited and slowed down by higher concentrations (20, 25, and 30 mg/L). High phytotoxicity was detected in shoots treated by 30 mg/L Acyclovir, AZA, 2-thiouracil, RBV, and ZDV, resulting in a regeneration rate below 30%. Shoots that regenerated on medium with 30 mg/L 2-thiouracil were unable to develop further, and finally they died. However, the 5-bromouracil caused slight phytotoxicity (70.13% regeneration on medium with 30 mg/L 5-bromouracil), but its antiviral activity was also the weakest [209].

To reduce phytotoxicity of RBV, it was applied in combination with Oseltamivir (OSTV) in meristem culture medium for potato (*S. tuberosum* L., ‘Roclas’). Very high regeneration results were obtained (83.3–100% and 60–87.5% for PVX and PVY infected potato plants, respectively) when 40 mg/L OSTV was combined with 20 or 40 mg/L RBV. However, a higher dose of OSTV (80 mg/L) combined with 20 mg/L RBV has already reduced the rate of regeneration of meristems (50–70% and 37.5–71% for PVX and PVY infected plants, respectively) [156].

Effect of the sterilization methods on phytotoxic and antiviral activity of the chemicals (RBV, 5-Azacytidine (4-amino-1-β-d-ribofuranosyl-5-triazin-2[1H]-one, AZA); 3-Deazauridine (4-hydroxi-1-β-Dribofuranosyl-2 [1H] pyridine, DZD)) was tested on single node cuttings of potato (*S. tuberosum* L., ‘Baraka’). Antiviral agents were used after autoclaving together with the medium (120 °C, 1 atm, for 15 min) or after sterilization with the Millipore filter (0.22 µm) in concentration of 20 or 30 mg/L, and single node cuttings were cultured for 62 days on these media. The way of sterilization did not affect significantly the survival rate of explants; they were 55.5% and 37.5% in filtered treatments, while they were 41.7% and 33.3% after sterilization by autoclave, in application of RBV and AZA, respectively. Survivors were found in the same proportion (28.6%) in both filtered and autoclaved DZD treatments. However, the length of shoots was smaller (3.0, 6.0, and 5.0 cm) after filter sterilization of antiviral agents of RBV, AZA, and DZD compared to autoclaved ones (4.0, 9.0, and 8.0 cm, respectively). Regardless, the healthiest shoots were regenerated after RBV treatments independently from the sterilization way [122].

**Figure 2:**
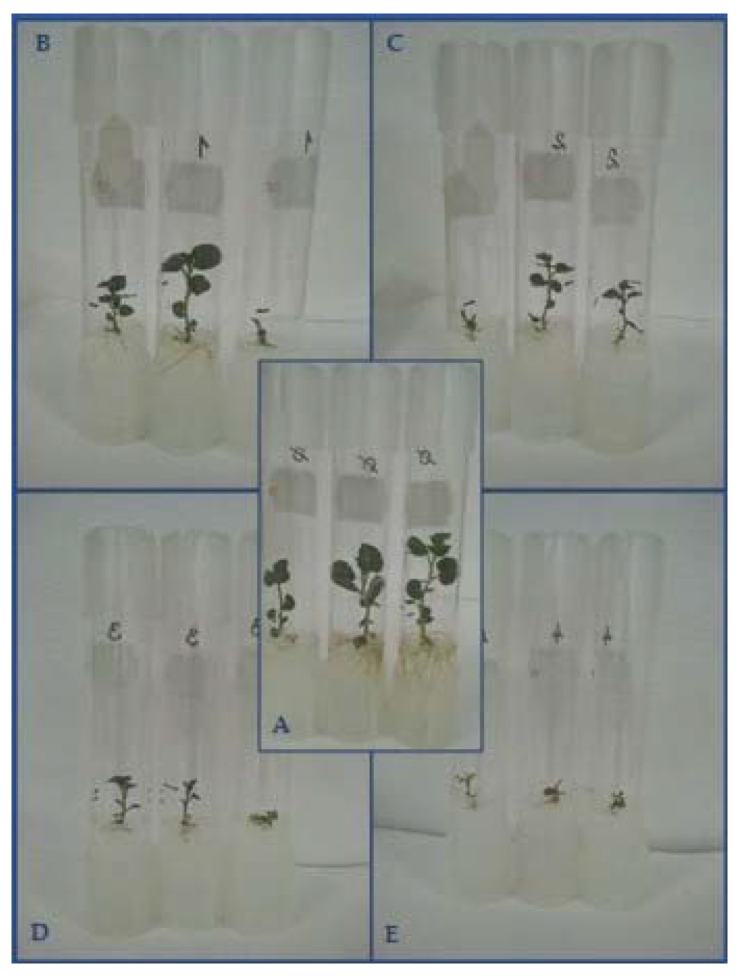
Effect of combined application of RBV and ZDV on V4 potato (*S. tuberosum* L.) in vitro shoot culture. Control plantlets on medium without antiviral drug (**A**); plantlets grown on medium with 10 mg/L RBV + 40 mg/L ZDV (**B**); plantlets grown on medium with 20 mg/L RBV + 30 mg/L ZDV (**C**); plantlets grown on medium with 30 mg/L RBV + 200 mg/L ZDV (**D**); plantlets grown on medium with 40 mg/L RBV + 10 mg/L ZDV (**E**). Photographs were taken by authors in Nyíregyháza (Hungary).

Ribavirin and Zidovudine were applied in combination on eight potato (*S. tuberosum* L.) genotypes (seven breeding line and ‘Jazzy’ cultivar), which were (co)infected by potato virus(es) in different ways. The dose of antiviral agents was determined so that the two active ingredients together did not exceed 50 mg/L; accordingly, increasing levels of RBV (from 0 to 40 mg/L) were combined with decreasing levels of ZDV (from 40 to 0 mg/L). Nodal segments were cultured on the medium with antiviral chemicals for four weeks. In the average of genotypes, the survival rates were decreased from 80.3% to 57.2%, as RBV content increased from 0 to 40 mg/L. However, the responses of genotypes were various. In V2 clone (co-infected by PVM and PVS), only the combination with highest RBV content (40 mg/L RBV + 10 mg/L ZDV) decreased significantly the rate of survival [217]. The shoots of V3 clone (infected by PVM) survived at a low rate (maximum 68.3%) in all treatments, and only the combination 20 mg/L RBV with 30 mg/L ZDV reduced significantly the survival rate. The lowest rates were detected in 1469/83 clone (co-infected by PVM, PVS, and PLRV), and no significantly different results were found in treatments. Survival rates of other breeding lines decreased as RBV level increased, although in general, only the treatments including combination 30 mg/L RBV + 20 mg/L ZDV or 40 mg/L RBV + 10 mg/L ZDV resulted in significant decrease [217] (Figure 2).

However, the main problem was the yellowish discoloration of shoot tips, which occurred in almost all shoots grown on the medium with 40 mg/L RBV + 10 mg/L Zidovudine. Very pale-yellow shoot tips never developed to healthy plantlets after passage onto the medium without antiviral chemicals. When yellowish discoloration occurred only on older leaves, the plantlets survived and were able to regenerate from isolated shoot tips. Another phenomenon with potatoes (S. *tuberosum* L.) is the development of microtubers on treated explants instead of shoot development that makes shoot tip isolation impossible after treatment. On the control medium, only V4 and V1 clones formed microtubers at a 16.7% and 6.6% rate, respectively. The rate of tuberization increased as the RBV content increased in combined treatments; in the average of genotypes, 36.7% was the rate of tuber-forming explants in the case of the majority of tested genotypes (six of eight genotypes). The rate of proliferated shoots also increased from 7.5% to 19.2% as RBV content increased from 0 to 40 mg/L [217].

Our research team tested the phytotoxic and antiviral effect of Adefovir (ADE) in two breeding lines of potato (014PS and 06RF infected by PVS and PVM, respectively) at concentrations of 0, 25, 50, 75, and 100 mg/L. Nodal segments were cultured for four weeks, and they were observed weekly. 014PS clone proved to be sensitive for ADE, because all explants died on the medium containing ADE more than 50 mg/L, while 15% of 06RF shoots survived the culture on the medium with 100 mg/L Adefovir. 014PS explants died at a 80% rate on medium with 50 mg/L ADE after one week culture, while explants of 06RF died gradually on the same medium, resulting in survival rates of 75%, 70%, 65%, and 50% by the end of the first, second, third, and fourth week, respectively. ADE inhibited the shoot growth significantly; even though explants survived, no shoot growth could be detected on medium with ADE above 25 mg/L (014PS) or above 50 mg/L (06RF). The length of shoots was decreased by 38% compared to the control by application of 25 mg/L ADE in 014PS. In the case of 06RF, the shoot lengths were reduced by 52.3% and 80% by using 25 and 50 mg/L ADE, respectively. Development of roots were significantly inhibited on medium containing ADE at 25 mg/L level, root formed only on 20% (014PS) and 45% (06RF) of explants. The number of roots decreased significantly from 9.6 (control) to 3.7 in 014PS clone, and from 3.6 (control) to 2.5 in 06RF. Root did not develop at all on explants grown on media containing more than 25 mg/L ADE. However, shoot tips grown on media containing Adefovir more than 25 mg/L became yellow and did not regenerate shoots at all during post-culture on the medium without antiviral chemicals. Although the survival rates were 85% and 100% in 014PS and 06RF clone, respectively, cultured on medium with 25 mg/L Adefovir, their regeneration rates were only 35% and 25% (unpublished data).

The answer was sought to an interesting question in experiments with tobacco (*Nicotiana occidentalis* ssp. *obliqua* Wheeler), in which the phytotoxic effect of ethylene glycol methylether (EGME) and dimethylsulfoxide (DMSO) at concentrations of 0.25, 0.5, and 1.0 mL/L was tested. EGME and DMSO play a role in antiviral experiments as solvent for antiviral drugs. After two weeks culture, dead spots appeared on the leaves of tobacco plants grown on medium with EGME, and the growth of the plants was also inhibited at a concentration of 1.0 mL/L. After three weeks, symptoms of phytotoxicity were seen in all treatments. No phytotoxicity was observed on the plants when DMSO was used [212]. Glycyrrhizin as antiviral drug was also tested in tobacco (*Nicotiana tabaccum* L.) culture, at concentrations of 0.1–1.0 and 10.0 mM applied for six weeks. At a low level of glycyrrhizin, weak necrotic and dwarfing symptoms appeared, but all plants survived. Larger concentration of glycyrrhizin (1.0 and 10.0 mM) resulted in dwarfism, chlorosis, and necrosis symptoms, and some plants were destroyed [212]. High survival rates (80–100%) were observed in Chinese cabbage (*Brassica pekinensis* (Lour.) Rupr., ‘Manoko’) plantlets cultured on media with 50 mg/L Cidofivir, Adefovir, Tenofovir, PMEDAP, or Ribavirin, when the exposure time was six weeks [159]. However, after nine-week-long treatment, differences in survival rates could be observed, e.g., the highest rate (96.7%) was obtained in Adefovir treated plants, while the lowest rates (63.3%) were detected in Cidofivir and PMEDAP treatments. The majority of plantlets treated by these latter chemicals died (survival rates were below 10%) by the end of 12 weeks, while plantlets treated other chemicals survived at a rate of 53.3–73.3%. Moreover, application of 50 mg/L Cidofovir and PMEDAP decreased the dry weight of plantlets compared to the control and other chemicals, although a significant effect was only proven in Cidofovir treated plants [159]. Older leaves of these treated plantlets became yellow, and survival rates were significantly decreased after 12 weeks (3.3% and 6.7% in Cidofovir and PMEDAP treated plants, respectively [159]). Sugarcane (*Saccharum officinarum* L., ‘CoC 671’,) shoot tips were cultured on medium with RBV or 8-Azaguanine at the level of 5, 10, 25, and 50 ppm. Shoot tips began to grow within some days and reached 2–3 leaf stage in 3–4 weeks. Application of RBV at 5, 10, 25, and 50 ppm level did not affect the regeneration rates of shoot tips (obtained in a range of 80–88%) compared to the control (90%). However, using of 8-Azaguanine at the same levels significantly reduced the regeneration rates down to 44–56% [210].

**Table 5:** Phytotoxic effect of the chemotherapy on herbaceous plants.

Plant Species, Cultivar, Virus	Methods	Survival and/or Regeneration [Reference]
Tobacco, *Nicotiana occidentalis* ssp. *obliqua* Wheeler, ASGV	Plantlets on MS with 30 g/L sucrose, 100 mg/L myo-inositol, 0.4 mg/L Thiamine-HCl, 192 mg/L NaH_2_PO_4_ · 2H_2_O, 80 mg/L adenine sulphate, 2 mg/L ZEA. Antiviral chemicals: DHT: 20 µg/mL for 9–12 wk; RBV: 10 µg/mL, for 12 wk; QRC 10 µg/mL + RBV10 µg/mL: for 9–12 wk; OMB: 10 µg/mL, for 12 wk; GLY 80 µg/mL + QRC 10 µg/mL, for 18 wk. Culture at 22 °C, 16 h L, 42 µmol m^−2^ s^−1^. Sub-culture: 3 wks. Phytotoxicity of the solvents was tested: EGME, DMSO in 0.25, 0.5, 1.0 mL/L.	Phytotoxicity of GLY at 1.0 and 10.0 mM: stunting, chlorosis, necrosis, after 6 wks some plants died; 0.1mM: slight necrosis, some stunting, but all survived. EGME: 2 wks: necrotic spots and reduced growth (1.0 mL/L), 3 wks: all phytotoxic symptoms. DMSO (0.25, 0.5, 1.0 mL/L): vigorous, healthy plants [212]
Sugarcane, *Saccharum officinarum* L., ‘CoC 671’, SCMV	AP meristem (0.5–1.0 mm, 2 LP) on liquid MS with 5% coconut milk, 100 ppm GA_3_. RBV and 8-azaguanine added to media in 5, 10, 25, and 50 ppm. Sub-culture: 5 ds, 3×, then rooting (solid 1/2 MS, but full iron and 1.0 mg/L IBA) and acclimatization.	Regeneration rates on 5/10/25/50 ppm: RBV: 84%; 88%; 82%; 80%. 8-azaguanine: 56%; 52%; 52%; 44%. [210]
Potato, *Solanum tuberosum* L., ‘Baraka’, PVY	Nodal cuttings (1.0 cm) MS with 1 mg/L thiamine, 100 mg/L myo-inositol, 2 mg/L glycine, 30 g/L sucrose; 8.0 g/L agar; 0.001 mg/L NAA, 1.0 mg/L KIN, 0.1 mg/L GA_3_, pH 5.7. Single node cuttings on medium with 20 mg/L RBV, 20 mg/L AZA or 30 mg/L DZD for 60 ds. Chemicals were autoclaved (120 °C, 1 atm, for 15 min) or filtered (0.22 µm). Culture: 25 ± 2 °C, 16 h, 110 µmol m^−2^ s^−1^.	Survival rates/shoot length of filtered chemicals: RBV:55.5%/3.0 cm; AZA: 37.5%/6.0 cm; DZD: 28.6%/5.0 cm. Those of autoclaved chemicals: ARBV: 41.7%/4.0 cm; AZA: 33.3%/9.0 cm; DZD: 28.6%/8.0 cm. [122]
Potato, *Solanum tuberosum* L., ‘Diamond’ PVY	In vitro shoot culture on MS medium with 0.2 GA_3_, 30 g/L sucrose, 25 ± 2 °C, 16 h L, 2500 lux. 20 mg/L RBV was added to the shoot induction, shoot multiplication and rooting media.	Regeneration rate was decreased by RBV from 30–45.5% (control) to 26–33.3% (treated) [121]
Potato, *Solanum tuberosum* L., ‘Amelia’, ‘Christian’, ‘Nicoleta’, ‘Roclas’, PVX, PVY, PVA, PVS, PVM, PLRV	AP meristems (0, 1; 2 or 4 LPs) cultured on PM with 5.7 × 10^−6^ M IAA; 4.9 × 10^−6^ M IBA, 8.6 × 10^−7^ M GA_3_. RBV added to the medium in a range of 10–50 mg/L. Culture for 6, 8, and 10 wks.	Presence of RBV in media decreased the regeneration rates of meristems by 27–56%. [206]
Chinese cabbage, *Brassica pekinensis* (Lour.) Rupr., ‘Manoko’, TYMV	In vitro plantlets from seeds cultured on MS with 2.0 mg/L glycine, 100 mg/L myo-inositol, 0.5 mg/L nicotinic acid, 0.5 mg/L pyridoxine, 0.1 mg/L thiamine, 20 g/L sucrose, at 23 °C, 16 h L, 90 µmol m^−2^ s^−1^. 50 mg/L RBV, (R)-PMPA, PMEA, PMEDAP, (S)-HPMPC was added to the medium when plantlets were 3 weeks old (the liquid medium was exchanged). Cultured for further 4 weeks.	Survival rates after 3/6/9/12 wks: RBV: 96.7/80/76.7/66.7; (S)-HPMPC: 96.7/86.7/63.3/3.3; PMEDAP: 96.7/96.7/63.3/6.7; (R)-PMPA: 100/83.3/70/53.3; PMEA: 100/100/96.7/73.3; control: 93.3/83.3/76.7/73.3. [159]
Potato, *Solanum tuberosum* L., ‘Burren’, ‘Binella’, PVY	Meristem (100–200–300 µm) from in vitro plantlets, cultured on MS with 2 mg/L glycine, 5 mg/L nicotinic acid, 5 mg/L pyridoxine, 5 mg/L thiamine, 5 mg/L ascorbic acid, 200 mg/L myo-inositol, 2 mg/L GA_3_, 0.2 mg/L KIN, 3% sucrose and 0.6% agar. Sub-culture: 20 ds. RBV 10, 20 or 30 mg/L. Culture: 25 ± 2 °C, 16/8 h (L/D), 2.5 µmol m^−2^ s^−1^.	Survival rates: 100/200/300 µm (in the average of cultivars: RBV 10 mg/L: 87.5%/97%/100%; RBV 20 mg/L: 90.5%/95.0%/100%; RBV 30 mg/L: 78.5%/89.5%/95.0%; Control: 87%/97%/100%. Survival rates of ‘Binella’/’Burren’ in the average of meristem lengths: RBV 10 mg/L: 93.7%/96.0%; RBV 20 mg/L: 95.0%/92.0%; RBV 30 mg/L: 87.7%/87.7%; control: 93.3%/96.0%. [54]
Potato, *Solanum tuberosum* L., ‘Armonia’, ‘Linia 1161’, ‘Luiza’, ‘Productiv’, ‘Redsec’, ‘Speranta’, PVX, PVY, PVS, PLRV	Isolated meristems regenerated on MS with 221 mg/L NaH_2_PO_4_ · 2H_2_O, 0.4 mg/L thiamine-HC1, 100 mg/L myo-inositol, 30 g/L sucrose, 7.5 g/L agar, 35 mg/L RBV. Culture at 20–21 °C, 16 h L, 2000 lux. Multiplication on MS medium with 1.0 mg/L IAA, 1.0 mg/L IBA, 0.3 mg/L GA_3_, for 6–8 wks. Virus-free plantlets sub-cultured on MS with 0.1 mg/L IAA, 0.1 mg/L GA_3_, 1.5 mg/L BA.	Regeneration rates of control/treated: ‘Armonia’: 34.4%/24.1%; ‘Linia 1161’: 57.0%/52.3%; ‘Luiza’: 33.3%/30.0%; ‘Productiv’: 64.3%/63.4%; ‘Redsec’: 96.2%/95.6%; ‘Speranta’: 79.2%/79.1%. The shoot lengths of control/treated (cm): ‘Armonia’: 10.7/2.4; ‘Linia 1161’: 10.8/2.4; ‘Luiza’: 8.8/2.1; ‘Productiv’: 12.9/2.97; ‘Redsec’: 14.6/3.1; ‘Speranta’: 11.2/2.6. [207]
Tulip, *Tulipa* L. polish breeding clones (P1–P8), new selections (S1–S8) and old cultivars (A–F), TBV	1st experiment including 3 genotypes: RBV in concentrations of 12.5; 25; and 50 mg/L added to the shoot multiplication medium. Culture for 10 wks, then sub-culture 2× on medium with 12.5 mg/L RBV for 2 months. 2nd experiment: including 7 genotypes: 12.5 mg/L RBV added to the medium for culture of initial explants, culture for 4 months.	1st experiment: number of regenerated shoots: in control/12.5 mg/L/25 mg/L/50 mg/L RBV: ‘D’: 75/39/33/10; ‘P1’: 70/0/57/30; ‘P2’: 73/139/50/37. 2nd experiment: regeneration rates in control/RBV treated: ‘S3’: 74.3%/58.3%; ‘S4’:78.8%/81.1%; ‘S5’: 58.3%/29.1%; ‘P3’: 78.9%/37.1%; ‘P4’: 62.5%/21.7%; ‘E’: 36.9%/34.8%; ‘F’: 70.8%/25.2% [5]
Narcissus, *Narcissus* L., ‘Lajkonik’, and breeding clone ‘0.985T’ NMV, NLV	Bulblets segment on MS with RBV in concentrations of 12.5; 25; and 50 mg/L, for 10 wks.	The shoot length and the fresh weight of plantlets decreased significantly at least by 50.6% and 56.1%, respectively, as RBV concentration increased (from 0 to 150 mg/L) in each infection group. [5]
Potato, *Solanum tuberosum* L., ‘Roclas’, PVX, PVY	Nodal cuttings from in vitro culture on MS with antiviral compounds: V1: 20 mg/L RBV + 40 mg/L OSTV; V2: 40 mg/L RBV + 40 mg/L OSTV; V3: 20 mg/L RBV 20 + 80 mg/L OSTV. Culture: at 20 ± 1 °C, 16 h L. S1: 26 ds; S2: 30 ds; S3: on MS free of antiviral compounds 28 ds. After 7 ds for acclimatization, the plants were sprayed 2× a week with a *Satureja hortensis* essential oils suspension (1/1000, 5 mL each plant). Weekly alternating treatment: H_2_O_2_ (1 mM pH 5.6) and Ascorbic Acid (3 mM), for 45 ds.	Regeneration rates: S1/S2/S3/sprayed/non-sprayed: PVX infected: V1: 83.3%; 83.3%; 88.9%; 100%; 83.3%. V2: 87.5%; 85.7%; 91.7%; 87.5%; 87.5%. V3: 62.5%; 70.0%; 62.5%; 66.7%; 50.0%. PVY infected: V1: 87.5%; 78.6%; 72.2%; 60.0%; 87.5%. V2: 62.5%; 71.4%; 76.2%; 75.0%; 75.0%. V3: 37.5%; 50.0%; 56.2%; 71.4%; 57.1%. [156]
Potato, *Solanum tuberosum* L., PLRV	Nodal segments were cultured on MS with ME, KIN, GA_3_, BA, NAA at different level. Antiviral agents (Acyclovir, AZA, Cytarabine, 5-Bromouracil, RBV, 2-Thiouracil, ZDV) in concentration of 5, 10, 15, 20, 25, and 30 mg/L. Culture for 4 wks, at 25 ± 2 °C, 16 h L, 40 µmol m^−2^ s^−1^, 70% RH.	The regeneration rates decreased as the level of chemicals increased (from 5 mg/L to 30 mg/L): Acyclovir: 77.1–22.9%; AZA: 81.9–20.8%; Cytarabine: 90.96–43%, 5-Bromouracil: 90.3–70.1%, RBV: 76.4–29.2%; 2-Thiouracil: 74.99–10.41%; ZDV: 79.85–16.7%. The best control regeneration rate (90.97%) on medium with 0.1 mg/L GA_3_, 0.1 mg/L NAA, 500 mg/L ME. [209]
Garlic, *Allium sativum* L., ‘N9A’, ‘Anton’, ‘Tristan’, French ‘D’Alsace Freres’ ‘Mako’, GCLV	ST (1.0 mm) or meristems (2 LPs) from 14-day-old culture grown on MS medium with 0.5 mg/L BA, 0.1 mg/L NAA, 0.5 mg/L GA_3_, 1 mL/L antibiotic ProClin, and supplemented with 25 mg/L or 50 mg/L RBV. Culture: 21 ± 1 °C, 16 h L, 20–25 μmol m^−2^ s^−1^ for 6 wks.	Survival rate was 72%. [218]
Gerbera, *Gerbera jamesonni* Bolus, ‘Zingaro’, CMV	Capitulum explants on MS with 0.25 mg/L IAA, 0.5 mg/L TDZ, supplemented with 10, 20, 30, 50, 100 mg/L RBV. Cultured at 25 ± 2 °C, 16 h L, 50 mol m^−2^ s^−1^ for 45 ds. Sub-culture on the medium without RBV, then rooting.	Regeneration rates of explants on 10 mg/L RBV: 84%; 20 mg/L RBV: 56%; 30 mg/L RBV: 36%; 50 mg/L RBV: 8%; 100 mg/L RBV: all explants browned and died. [216]

ACY: acyclovir (acyclic purine nucleoside analogue); AP: apical; ASGV: Apple stem grooving virus; AZA: 5-Azacytidine (4-amino-1-β-d-ribofuranosyl-5-triazin-2[1H]-one); BA: 6-benzyladenine; CMV: Cucumber mosaic virus; d(s): day(s); D: darkness; DHT: 2,4-dioxohexahydro- 1,3,5-triazin; DMSO: dimethylsulfoxide; DZD: 3-Deazauridine (4-hydroxi-1-β-d-ribofuranosyl-2 [1H] pyridone); EGME ethylene glycol methylether; GA_3_: gibberellic acid; GCLV: Garlic common latent virus; GLY: glycyrrhizin; IAA: β-indolyl-acetic acid; IBA: Indole-3-butyric acid; KIN: kinetin; L: light; LP(s): leaf primordium(a); ME: malt extract; MS: MS medium, [62]; NAA: α- naphthalene acetic acid; NLV: Narcissus latent carlavirus; NMV: Narcissus mosaic potexvirus; OMB: ombuin (7,4′-dimethyl quercetin); OSTV: oseltamivir ([(3*R*,4*R*,5*S*)-4-acetamido-5-amino-3-(1-ethylpropoxy)-1-cyclohexane-1-carboxylic acid)]); PDV: Prune dwarf virus; PLRV: Potato leaf roll virus; PM: PM medium: [219]; PMEA: 9-[2-(phosphonomethoxy)ethyl]adenine, adefovir; PMEDAP: 2,6-diamino-9-[2-(phosphonomethoxy)ethyl]purine; PVA: Potato virus A; PVM: Potato virus M; PVS: Potato virus S; PVX: Potato virus X; PVY: Potato virus Y; QRC: Quercetin (3,3′,4′,5,7-pentahydroxyflavone); RBV: Ribavirin (1-β-d-ribofuranosyl-1, 2,4 triazone-3-carboxamide) Virazole^®^ a synthetic broad-spectrum antiviral nucleoside; RH: relative humidity; (R)-PMPA: 9-[(R)-2-(phosphonomethoxy)propyl]adenine, tenofovir; S1: sub-culture 1; SCMV: Sugarcane mosaic potyvirus; (S)-HPMPC: 1-[(S)-3-hydroxy-2-(phosphonomethoxy)propyl]cytosine, cidofovir, Vistide^®^; ST: shoot tip; TBV: Tulip breaking potyvirus; TDZ: thidiazuron; wk(s): week(s); TYMV: Turnip yellow mosaic virus; ZDV: Zidovudine (azidothymidine; 3′-azido-3′-deoxythymidine); ZEA: zeatin.

#### 6.2.3. Effect of Ribavirin on Woody Plants

Chemotherapy has been also often applied on woody plants, and among them the grapevine (*Vitis* L. species) was treated the most frequently by antiviral chemicals [28] (Table 6). No phytotoxicity was detected on grapevine (*Vitis champanii* Planch) plantlets during the first 20 days of treatment by RBV (15 or 25 mg/L), and the growth of plantlets was similar to the growth of control ones. However, after 30 days of treatment, the edges of the leaves in some plantlets began to turn yellow or brown at both concentrations of RBV. By this time, there were already differences in shoot height: the treated plantlets were significantly lower (1.9 and 1.85 cm in 15 and 25 mg/L RBV treatment, respectively) than control plantlets (2.14 cm) [127].

The survival rates varied from 40% to 53.3% in grapevine (*Vitis vinifera* L.) cultivars (‘Aberkane’: 47.1%; ‘Bezzoul El K’.: 40.0%., ‘Muscat de F’.: 53.3%, ‘Ferrana’: 47.1%) during virus eradication made by 20 mg/L RBV. Moreover, a significant increase was found in the number of new shoots compared to the control [220].

Similarly, the multiplication rate of grapevine (*Vitis vinifera* L., ‘Servant’) apex and axillary buds gradually increased during the sub-cultures on RBV-free medium after treatment by 80 µmol/L RBV. Hyperhydricity occurred on treated plantlets, but its rate decreased gradually during post-cultures on RBV-free medium, and no mortality was found [203].

Although thermotherapy is the most common method for virus eradication in fruit trees, several reports about their chemotherapy can be found [204]. Some species belonging to the Rosaceae family, including apple, pear, and stone fruits, were treated by RBV.

All apple (*Malus domestica* Borkh, ‘Xinhongjiangjun’) shoots survived the treatment by RBV at 15 and 25 mg/L concentration, which lasted for 60 days. The growth of shoots was similar to the control shoots, although after 60 days some leaves became yellow and brown, which also could be observed in control cultures, and it was maybe due to the natural senescence. However, the proliferation rate was inhibited on treated explants, and the number of new shoots was significantly lower (3.8 new shoots per explant) in cultures treated by 25 mg/L RBV compared to the control (5.5 new shoots per explant) by the 40th day of treatment [140].

RBV was applied on sand pear (*Pyrus pyrifolia*, ‘Jinshui no. 2’) shoot cultures at similar levels (15, 20, and 25 mg/L), and each shoot survived [4]. Meanwhile, it was found that treatment with RBV in concentration of 15 and 25 mg/L for a period of 5–30 days could enhance the growth of in vitro pear plantlets. The growth speed of plants treated under those conditions was significantly higher than that of the control. However, when the treatment period lasted for 35–40 days, the height of treated shoots did not differ significantly from that of the control shoots. RBV also significantly improved the proliferation of in vitro pear shoots, and the number of newly developed shoots on medium containing 25 mg/L RBV was almost two-fold more than that of those on the control medium when treatment lasted for 25–40 days [4].

Application of 20 mg/L RBV for virus elimination of pear (*Pyrus communis* L.) did not induce phytotoxic symptoms on shoot apices explant of ‘Astra’ and ‘Erika’ varieties; they were vigorous, healthy, and green, and each of them survived and regenerated shoots during sub-culture. No adverse effects (neither morphological changes nor abnormalities) were observed on the transplanted plants treated previously with RBV; normal leaves and shoots developed. However, in the case of ‘David’ cultivar, growth was inhibited by RBV, and shoot apex necrosis appeared at about a 30% rate, which died on the regeneration medium [208].

Survival rates of myrobalan (*Prunus cerasifera* var. *divaricata* Borgh), plum (*Prunus domestica* L., ‘Empress’), and sweet cherry (*Cerasus avium* L., Moench., ‘Early Rivers’) were not affected significantly by 10 and 25 mg/L RBV added to the medium, and all of the shoots survived in rates above 80%. Higher concentrations (50 and 100 mg/L) of RBV were proven to be phytotoxic in plum cultures (*Prunus domestica* L., ‘Empress’) and resulted in low survival rates 33.3% and 6.7%, respectively [135].

The multiplication rates of plum explants (*Prunus domestica* L., ‘Magna Lauca’) cut from in vitro shoots treated by RBV in concentrations of 10, 20, 30, 40, and 50 mg/L were significantly reduced (2.0, 2.4, 2.3, 1.5, and 2.2 shoots developed per explant, respectively) compared to the control (4.6 shoots/explant), as it was observed at four weeks after treatment. However, during the second sub-culture, significantly more shoots (11.6, 10, and 9.6 shoots per explant) developed on shoots treated previously by 10, 20, or 30 mg/L RBV, respectively, compared to the control (6.3 shoots per explant) [214]. The same trends were observed for the other tested variety (‘Cacanska Rana’), but the differences were not significant [214].

Low RBV concentrations (5 or 10 mg/L) were used in antiviral experiments with plum (*Prunus domestica* L., ‘Bluefree’) and apricot (*Prunus armeniaca* L., ‘Hanita’) to reduce the phytotoxic effect. Since the successful virus elimination required a longer period of treatment (nine weeks for apricot and 12 weeks for plum), the treatments were applied for various and relatively long periods of time (up to 27 weeks). Even though several sub-cultures were performed on medium with RBV, no phytotoxicity was observed on plantlets, and no significant effects were found on multiplication rate, shoot growth, or rooting [221].

In order to mitigate the adverse effect of antiviral chemicals, a very low RBV concentration (1 mg/L) was tested, and it was found that it can be effective against PPV in apricot (*Prunus armeniaca* L.) in vitro cultures treated for four weeks, although it can depend on the host genotype, the concentration, and type of virus [86].

Rosa (*Rosa hybrida*), also belonging to the Rosaceae family, is an ornamental plant, which is distributed widely and also can be infected by viral disease of *Prunus* sp. and apple [213]. During the 20-day-long treatment, the presence of RBV in the medium increased the regeneration rate (from 34% to that of 82% observed in control) in rosa (*Rosa hybrida*) plants infected by Arabis mosaic virus (ArMV). Increasing the RBV concentration (from 10 to 30 mg/L) significantly increased the regeneration rate from 82% to 94%. However, extending the duration of treatment from 20 to 40 days significantly reduced the regeneration ability of explants in all RBV concentrations at least by 12%, and the greatest inhibitory effect (reduction by 45%) was detected in the case of 30 mg/L RBV. Similar results were observed in Prunus necrotic ringspot virus (PNRSV) infected plants, but application of 30 mg/L RBV for 40 days did not reduce significantly the regeneration rate (88%) compared to those treated for 20 days (92%) [213].

#### 6.2.4. Effect of Other Antiviral Chemicals Applied Alone or in Combination on Woody Plants

No mortality was found in grapevine (*Vitis vinifera* L., ‘Tămâioasă românească’) when shoot cultures were grown on media with different combination of RBV and OSTV concentrations during three consecutive sub-cultures [191]. However, the multiplication rate of ‘Tămâioasă românească’ was decreased compared to the control in each treatment and in all sub-cultures. The high RBV concentration in the combined treatment (40 mg/L RBV + 40 mg/L OSTV) resulted in a significantly lower multiplication rate compared to the control (2.2 and 3.0 new shoots/explant, respectively) during the first sub-culture, while the same treatment caused increased multiplication rates in ‘Burgund’ 63 Mn, halving the RBV to 20.0 mg/L, supplemented by 40 mg/L OSTV in the other treatment, allowing vigorous bud proliferation [191]. Regardless, the multiplication rates decreased during and after the second sub-culture in each treatment and in both cultivars [191].

Other grapevine (*Vitis vinifera* L.) cultivars, e.g., ‘Fetească neagră 7Od’ and ‘Frâncuşă 15Od’ were also tested with the same combination of RBV and OSTV and by the same method. Cultivars responded differently to the treatments. ‘Fetească neagră 7Od’ tolerated better the antiviral chemicals; there was no significant effect on its rate of multiplication during the first and second sub-cultures. Only the combination of 20 mg/L RBV and 80 mg/L OSTV reduced the multiplication rate in the third sub-culture. The multiplication rate of the other variety (‘Frâncuşă 15Od’) was not also influenced significantly during the first sub-culture, while it was significantly reduced by each treatment in the third sub-culture. In the second sub-culture, the shoot proliferation was significantly inhibited by two combinations (40 mg/L RBV with 40 mg/L OSTV and 20 mg RBV with 40 mg/L OSTV) [190].

A very low concentration (1.0 mg/L) of RBV, QRC, and 8-azaguanine was used in virus elimination experiments. They were added to the multiplication medium of apricot (*Prunus armeniaca* L.), and shoot tips were cultured on them for four weeks. Very high survival rates (80% and 100%) could be achieved by RBV and QRC, while no shoot tip survived on the medium with 8-azaguanine [86].

Zidovudine (ZDV) in 25 and 50 mg/L concentration was used for virus elimination on peach (*Prunus persica* (L.) Batsch., ‘Red haven’) in vitro shoots. Although very high antiviral activity of ZDV was found (100% virus-free plantlets), no damage could be detected on plantlets [222].

The application of rimantadine (RMT) and acyclovir (ACY) was tested in experiments including other peach cultivar (*Prunus persica* (L) Batsch, ‘Redhaven’, ‘Suncrest’). Both antiviral drugs were proven to be very effective in concentrations of 25 or 50 mg/L, although they did not induce any kind of phytotoxic symptoms [223].

The tolerance of red raspberry (*Rubus idaeus* L. ‘Babje Leto 2’) to RBV (30 mg/L) treatment was different (survival rates ranged from 60 to 83%) in the plantlets collected from a different mother plant. However, very high survival rates (98–100%) were observed in plants treated by 25 mg/L Azacytidine (AZA) and 25 mg/L Dicyandiamide (synonym: cyanoguanidine) (DCA). However, only the latter chemicals resulted in virus-free plantlets [224].

Nodal cuttings of cassava (*Manihot esculenta* Crantz, Tanzanian landrace) were grown on the medium with salicylic acid (SA) at levels of 0, 10, 20, 30, or 40 mg/L, or RBV in concentration of 0, 5, 10, 15, and 20 mg/L for three weeks. The rate of survival decreased as the level of RBV and SA increased. While 100% of explants survived in control treatment, only 58.3% and 66.7% survivors were found in treatment with 10 mg/L RBV and SA for 42 days, respectively. All explants died on the medium with 40 mg/L SA, and less than half of plants (41.6%) survived the RBV at 20 mg/L level. In the latter case, the phytotoxicity of RBV could be detected as necrosis and chlorosis, leading to defoliation. Similar symptoms were also observed on plantlets cultured on media with 30 and 40 mg/L SA for 42 days. Normal plantlets developed on media containing RBV up to 10 mg/L concentrations. The multiplication rate was reduced from 3.0 shoots per explants observed in control to 2.5–1.3 (RBV treatments) and 1.8–0.6 (SA treatments) [128].

**Table 6:** Phytotoxic effect of the chemotherapy on woody plants.

Plant Species, Cultivar, Virus	Methods	Survival and/or Regeneration [Reference]
Myrobalan, *cerasifera* var. *divaricata* Borgh, ACLSV, PNRSV, Plum, *Prunus domestica* L., ‘Empress’, PNRSV Sweet cherry, *Cerasus avium* L., Moench., ‘Early Rivers’, PDV	ST (1.0 cm) grown on MS with 5.0 µmol/L BA, 0.5 µmol/L IBA, and RBV in concentrations of 10, 25, 50, or 100 mg/L. Culture 24/21 °C, 16 h L, 2000 lux, for 4 wks. Then grown on RBV-free medium, and survivors rooted on medium (2 mg/L IBA). Potted and kept in greenhouse.	Survival rates in control/10/25/50/100 mg/L RBV: myrobalan: 100%/90%/86.6%/86.6%/70%; ‘Empress’: 100%/86.6%/80%/33.3%/26.7%; ‘Early Rivers’: 100%/100%/100%/88%/56%. [135]
Red raspberry, *Rubus idaeus* L., ‘Babje Leto 2’, RBDV	Modified MS with ¼ of nitrates, double Fe salts, 170 mg/L KH_2_PO_4_, 0.4 mg/L thiamine-HCl, 1.0 mg/L BA, 0.05 mg/L IBA, 0.1 mg/L GA_3_. RBV 30 mg/L, AZA 25 mg/L, DCA 25 mg/L added to the media. Culture for 25 ds.	Only RBV showed phytotoxicity: Survival rates were 60%, 70%, and 83% in lines from different mother plants. Survival rates were 98–100% in AZA and DCA treated plants. AZA and DCA did not result in any damage on plants. [224]
Apple, *Malus domestica* Borkh, ASGV	In vitro cultures grown on medium with QRC and RBV (10 mg/L) for 9–12 wks, then sub-culture on medium free of antiviral chemicals.	Trees from treated cultures grown normally and any abnormalities could not be detected. [225]
Plum, *Prunus domestica* L., ‘Bluefree’, Apricot, *Prunus armeniaca* L., ‘Hanita’, PPV	2-month-old plantlets to MS medium with RBV (5 or 10 mg/L) for 9, 12, 16, 20, and 27 wks, sub-culture every 4 wks on the same medium with RBV.	No phytotoxicity was detected. [221]
Grapevine, *Vitis vinifera* L., ‘Servant’, GVA, GLRaV-1	ST (0.2–0.3 cm) and AX buds cultured on MS with RBV 80 μmol/L for 30–60–90 ds, adventitious buds formed on treated shoots post-cultured on MS without RBV for 1–3 cycles. Growing conditions: 24 ± 1 °C, 16 h L, 3000 lux.	The multiplication rates increased after treatment during sub-culture on RBV free medium. No mortality was found, hyperhydricity occurred. [203]
Pear, *Pyrus communis* L., ‘Alexander Lucas’, ‘Bohemica’, ‘Elektra’, ‘Rote Williams’, ASPV	Shoots (5–10 mm) on MS with 1.5 mg/L BA, and RBV 20 mg/L. Culture at 22 ± 1 °C, 16 h L, 60 µmol m^−2^ s ^1^, for 4 wks. Then shoot tip isolation (3 mm) and regeneration on MS with BA 1.5 mg/L.	Survival rate/regeneration rates: ‘Alexander Lucas’: 100%/95%; ‘Bohemica’: 100%/100%; ‘Elektra’: 100%/100%; ‘Rote Williams’: 100%/95%. [226]
Sand pear, *Pyrus pyrifolia*, Burm. ‘Jinshui no. 2’, ACLSV, ASGV	In vitro cultures on MS with 1.0 mg/L BA, 0.2 mg/L IBA, 30 g/L sucrose, 5.3 g/L agar. Sub-culture: 30 d. RBV added to medium at 15, 20, and 25 µg/mL. Culture at 24 ± 1 °C, 16 h, 40 µmol m^−2^ s^−1^, for 40 ds. Then meristem (1.0–0.5 mm) from 5 main shoots and axillary shoots were cultured on MS. 2 cycles of sub-culture.	Survival rate: 100% in each treatment. Regeneration rates in average of different meristem sizes: control: 88.3%; 15 mg/L RBV: 86.2%; 20 mg/L RBV: 62.9%; 25 mg/L RBV: 78.3%. [4]
Pear, *Pyrus communis* L., ‘Astra’, ‘David’, ‘Erika’, ASPV	Shoot apices on MS with 1.5 mg/L BA, 20.0 mg/L RBV. Culture at 22 ± 1 °C, 16 h L, 40 µmol m^−2^ s^−1^ for 4 wks. Then shoot tip isolation for regeneration on MS with 1.5 mg/L BA.	Survival rates were 100% in ‘Astra’, 70% in ‘David’, 100% in ‘Erika’. All survived shoots regenerated shoots. [208]
Grapevine, *Vitis vinifera* L., ‘Tămâioasă românească’, ‘Burgund’ 63 Mn, GFkV, GVA	Single node segments cultured on MS with 1.0 mg/L BA, 0.5 mg/L IAA. Culture: 22 ± 1 °C, 16 h L., 3000–3500 lux. Combination of RBV and OSTV was added to the medium in concentrations of 40 + 40 mg/L (V1); 20 + 40 mg/L (V2); and 20 + 80 mg/L (V3), respectively, for 3 subsequent sub-cultures (S1, S2, S3).	Multiplication rate of ‘Tămâioasă românească’ was decreased in V1 treatment (2.2 shoots/explant) compared to the control (3 shoots/explant). Multiplication rate of ‘Burgund’ 63 Mn increased during S1, later decreased (data n.a.). [191]
Apple, *Malus domestica* Borkh, ‘Xinhongjiangjun’, ACLSV, ASGV, ASPV	In vitro plantlets on MS with 1.0 mg/L BA, 0.1 mg/L NAA, 30 g/L sucrose, 5.6 g/L agar. Sub-culture: 60 ds. RBV added to the medium in 15 or 25 mg/L. Culture: 24 ± 1 °C, 16 h L., 2000 lux, for 60 ds.	Survival rate was 100% in each treatment. Survival rate: 15/25 mg/L RBV: AP meristems: 76.7%/76.7%; AX meristems: 67.7%/88.5%. Proliferation rates (shoots/explant) after 20/40/60 days: control: 1.0/5.5/5.5; 15 mg/L RBV: 1.0/4.8/4.8; 25 mg/L RBV: 1.0/3.8/5.5. [140]
Peach, *Prunus persica* (L.) Batsch, ‘Red Haven’, PDV, PPV, PNRSV	Nodal segments cultured on QL medium for some wks, 21 ± 1 °C, 16 h L., 20.25 μmol m^−2^s^−1^, then chemotherapy: ZDV: 0 mg/L (C0), 25 mg/L (Z25) or 50 mg/L (Z50), RBV: 50 mg/L (C1). Sub-culture on QL with 0.4 mg/L BA, 0.01 mg/L NAA.	Zidovudine 25 or 50 mg/L: no damaged plants [222]
Apricot, *Prunus armeniaca* L., PPV	ST cultured on MS medium with 2 mg/L BA, 0.5 mg/L GA_3_, 1.0 mg/L RBV, 8-azaguanine or QRC. Culture: 25 ± 2 °C, 16 h L, 36 μmol m^−2^ s^−1^, for 30 ds.	Survival rates: 8-azaguanine: 0%; RBV: 80%; QRC: 100%. [86]
Rose, *Rosa* (L.) *hybrida*, PNRSV, ArMV	Nodal cuts on MS with 0.4 mg/L NAA, 0.4 mg/L BA. RBV added to the medium in concentration of 10, 20, and 30 mg/L. Culture for 20 or 40 ds. Then rooting of ½ MS with 6.0 mg/L IAA.	Regeneration rates of ArMV infected plants in 20/40-day-long treatment and the length of shoots (mm): control: 34%/21% (18.7/19.1); 10 mg/L: 82%/64% (21.6/22.6); 20 mg/L: 88%/78% (27.8/85.1); 30 mg/L: 94%/42% (91.5/14.4). Similar results were obtained for PNRSV infected plants. [213]
Peach, *Prunus persica* (L) Batsch, ‘Redhaven’: PPV, Suncrest’: PDV, PNRSV	2-week-old shoot on MS with 0.5 mg/L BA, 0.01 mg/L NAA, 0.5 mg/L GA_3_, supplemented with ACY or RMT at concentration of 25 or 50 mg/L. Culture at 21 ± 1 °C, 16 h L, 22 μmol m^−2^ s^−1^ for 3 wks. Post-culture: on QL medium with 0.5 mg/L BA, 0.01 mg/L NAA.	No phytotoxicity could be observed both in the health and in the vitality of shoots. [223]
Grapevine, *Vitis vinifera* L., ‘Fetească neagră 7Od’, ‘Frâncuşă 15Od’, GLRaV-1 GFLV	Apices and axillary buds cultured on MS with 1 mg/L BA, 0.5 mg/L IAA. RBV and OSTV were added to the medium in combinations: V1 = 40 mg/L RBV + 40 mg/L OSTV; V2 = 20 mg/L RBV +40 mg/L OSTV; V3 = 20 mg/L RBV + 80 mg/L OSTV. Cultured at 22 ± 1 °C, 16 h L, 3000–3500 lux. Sub-cultured on medium with the same antiviral chemicals 3×: S1, S2, S3: 35–40 ds each, then one sub-culture on medium without antiviral chemicals followed by rooting.	Multiplication rates of ‘Frâncuşă 15Od’ in S1: phytotoxicity effect not detected; in S2: the V1 treatment significantly reduced the MR; in S3: each treatment significantly reduced the MR ‘Fetească neagră 7Od’: more tolerant variety, onlyV3 treatment reduced the MR in S3. [190]
Cassava, *Manihot esculenta* Crantz, Tanzanian landrace, EACMVs	In vitro shoot culture: MS with 20 g/L sucrose, 3 g/L agar, 28 °C, 16 h, sub-culture 5 wk. Nodal cuttings were grown on MS with salicylic acid (0, 10, 20, 30 or 40 mg/L), or RBV (0, 5, 10, 15, and 20 mg/L) for 3 wks. Culture at 28 °C, 16 h L. Post-culture on medium free of antiviral compounds	Survival rates after 42 days: RBV: 0 mg/L: 100%; 5 mg/L: 83.3%; 10 mg/L: 72.9%; 15 mg/L: 58.3%; 20 mg/L: 41.6%. SA: 0 mg/L: 100%; 10 mg/L: 66.7%; 20 mg/L: 28.3%; 30 mg/L: 18.8%; 40 mg/L: 0%. [128]
Plum, *Prunus domestica* L., ‘Magna Glauca’, ‘Cacanska Rana’, PNRSV, ACLSV	In vitro shoots (0.8 ± 0.2 cm) grown on MS with 0.75 mg/L BA, 0.14 mg/L IBA, 30 g/L sucrose, RBV in concentration of 10, 20, 30, 40 or 50 mg/L. Culture at 21 ± 2 °C, 16 h L, 50 µmol m^−2^ s^−1^, for 2 wks. Then isolated meristems (0.2 cm) sub-cultivated on MS, monthly.	Multiplication rates (number of new shoots/explant) at 4 wks/8 wks after treatment. ‘Magna Glauca’: control: 4.6/6.3; 10 mg/L RBV: 2.0/11.6; 20 mg/L RBV: 2.4/10; 30 mg/L RBV: 2.3/9.6; 40 mg/L RBV: 1.5/6.7; 50 mg/L RBV: 2.2/7.2. ‘Cacanska Rana’: control: 4.1/4.3; 10 mg/L RBV: 1.2/6.2; 20 mg/L RBV: 2.4/4.5; 30 mg/L RBV: 1.7/5.8; 40 mg/L RBV: 0.9/6.1; 50 mg/L RBV: 1.3/4.9. [214]
Grapevine, *Vitis champinii* Planch, GLRaV-3	In vitro plantlets grown on ½ MS with 15 or 25 mg/L RBV. Culture at 24 °C, 16 h L., 2000 lux, for 40 ds.	Survival rate was 100%. Regeneration rate on medium with 15/25 mg/L: 52.8%/40.0%, control: 53.3%. Length of shoots after 30 day: control: 2.14 cm, treated: 1.9 cm/1.85 cm. [127]
Grapevine, *Vitis vinifera* L., ‘Aberkane’ ‘Bezzoul El Khadem’, ‘Muscat de Fandouk’, ‘Ferrana’, GLRaV-3, GFLV	ST cultured on modified MS with RBV 20 mL/L, for 8 wks, at 24 ± 1 °C, 16 h L. 1–3 sub-cultures on MS without RBV, then rooting.	Survival rates were in ‘Aberkane’: 47.1%; in ‘Bezzoul El K’.: 40.0%., in ‘Muscat de F’.: 53.3%, ‘Ferrana’: 47.1%. [220]

ACLSV: Apple chlorotic leaf spot virus; ACY: acyclovir (acyclic purine nucleoside analogue); AP: apical; ArMV: Arabis mosaic virus; ASGV: Apple stem grooving virus; ASPV: Apple stem pitting virus; AX: axillary; AZA: 5-Azacytidine (4-amino-1-β-d-ribofuranosyl-5-triazin-2[1H]-one); BA: 6-benzyladenine; d(s): day(s); D: darkness; DCA: dicyandiamide; EACMV: East African cassava mosaic virus; GA_3_: gibberellic acid; GFkV: Grapevine fleck virus; GFLV: Grapevine fanleaf virus; GLRaV-1: Grapevine leafroll associated virus serotype 1; GLRaV-3: Grapevine leafroll-associated virus serotype 3; GVA: Grapevine virus A; IAA: β-indolyl-acetic acid; IBA: Indole-3-butyric acid; L: light; LP(s): leaf primordium(a); MR: multiplication rate; MS: MS medium, [62]; NAA: α-naphthalene acetic acid; OSTV: oseltamivir ([(3*R*,4*R*,5*S*)-4-acetamido-5-amino-3-(1-ethylpropoxy)-1-cyclohexane-1-carboxylic acid)]); PDV: Prune dwarf virus; PNRSV: Prunus necrotic ringspot virus; PPV: Plum pox virus; QL: QL medium, [227]; QRC: Quercetin (3,3′,4′,5,7-pentahydroxyflavone); RBDV: Raspberry bushy dwarf (idaeo)virus; RBV: Ribavirin (1-β-d-ribofuranosyl-1, 2,4 triazone-3-carboxamide) Virazole^®^ a synthetic broad-spectrum antiviral nucleoside; RMT: rimantadine (1-(1-Adamantyl)ethylamine hydrochloride), an amantadine analogue; (R)-PMPA: 9-[(R)-2-(phosphonomethoxy)propyl]adenine, tenofovir; S1: sub-culture 1; SA: salicylic acid; ST: shoot tip; wk(s): week(s); ZDV: Zidovudine (azidothymidine; 3′-azido-3′-deoxythymidine).

### 6.3. Suggestions for Decreasing of Phytotoxic Effect of Antiviral Drugs on Plants

All chemicals added to the medium in excessive amount can results in a stress effect in plants. However, the toxic level largely depends on the plant species and within the species on cultivars as well. Concentration of antiviral drugs can be decreased down to a very low level to decrease the toxicity; however, repetition of treatments (consequently sub-cultures on the medium with antiviral drugs) might be necessary. Preliminary experiments to determine the optimal concentration for treatment of a genotype are required, but the sensitivity of the infected plants to chemicals is affected also by the type and by the amount of virus. Although RBV is the most common antiviral drug in plant virus elimination, several other antiviral chemicals can be applied in order to reduce phytotoxicity and increase efficacy. However, it is impossible to recommend antiviral drugs in general due to very different and contradictory responses of genotypes, as cited Špak et al. at the beginning of this section. Moreover, sometimes we have to face the fact that RBV is the gentlest for plants. To find an effective chemical, its optimal concentration, the exposure time, and the number of repeated sub-cultures is a serious task. Combination of RBV with another antiviral drug can be very effective considering both their phytotoxic effects and antiviral activity.

## 7. Virus Elimination by Combined Treatment

Several viruses were proven to be difficult to eradicate from plants by treatments applied alone; thus, several attempts have been made to eliminate them by combined treatments. Thermotherapy and chemotherapy are most frequently used in combination; they can be applied simultaneously or sequentially. Electrotherapy and cryotherapy also can be involved in combined treatments [81,204].

### 7.1. Thermotherapy Combined with Chemotherapy

When RBV was combined with thermotherapy simultaneously, the effect of combined treatments (15 or 25 mg/L RBV and 34 ± 0.5, or 36 ± 0.5, or 38 ± 0.5 °C) on shoots of apple (*Malus domestica* Borkh, ‘Xinhongjiangjun’) was proven to be mainly due to the effect of the heat treatment. Almost all shoots survived (90–100%) the treatment at 34 ± 0.5 and 36 ± 0.5 °C, while the best survival rate was only 46.7% in treatment, in which 38 ± 0.5 °C was applied. Moreover, no stress symptoms were found on shoots cultured at 34 ± 0.5 °C, while those grown at 36 ± 0.5 °C showed chlorosis and rolling. However, serious damages were observed on shoots incubated at 38 ± 0.5 °C, including dark discoloration before dying. The survival rates of treated shoots and of isolated meristems was highly affected by temperature, but it was not dependent on the RBV level (Table 7). Even though 40% and 46.7% of shoots were treated by 38 ± 0.5 °C, the isolated meristems were hardly or not able to regenerate shoots (16.7% was the best result, which was obtained by apical meristem isolated from shoots grown on medium with 15 mg/L RBV) [140].

In the case of sand pear (*Pyrus pyrifolia*, Burm. ‘Jinshui no. 2’), shoots were treated by 15, 20, and 25 mg/L RBV and cultured at 35 °C for up to 40 days, and all shoots survived. The regeneration rates of isolated meristems (1.0 mm in length) decreased (90.9%, 83.3% and 70.6%) as exposure time of heat treatment increased (30, 35, and 40 days, respectively). However, when 0.5 mm meristems were isolated and cultured, this kind of relationship could not be detected (83.3%, 75%, and 85.7% in treatment for 30, 35, and 40 days, respectively). Moreover, the regeneration rates of meristems isolated from the shoots treated by heat for 40 days, decreased from 81.8% to 66.7% when RBV concentration increased from 15 to 20 mg/L in the case of meristems in length of 1.0 mm. Further increase in RBV content to 25 mg/L did not result in further decrease (70.6%). Regeneration rates of 0.5 mm sized isolated meristems were not affected by level of RBV (86.7%, 92.9%, and 85.7% in treatment by 15, 20, and 25 mg/L RBV, respectively). The length of shoots exposed to the combined treatments was significantly reduced from 2.92 cm (control) to 1.83, 1.85, and 1.99 cm measured in treatments by 15, 20, and 25 mg/L RBV after exposure for 40 days. Leaves became yellow and black during the first 5–35 days due to high temperature, but with development of new leaves, the recovery of shoots could be observed [4].

Begonia (*Begonia × semperflorens* Link & Otto) in vitro cultures were grown on the medium with 20 mg/L RBV and cultured at 38/22 °C for 16 h L/8 h D, for 25 days. Survival rates were about 100% (20 mg/L RBV and 25 days thermotherapy) or less than 30% (30 mg/L RBV and 30 days thermotherapy). Culture of shoots for more than 25 days and over 20 mg/L RBV resulted in the death of the shoots [165].

Different antiviral drugs (20 mg/L RBV, or 20 mg/L 5-Azacytidine (AZA) or 30 mg/L 3-Deazauridine (DZD) were applied for potato (*Solanum tuberosum* L., ‘Baraka’) virus elimination of PVY, and shoots were cultured at 37 ± 2 °C, for 30 days: 83.3% of plantlets survived and regenerated in each treatment. However, plants cultured on the medium with RBV were less vigorous, and slow root and shoot development were observed [122].

Combination of chemo- (15 and 25 mg/L RBV) and thermotherapy (temperature increased gradually up to 37 °C, applied for 20, 30, and 40 ds) was tested for virus elimination of GLRaV-3 from grapevine (*Vitis champinii* Planch). Although all shoots survived the treatments, symptoms of heat stress occurred (discoloration, necrosis, and wrinkles) from the beginning of treatment. However, the lengths of all treated shoots were higher than control shoots in the whole experiments, which was due to thermotherapy, because this phenomenon was only observed on shoots treated by heat independently on application of RBV. Neither RBV level nor heat treatment showed clear tendentious effect on the regenerative capacity of shoots evaluated after five sub-cultures [127].

Sequentially, the treatment of rose (*Rosa* (L.) *hybrida*) by chemotherapy (10, 20, and 30 mg/L RBV) followed by thermotherapy (38/22 °C) for 30 days enhanced the shoot regeneration compared to the control (Table 7). The regeneration rate was dependent on the virus infection, in the control treatments, 33.3%, 40%, and 26.7% regeneration rates were obtained in ArMV, PNRSV, and ArMV with PNRSV co-infected plantlets. However, when 30 mg/L RBV was applied, the regeneration rates increased up to 100% (in ArMV and PNRSV infected plantlets) or up to 90% (in ArMV with PNRSV co-infected plantlets), and lower concentration of RBV also resulted in increased regeneration rates (Table 6). Even though the highest RBV level stimulated the calli formation on the nodal parts of stem, the growth and development of shoots were not inhibited [213].

When chemo-treated (20 mg/L RBV for three weeks) potato (*Solanum tuberosum* L.) shoots were moved to heat chamber (37 ± 1 °C for two weeks) without transfer of shoots onto the new medium, the regeneration rates were highly depended on genotypes. Regeneration rates were 15%, 45%, and 47.5% in PVS infected ‘Kerrs Pink blatt skall’, ‘Hroar Dege’, and ‘Iverpotet/Smaragd’, respectively. The regeneration rates were 27.5% and 32.5% in the PVY and PVS mix-infected ‘Gjernespotet’ and ‘Sverre’, respectively, while and 35% in ‘Gammelraude’, which was mix-infected by PVM and PVS. The highest regeneration rate (72.5%) was observed in ‘Abundance’ explants from plants infected by PVX, followed by the 65% regeneration rate detected in ‘Truls’ (mix-infected by PVY, PVS, and PVX) [59].

Pear cultivars (*Pyrus pyrifolia* Burm, ‘Wonhwang’, ‘Xuehua’, ‘Conference’, ‘Stankimson’, ‘Starcrimson’, ‘Red Bastlett’) were grown on the medium with 25 mg/L RBV for seven days, then exposed to thermotherapy (gradually increased temperature from 27 to 35 °C, for total 40 days). Each shoot survived the combined treatment in ‘Wonhwang’, ‘Conference’, ‘Stankimson’, and ‘Starcrimson’, and high survival rates were observed in ‘Xuehua’ and ‘Red Bastlett’ (both reached 80%). However, the regeneration rates of isolated shoots were lower and very various depending on genotypes. Very low regeneration ability was observed in ‘Conference’ (28.2%), in ‘Stankimson’ (28.2%), and in ‘Starcrimson’ (32.1%), while very good regeneration rates were detected in ‘Wonhwang’ (77.8%), in ‘Xuehua’ (63.9%), and in ‘Red Bastlett’ (84.6%) [228]. The length of shoots was decreased by treatment combination in all cultivars, especially in ‘Xuehua’, ‘Conference’, ‘Stankimson’, and ’Starcrimson’, resulting in shorter shoots by 0.6 cm compared to the control. However, the shoot proliferation was significantly stimulated by treatment resulting in 2.5, 2.6, 3.5, and 1.9 multiplication rates in ‘Xuehua’, ‘Conference’, ‘Stankimson’, and ‘Starcrimson’, respectively. According to the earlier experimental results, the authors assumed that the lower survival rates of ‘Xuehua’ and ‘Red Bastlett’ were due to their higher sensitivity to high temperature, since the low RBV level was used. They also found that the higher shoot proliferation was due to the stimulating effect of RBV applied in low concentration [228].

### 7.2. Thermotherapy Combined with Cryotherapy

During the elimination of Raspberry bushy dwarf virus (RBDV) from raspberries (*Rubus idaeus* L., ‘Z13’) by application of thermotherapy followed by cryotherapy, it was observed that the freezing tolerance of cells decreased after the heat treatment, maybe because during heat treatment the cells and their vacuoles enlarged, which can lead to higher rate of dead cells due to their higher water content. As a result, this combined treatment caused a very low survival rate (0–48%), with 0–60% regeneration capability. Moreover, the survival and regeneration rates decreased as duration of heat treatment increased (Table 7). However, 35% of regenerated shoots were free of RBDV, which is a good result considering that RBDV previously was found to be very difficult to eliminate [26]. During post-culture of regenerated shoots, the phenomenon of chlorosis occurred, and some of the affected shoots died, but addition of Fe-EDTA at 50 mg/L to the post-culture medium solved this problem by prevention chlorosis [26].

Thermotherapy and cryotherapy were also combined in antiviral experiments with apple (*Malus domestica* Borkh.). It was found that after four weeks of heat treatment, several cells in the upper layer of the apical dome (AD) region and some cells in between the first and the third leaf primordium (LP1–3) region survived, while the other cells in the shoot apex died in ‘Gala’. In the ‘Ruixue’ variety, some cells also survived in the LP4 region. Regeneration rates of shoot tips decreased from 55.5% to 20% as the heat period increased from two weeks to six weeks (Table 7). The size of isolated shoot tips also influenced the regeneration rates (shoot regenerated in the rate of 11.1%, 46.7%, and 49.5%, when shoot tips were 1.5 mm with 2–3 LP, 1.5 mm with 4–5 LP, or 2.0 mm with 5–6 LP, respectively). Heat treated shoots often showed yellow discoloration, especially at the base. After cryotherapy, some shoot tips died, about 10% of them formed calli without shoot development, and the others regenerated to normal shoots in the rates between 33% and 76% [38].

### 7.3. Chemotherapy Combined with Cryotherapy

Potato (*Solanum tuberosum* L., ‘Tamyr’, ‘Nartau’, ‘Narly’, ‘Aul’, ‘Astana’, and ‘Nikitka’) shoot tips were collected from plantlets grown on medium with 100 mg/L RBV during three sub-cultures (each lasted for 45 days), and they were cryo-treated by using PVS-2-vitrification protocol. The regeneration rates depended on the genotypes; they were 30% (‘Astana’), 25% (‘Nikitka’), 50% (‘Narly’), and 60% (‘Aul’). The regeneration rate also varied by the number of sub-cultures on RBV medium. The regeneration rate increased as the number of sub-cultures increased in ‘Tamyr’ as follows: 30%, 35%, and 55% after the first, second, and third sub-cultures. Similar results (15%, 50%, and 50%, respectively) were obtained with ‘Nartau’ [229].

### 7.4. Chemotherapy Combined with Electrotherapy

In experiments with potato plants (*Solanum tuberosum* L., ‘Diamond’), the RBV applied alone (20 mg/L) barely reduced the regeneration rate, and even electrical treatment (especially direct application) enhanced it in lower currents and shorter exposure times (see Table 4). However, the combined sequential treatment (first electrotherapy and then culture on RBV-containing medium) caused a significant decrease in the regeneration rates. Regeneration rates of 45.5% (direct treatment) and 30% (indirect treatment) observed in control treatments decreased to 24–34.3% and 27–0%, respectively [121].

### 7.5. Suggestions for Application of Combined Treatments

The sensitivity of species and genotypes to combined treatments can be various, and the occurrence of stress symptoms during treatments largely depends on applied treatments. The most frequently used combinations involved thermotherapy, and (until now) the heat treatment has been found to have more stress effect on plants than other applied treatments combined with it. Both the temperature and the duration of heat exposure can significantly affect the survival and/or regeneration rates. Thus, applications, which are suggested to decrease the harmful effects of thermotherapy (pre-treatments, alternate temperature, etc.) can also be used (see Thermotherapy chapter).

In general, when thermotherapy was combined with chemotherapy, the stress effect of antiviral agents was much smaller compared to the effect of heat, maybe due to their low level (in general, 10–30 mg/L) applied in medium. Moreover, when thermotherapy precedes the cryotherapy, the sensitivity of plants to freezing damages can increase significantly, which makes obtaining survivors difficult. Therefore, possibilities of improving the freezing tolerance of explants during cryotherapy should be considered (see Cryotherapy chapter).

However, the pre-treatment of mother plants, e.g., cold acclimation, may not be an option that can be realized. During post-culture of explants isolated from treated plants, the optimization of medium can increase the regeneration rate. Combination of different treatments, e.g., chemotherapy with electrotherapy, may amplify adverse effects that may not be significant individually.

**Table 7:** Experimental details of combined treatments.

Plant Species, Cultivar, Virus	Methods	Survival and/or Regeneration [Reference]
Potato, *Solanum tuberosum* L., ‘Baraka’, PVY	Chemo- and thermotherapy: Single node cuttings grown on MS with 1 mg/L thiamine, 100 mg/L myo-inositol, 2 mg/L glycine, 30 g/L sucrose; 8.0 g/L agar; 0.001 mg/L NAA, 1.0 mg/L KIN, 0.1 mg/L GA_3_, with 20 mg/L RBV, or 20 mg/L AZA or 30 mg/L DZD, cultured at 37 ± 2 °C, at 16 h L., 110 µmol m^−2^ s^−1^, for 30 days. After treatment plantlets acclimated and potted.	83.3% survived and regenerated in each treatment. Plants cultured on medium with RBV were less vigorous, slow root and shoot development. [122]
Begonia, *Begonia × semperflorens* Link & Otto, PNRSV	Chemo- and thermotherapy: Shoots cultured on 1/2 MS medium with 20 or 30 mg/L RBV, and at 38/22 °C for 16 h L/8 h D, for 25 or 30 days.	Survival rate about 100% (20 mg/L RBV and 25 days thermotherapy) or less than 30% (30 mg/L RBV and 30 days thermotherapy). Culture of shoots more than 25 ds, and over 20 mg/L RBV resulted in death of shoots. [165]
Myrobalan, *Prunus cerasifera* var. *divaricata* Borgh, ACLSV, PNRSV, Plum, *Prunus domestica* L., ‘Empress’, PNRSV, Sweet cherry, *Cerasus avium* L., Moench., ‘Early Rivers’, PDV	Chemo- and thermotherapy: ST (1.0 cm) grown on MS with 5.0 µmol/L BA, 0.5 µmol/L IBA, and RBV in concentrations of 10, 25, 50 or 100 mg/L. Culture: temperature gr. increased from 28 to 36 °C within a week, and kept at 36 °C for four weeks. Post-culture: shoots on the fresh medium 24/21 °C for 4 weeks, then shoots on the rooting medium (2 mg/L IBA). Potted and kept in greenhouse.	Survival rate on medium with 10, 25, 50 or 100 mg/L. Myrobalan: 83.3%/73.3%/63.3%/60%. ‘Empress’: 73.3%/66.7%/33.3%/6.7%. ‘Early Rivers’: 100%/100%/84%/60%. [135]
Raspberries, *Rubus idaeus* L. ‘Z13’ and virus-free cultures of line TTA-508 RBDV	Thermo- and cryotherapy: Shoots ˃ 2 cm on MS with 100 mg/L myo-inositol, 30 g/L sucrose, 0.5 mg/L BA, 0.05 mg/L IBA, 3.5 g/L agar, 1.2 g/L Gelrite. Culture: at 22 ± 2 °C, 16 h, 45 μE s^−1^ m^−2^ for 3 d. Then at 16 h L, 38 °C/8 h D, 26 °C; for 21–42 ds. Then 1.0 mm ST isolated for cryotherapy: stabilized on MS with 2.5 g/L AC (2 ds); encapsulation (2.5% Na-alginate, 2 M glycerol, 0.4 M sucrose in 0.1 M CaCl_2_ solution with 2 M glycerol, 0.4 M sucrose). Vitrification: on MS with increasing level of sucrose (0.25–0.75 M) for 3 ds) (pre-culture), then treated with 2 M glycerol + 0.8 M sucrose (90 min), dehydration with PVS-2 (24 °C, 180 min). Surface drying, cryotube in LN for 1 h, thawed in a water bath (40 °C, for 3 min). Washing (MS with 1 M sucrose, 20 min), post-culture on MS with 50 mg/L Fe-EDTA. D, 22 ± 2 °C for 3 ds, then regeneration 22 ± 2 °C, 16 h, 45 μE s^−1^ m^−2^.	Survival rates/regeneration rates after 2 wks post-culture: control: 85%/78%; 21 d heat + Cryo: 48%/60%; 28 d heat + cryo: 36%/40%; 35 d heat + cryo: 20%/30%; 42 d heat + cryo: 0%/0%. [26]
Potato, *Solanum tuberosum* L., ‘Diamond’ PVY	Chemo- and electrotherapy: Electric current treatment: Stem with 5 nodes: directly connected to the electrodes: 5; 10; and 15 mA for 5- or 10-min. Stem with 2 nodes: in the electrophoresis chamber with NaCl solution, 5; 10; and 15 mA (indirectly). ST (1.0 mm) from AX buds from treated stems: meristem culture on MS with 0.1 mg/L IAA, 0.2 mg/L GA_3_, with 20.0 mg/L RBV, 30 g/L sucrose, and 7.0 g/L agar. Culture: 25 ± 2 °C, 4–6 wks, then multiplication (the same medium without IAA) and rooting (the same medium with 0.04 mg/L KIN): 25 ± 2 °C, 16 h, 2500 lux. Potted, acclimatized, 3 wks.	Regeneration rates (mA/min.): 5/5; 5/10; 10/5; 10/10; 15/5;/15/10: Directly: with RBV: control: 45.5%; 34.3%; 33.3%; 31.3%; 27.6%; 25%; 24%. Indirectly: with RBV: control: 30%; 27%; 27%; 24.1%; 18.5%; 18.3%; 0%. [121]
Sand pear, *Pyrus pyrifolia*, Burm. ‘Jinshui no. 2’, ACLSV, ASGV	Chemo- and thermotherapy: Shoots cultured on MS with RBV in 15, 20, and 25 mg/L concentration, cultured at 24 ± 1 °C, 16 h L, 40 µmol m^−2^ s^−1^ for 2 ds, then at gradually raised temperature from 24 ± 1 °C to 35 ± 0.5 °C within 4 d at 16 h, 40 µmol m^−2^ s^−1^ for further 40 ds. Meristem (1.0–0.5 mm) from five main shoots and axillary shoots were cultured on MS. 2 cycles of sub-culture.	Each shoot survived. Regeneration rates of isolated meristems: control: 88.3%; RBV 25 mg/L + 35 °C for 30 ds: 87.1%; RBV 25 mg/L + 35 °C for 35 ds: 79.2%; RBV 25 mg/L + 35 °C for 40 ds: 78.2%; RBV 20 mg/L + 35 °C for 40 ds: 79.8%; RBV 15 mg/L + 35 °C for 40 ds: 84.3% in average of meristems with 0.5- and 1-mm length). [4]
Apple, *Malus domestica* Borkh, ‘Xinhongjiangjun’, ACLSV, ASGV, ASPV	Chemo- and thermotherapy: In vitro plantlets on MS with 1.0 mg/L BA, 0.1 mg/L NAA, 30 g/L sucrose, 5.6 g/L agar. RBV into medium in 15 or 25 mg/L. Culture: 24 ± 1 °C, 16 h L, 2000 lux, for 2 ds. Then the temperature was raised gradually to 34 ± 0.5 °C, 36 ± 0.5 °C, and 38 ± 0.5 °C. The duration of thermotherapy: 20 days.	Survival rates of shoots after treatments/survival rates of isolated AP meristems/AX meristems (15/25 RBV mg/L): 34 °C: (100/100%)/(73.3%/70.0%)/(78.8%/72.9%); 36 °C: (100%/90%)/66.75/59.3%)/(83.3%/100%); 38 °C: (40%/46.7%)/(16.7%/0%)/(0%/1%). [140]
Rose, *Rosa (L.) hybrida*, PNRSV, ArMV	Chemo- and thermotherapy: Nodal cuts on MS with 0.4 mg/L NAA, 0.4 mg/L BA. RBV added to the medium in concentration of 10, 20, and 30 mg/L. Culture at 16 h L/8 h D, 38/22 °C for 30 days. Then rooting of ½ MS with 6.0 mg/L IAA.	Regeneration rates in control/10/20 and 30 mg/L RBV treatments: ArMV infected: 33.3%/43.3%/50%/100%; PNRSV infected: 40%/60%/60%/100%; ArMV + PNRSV co-infected: 26.7%/36.7%/26.7%/90%. [213]
Potato, *Solanum tuberosum* L., ‘Tamyr’, ‘Nartau’, ‘Narly’, ‘Aul’: PVM and PVS ‘Astana’, ‘Nikitka’: PVM	Chemo- and cryotherapy: Shoots on MS with 100 mg/L RBV for 45 ds, 3×. Shoot tip (1.5–2.0 mm) for cryotherapy: PVS-2-vitrification protocol, ST pre-cultured on MS with 0.3 M sucrose for 1 d at 24 °C, 16 h L, 40 μmol m^−2^ s^−1^, then ST in 1.2 mL cryovials with 2 M glycerol, 0.4 M sucrose for 20 min at 24–25 °C, then exposure to PVS-2 for 30 min (24–25 °C). LN: 15–20 min. Warming: 45 °C water-bath for 1 min, then in 22 °C water for 1 min. ST: rinsed 2x with liquid MS with 1.2 M sucrose, ST onto MS with 2.0 mg/L calcium D-pantothenate, 3.0 g/L agar 1.25 g/L Gelrite, 30 g/L sucrose, darkness for 1 week, then normal conditions.	Regeneration rates after 1st, 2nd, and 3rd sub-cultures: ‘Tamyr’: 30%/35%/55%; ‘Nartau’: 15%/50%/50%. Regeneration rates after 3rd sub-culture of other cultivars: ‘Astana’: 30%; ‘Nikitka’: 25%; ‘Narly’: 50%; ‘Aul’: 60%. [229]
Apple, *Malus domestica* Borkh, ‘Gala’, ‘Fuji’, Ruixue’, ‘Nongguo 25’, *Malus pumila paradisiaca* L., ‘M9’ ASGV	Thermo- and cryotherapy: MS + 0.25 mg/L BA, 2-wk-old shoot grown at 36/32 °C, 16 h, 50 µE s^−1^ m^−2^, for 0, 2, 4, and 6 wks. After 4 wks ST 1.5 mm (2-3 LP), 1.5 mm (4-5 LP), 2.0 mm (5-6 LP). Pre-culture: ST on MS + 0.25 mg/L BA for 1 d, then liquid MS + 2 M glycerol, 0.8 M sucrose 1 d, then vitrification: PVS-2 at RT 40 min, after dehydration: 2.5 µL PVS-2 droplets, directly LN for 30 min, rewarm, unloading solution 1.2 M sucrose, in MS at RT, for 20 min. Post-culture: MS + 0.25 mg/L BA, D for 3 ds, passage every 16–24 h to fresh medium, culture at 24 ± 2 °C, 16 h, 50 µE s^−1^ m^−2^, for 4 wks, sub-culture 4 wks, rooting 0.5 mg/L NAA, for 4 wk, planted to soil.	Regeneration rates after cryo-treatment of ‘Gala’ ST from different period of heat: 0 wk: 62.2%; 2 wk: 55.5%; 4 wk 44.4%; 6 wk 20.0%. Regeneration rates of different sized ST: 1.5 mm (2–3LP): 11.1%; 1.5 mm (4–5LP): 46.7%; 2.0 mm (5–6LP): 49.5%. [38]
Potato, *Solanum tuberosum* L., ‘Truls’, ‘Kerrs Pink blatt skall’, ‘Gammelraude’, ‘Abundance’, ‘Gjernespotet’, ‘Hroar Dege’, ‘Iverpotet/Smaragd’ and ‘Sverre’, PVY, PVM, PVS, PVX	Chemo- and thermotherapy: Shoot segments (0.5 cm) with AP bud from three-week-old cultures, on MS with 30 g/L sucrose, 1 g/L casein, 0.5 mg/L IBA, 20 mg/L RBV and 9 g/L Bacto agar. Cultured at 20 ± 2 °C, 16 h L, 50 µmol s^−1^m^−2^ for 3 wks, then 37 ± 1 °C, 16 h L, 50 µmol s^−1^ m^−2^ for 2 wks.	Regeneration rates: ‘Truls’: 65%, ‘Kerrs Pink blatt skall’: 15%, ‘Gammelraude’:35%, ‘Abundance’: 72.5%, ‘Gjernespotet’: 27.5%, ‘Hroar Dege’: 45%, ‘Iverpotet/Smaragd’: 47.5%, ‘Sverre’: 32.5%. [59]
Pear, *Pyrus pyrifolia* Burm, ‘Wonhwang’, ‘Xuehua’, ‘Conference’, ‘Stankimson’, ‘Starcrimson’, ‘Red Bastlett’, ASPV	Chemo- and thermotherapy: ST (1 cm) from MS with RBV 25 mg/L. Culture: 24 °C for 7 ds. Thermotherapy: T increased by 3 °C/d, up to 27, 30, 33, or 35 °C, 16 h L., 2000 lux, for 40 ds. Meristem tips (1.0 mm) were cultured on MS.	Survival rates of treated shoots/isolated ST in cultivars: ‘Wonhwang’: 100%/77.8%; ‘Xuehua’: 80%/63.9%; ‘Conference’: 100%/28.2%; ‘Stankimson’: 100%/28.2%; ‘Starcrimson’: 100%/32.1%; ‘Red Bastlett’: 80%/84.6%. [228]
Grapevine, *Vitis champinii* Planch, GLRaV-3	Chemo- and thermotherapy: Shoots on ½ MS with 15 or 25 mg/L RBV, and heat treatment: T increased gr. to 37 °C, cultured total for 40 days, sub-culture 5×.	100% survival in each treatment. Regeneration rates of isolated ST: RBV 25 mg/L + heat for 20 ds: 35.5%. RBV 15 mg/L + heat for 20 ds: 53.3%; RBV 15 mg/L + heat for 30 ds: 25.8%; RBV 15 mg/L + heat for 40 ds: 50%. The shoot lengths after 10/20/30/40 days heat treatment: Control: 1.79 cm/2.11 cm/2.14 cm/2.71 cm, RBV 15 mg/L: 2.13 cm/2.51 cm/3.45 cm/3.56 cm; RBV 25 mg/L: 2.11 cm/2.36 cm/-/-. [127]

AC: activated charcoal; ACLSV: Apple chlorotic leaf spot virus; AP: apical; ArMV: Arabis mosaic virus; ASGV: Apple stem grooving virus; ASPV: Apple stem pitting virus; AX: axillary; AZA: 5-Azacytidine (4-amino-1-β-d-ribofuranosyl-5-triazin-2[1H]-one); BA: 6-benzyladenine; d(s): day(s); D: darkness; DZD: 3-Deazauridine (4-hydroxi-1-β-d-ribofuranosyl-2 [1H] pyridone); Fe-EDTA: Ferric Ethylene diamine-tetra acetic Acid; GA_3_: gibberellic acid; GLRaV-3: Grapevine leafroll-associated virus; gr: gradually; h(s): hour(s); IAA: β-indolylacetic acid; IBA: Indole-3-butyric acid; KIN: kinetin; L: light; LN: liquid nitrogen; LP: leaf primordium(a); min: minute; MS: Murashige-Skoog medium [62]; NAA: α-naphthylacetic acid; PDV: Prune dwarf virus; PNRSV: Prunus necrotic ring spot virus; PVM: Potato virus M; PVS: Potato virus S; PVS2: PVS2 solution contains 30% (*w*/*v*) glycerol, 15% (*w*/*v*) ethylene glycol, 15% (*w*/*v*) dimethylsulfoxide (DMSO) and 0.4 M sucrose in MS medium (pH 5.8) [26]; PVX: Potato virus X; PVY: Potato virus Y; RBDV: Raspberry bushy dwarf virus; RBV: Ribavirin (1-β-d-ribofuranosyl-1, 2,4 triazone-3-carboxamide) Virazole^®^ a synthetic broad-spectrum antiviral nucleoside; RT: room temperature; SCMV: Sugarcane mosaic potyvirus; ST: shoot tip; T: temperature; wk(s): week(s).

## 8. Conclusions

Since there is no simple way to control viral diseases in plants, the virus elimination methods are of a great importance not only in production of virus-free propagation material of different plant species, but in preservation of valuable genetic resources in plant breeding work. These methods are based on application of in vitro tissue culture techniques, including meristem isolation applied alone, or together with other treatments. Because almost every virus eradication process damages the host plants as well, this review aimed to collect and report the harmful effect of methods and to show possibilities to mitigate them.

We can choose from several methods for virus elimination processes, but the properties of virus to be eliminated, characteristics of host plant, especially the interactions between them, and all the factors determining the way have to be applied. Virus properties, including its type, distribution and concentration in plants, configuration, and resistance or sensitivity for heat and chemicals should be taken into account. From the perspective of the plant, species characteristics should also be considered, including its habitat, seasonal growth cycles, and environmental requirements (temperature, day length, etc.). Moreover, within a species, the genotype plays a very important role in response to virus eradication treatment. In addition, the physiological state of plants, e.g., the seriousness of disease caused viral infection should be considered, because it can modify the sensitivity of plants to treatments. However, with any kind of method we choose, we must take into account that some stress will occur on the plant during application. Injury, high or low temperatures, electric current, antiviral drugs, and other chemicals added to the medium are all stress factors. Both the survival and regeneration ability of the treated plant parts should be enhanced by application of the properly selected methods. Adequate pre-conditioning of mother plants, pre-treatments of explants, or timing of meristem/shoot tip isolation can increase the survival and regeneration rate of treated explants. Use of appropriate PGR content, absorbent, or/and antioxidant in the medium can also improve the outcome of treatments. Environmental conditions, such as temperature and light conditions, both affecting the enzymatic pathways, should also be fitted to the requirements of cultured plant parts. Moreover, the frequent transfer also can help the survival of explants.

In the future, the antiviral therapies whose effects are based on directly the destruction of viruses should be preferred, and the importance of these methods is likely to increase. One of the most perspective therapies is electrotherapy, whose application can be extended in the future, especially if we consider that apart from high efficacy in virus elimination, the electric treatment increased the regeneration rate and stimulated shoot growth, multiplication rate, and other developmental processes for several plant species [121,149,151]. Another possible future perspective is using chemicals, even natural compounds, like melittin, which destroy the virus particles before they can join to the plant cell and thereby cause the loss of the infectivity of the viruses. Cryotherapy also seems to be an effective method to obtain virus-free shoots in a large proportion and rapidly.

Improvements of virus eradication methods are of a great importance to increase effectiveness, and it is necessary to develop new practices, such as new pre-treatment technics, to adopt and extend the applied therapies to more plant species. The information and knowledge summarized in this review can be utilized in virus elimination practice of several plant species grown as food, ornamental, and industrial crops.

## Data Availability

Not applicable.
